# Karakum desert: a unique source of cultivable novel and rare actinomycetes with a remarkable biosynthetic potential

**DOI:** 10.1007/s11274-025-04399-3

**Published:** 2025-06-24

**Authors:** Hayrettin Saygin, Nevzat Sahin, Michael Goodfellow

**Affiliations:** 1 Department of Molecular Biology and Genetics, Faculty of Sciences, Ondokuz Mayis University, Samsun, 55139 Turkey; 2 School of Natural and Environmental Sciences, Newcastle University, Ridley Building 2, Newcastle upon Tyne, NE1 7RU UK

**Keywords:** Selective isolation, Taxogenomics, Biodiversity, Genome-mining, Biosynthetic gene clusters, Stress genes

## Abstract

A culture-based strategy was used to isolate and screen representative actinomycetes from six sampling sites in the Karakum Desert, Turkmenistan. A total of 459 actinomycete isolates were obtained using 16 selective media, and 270 representative strains were subjected to 16 S rRNA gene sequencing. Comparative 16 S rRNA gene sequence analyses on colour-group representatives led to their assignment to 17 genera with validly published names which included many isolates assigned to novel or putatively novel species including ones belonging to rare genera, such as *Actinocorallia*, *Actinomadura*, *Jiangella* and *Nonomuraea*. Mining of whole-genome sequences of 32 isolates which formed distinct lineages in phylogenomic trees revealed biosynthetic gene clusters predicted to encode for many novel specialized metabolites, notably antibiotics. The genomes of most of these isolates included genes associated with the promotion of plant growth while bioinformatic analyses of stress-related genes provided on insight into how filamentous actinomycetes have adapted to harsh environmental conditions in the Karakum Desert. This extensive bioprospecting campaign shows that the Karakum Desert is a unique source of novel, rare and gifted filamentous actinomycetes with the ability to synthesise new specialized metabolites needed to address key existential issues facing humankind, especially, the urgent need to find a new generation of therapeutic antibiotics to control multidrug-resistant microbial pathogens and compounds that protect and promote plant growth.

**Supplementary Information:**

The online version contains supplementary material available at 10.1007/s11274-025-04399-3.

## Introduction

Natural products remain the most prolific source of pharmaceutically interesting biomolecules (Newman and Cragg 2020) needed to control multidrug-resistant microbial pathogens and to treat patients with life-threatening diseases such as cancer (Sivalingam et al. 2019). Filamentous actinomycetes classified in the phylum A*ctinomycetota* (Oren and Garrity 2021) are, and remain, a unique source of novel antibiotics (Takahashi and Nakashima 2018). Streptomycetes are especially gifted *sensu* Baltz (2017) as they have large genomes rich in natural product – biosynthetic gene clusters (NP – BGCs) many of which encode for new and uncharacterised antibiotics (Sivakala et al. 2021). Other gifted filamentous actinomycetes include ones classified in the genera *Amycolatopsis* (Sangal et al. 2018). *Micromonospora* (Carro et al. 2019a), *Nocardia* (Männle et al. 2020) and *Salinispora* (Jensen et al. 2015). Rare and novel actinomycetes are an asset in the search for novel bioactive compounds using emerging technologies, notably genome mining, metabolic engineering, metabolomics, proteomics and transcriptomics (Sekurova et al. 2019; Atanasov et al. 2021; Ossai et al. 2022). Questions arising from these developments include “how and where to look for novel gifted actinomycetes”.

Extreme biomes are promising sources of gifted filamentous actinomycetes as harsh abiotic conditions select for novel actinomycetes with unexplored chemical diversity that can lead to the discovery of new bioactive compounds (Rateb et al. 2018; Sayed et al. 2020a). The genomes of some gifted filamentous actinomycetes from extreme habitats contain clade and species-specific NP-BGCs (Adamek et al. 2018; Carro et al. 2018; Nouioui et al. 2019); a development which underpins the importance of sound classification in bioprospecting campaigns, notably taxonomic approaches to drug discovery (Świecimska et al. 2023). Genome-based taxonomies also bring greater precision to bioprospecting and ecological studies (Nouioui et al. 2018; Sangal et al. 2018), as do genomic-based metrics used to delineate generic, species and subspecific boundaries (Meier-Kolthoff et al. 2014; Sant’Anna et al. 2019; Thompson et al. 2020). Further, the detection of stress-related genes in genomes of actinomycetes isolated from extreme habitats provide genomic insights into how they have adapt to extreme environmental conditions (Busarakam et al. 2016; Abdel-Mageed et al. 2020).

Until recently, little was known about the numbers, types and distribution of actinomycetes in deserts even though desert biomes account for about 20% of the Earth’s landmass around 7% of which is hyper-arid (Neilson et al. 2012; Mohammadipanah and Wink 2016). Desert habitats are an especially rich source of novel and rare filamentous actinomycetes that produce new specialized (secondary) metabolites, such as anticancer compounds, antibiotics, immunosuppressive agents and enzyme inhibitors (Xie and Pathom-aree 2021). Small numbers of taxonomically diverse, bioactive, filamentous actinomycetes are characteristic of the Atacama (Goodfellow et al. 2018), Chihuahuan (Arocha-Garza et al. 2017), Kazakhstan (Ziyat et al. 2019), Mongolian (Kurapova et al. 2012), Gurbantunggut (Li et al. 2021), Mojave (McHugh et al. 2017), Taklamakan (Liu et al. 2021), Thar (Masand et al. 2018) and Sahara (Meklat et al. 2020) deserts, polar desert soils (Ji et al. 2022), the desert ecosystem of the Qinghai-Tibet plateau (Ding et al. 2013) and the cold desert of the Leh Ladakh region of Jammu and Kashmir (Yadav et al. 2015).

Actinomycetes from Central and South Asian deserts have rarely featured in biotechnological or ecological studies (Xie and Pathom-aree 2021; Xie et al. 2022). This also applies to the Karakum Desert, one of the largest deserts in the world and the hottest in Central Asia (Ghassemi and Garzanti 2019). Recently, small numbers of filamentous actinomycetes isolated from undisturbed habitats in the Karakum Desert were described as novel *Jiangella* (Saygin et al. 2020a), *Kribbella* (Saygin et al. 2019a), *Micromonospora* (Saygin et al. 2020c), *Nonomuraea* (Saygin et al. 2020d, 2021a), *Saccharopolyspora* (Saygin et al. 2021b), *Spongiactinospora* (Saygin et al. 2019b; Ay et al. 2020) and *Streptomyces* (Saygin et al. 2020b) species thereby providing additional evidence that neglected desert biomes are a source of gifted filamentous actinomycetes with the potential to produce new drug leads and compounds that promote and protect plant growth (Nouioui et al. 2019; Ebrahimi-Zarandi et al. 2022).

In this study, representative amycelial and filamentous actinomycete colonies from selective isolation plates inoculated with environmental samples from six locations in the Karakum Desert were assigned to colour-groups. 16 S rRNA gene sequence analyses were carried out on representatives of the colour-groups to determine their taxonomic status and the resultant phylogenetic data used to establish the identity of isolates assigned to the colour-groups. Whole-genome sequences generated for 32 isolates that differed markedly from their phylogenetic neighbours were examined for BGCs predicted to express for specialized metabolites, notably antibiotics, for genes which code for compounds that promote plant growth and stress-related genes. The overall objectives of the study were to determine the taxonomic diversity of culturable actinomycetes, the functional activities and genetic potential of representative isolates, and to generate a high quality library of taxonomically diverse actinomycetes that can be used to address critical contemporary issues facing agricultural, industrial and medical biotechnology.

## Materials and methods

### Karakum desert: geography, biology and sampling sites

**Fig. 1:**
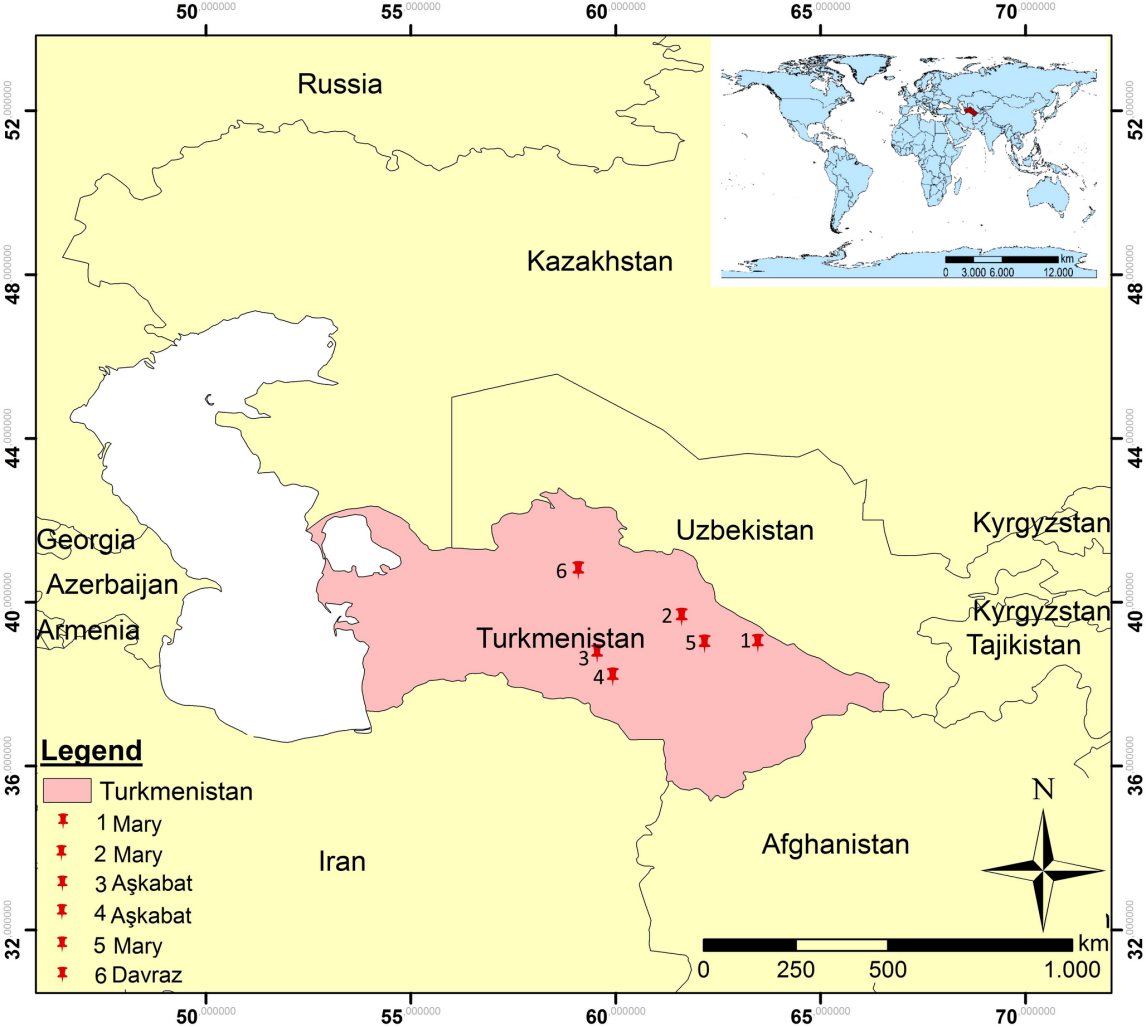
Location of the Karakum Desert sampling sites

**Fig. 2:**
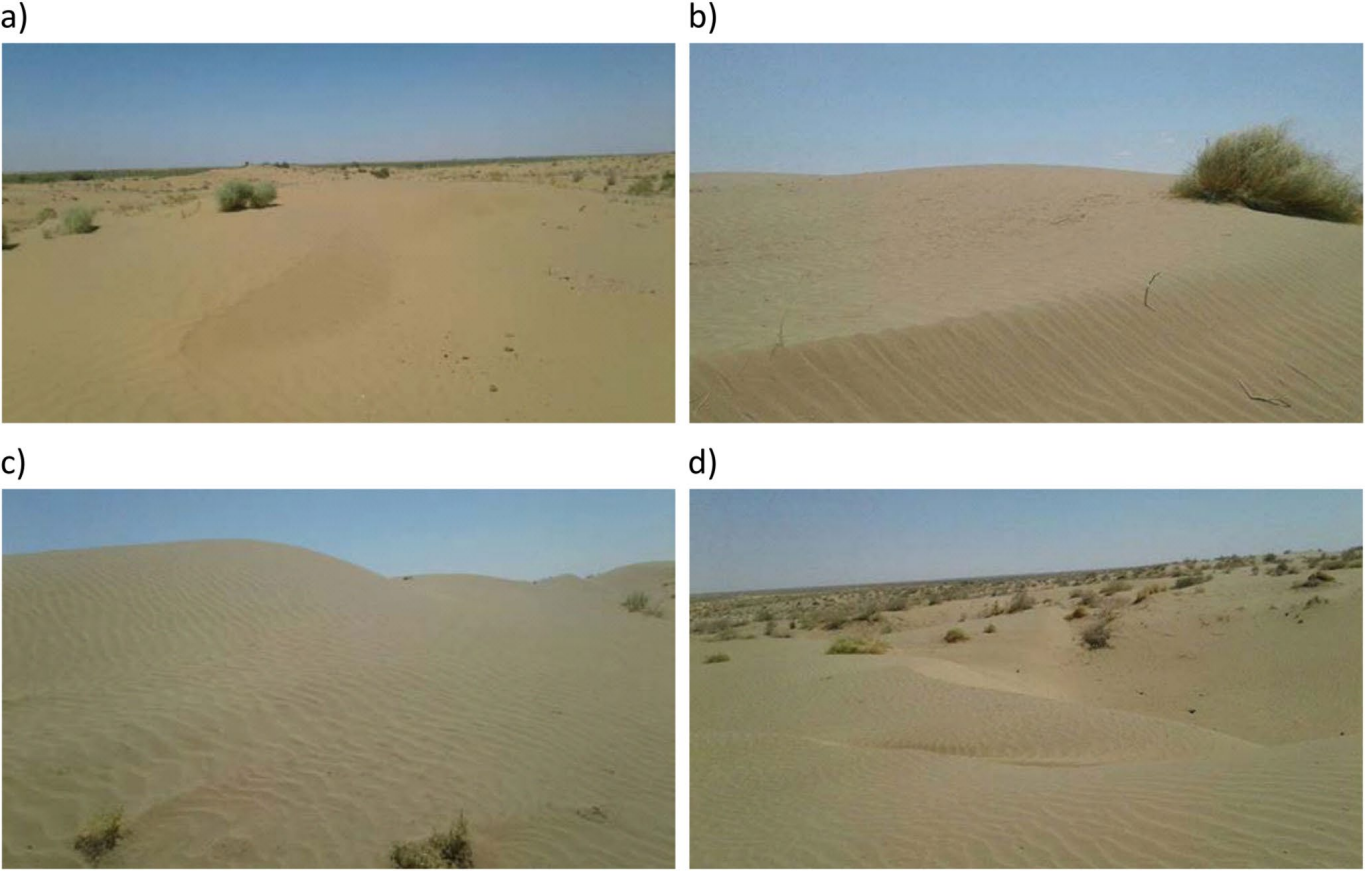
Selected sampling locations in the Karakum Desert; (**a**) Mary (sample no 1), (**b**) Aşkabat (sample no 3), (**c**) Mary (sample no 5) and (**d**) Darvaz (sample no 6)

The Karakum Desert, which covers about 80% of Turkmenistan (Zonn and Esenov 2014), lies between the River Amudarya in the east and the Caspian Sea in the west (Ghassemi and Garzanti 2019), has extensive underground oil and gas reserves (Arbatov and Karatepe 2006). The region has a seasonal climate, cold winters, warmish dry autumns, gentle rain in spring, and very hot cloudless summers (maximum air temperatures 48 °C to 50 °C in July with ground temperatures above 80 °C (Zonn and Esenov 2014). In July 2014, when the environmental samples were taken, the Karakum Desert had temperatures varying between 23 and 38 °C, a monthly precipitation of about 5 mm, and a wind speed of 1–2 m per second (www.weather-and-climate.com). Environmental samples (Table 1) were collected from selected regions of the Karakum Desert (Figs. 1 and 2). About 200 g of subsurface soil (depth 5 cm) was collected aseptically at each of the pristine sites using a spatula sterilized in the field with ethanol, and placed into sterilized polyethylene bags. The samples were brought to the laboratory quickly and stored at 4 °C.

**Table 1:** Sampling sites

Sample no and location	Latitude °*N*	Longitude °E	Altitude	Date of sampling
1. Mary	38°12’31.41”	62°53’37.29”	190 m	06.07.2014
2. Mary	38°57’18.94”	61°29’10.26”	154 m	06.07.2014
3. Aşkabat	38°25’48.54”	58°29’38.60”	86 m	06.07.2014
4. Aşkabat	38°04’06.25”	58°43’10.59”	131 m	06.07.2014
5. Mary	38°03’25.92”	61°50’05.63”	198 m	06.07.2014
6. Darvaz	40°15’38.13”	58°26’20.39”	90 m	06.07.2014

### Physico-chemical analyses of environmental samples

The pH of each of the samples was determined using the procedure of Reed and Cummings (1945) and electrical conductivity measurements after Avery and Bascomb (1982). The moisture content of the samples were determined by drying known amounts to constant weight at 105 °C. The organic matter content of each sample was calculated using the modified Walkley-Black method (Walkley and Black 1934).

### Selective isolation, purification and preservation of isolates

Environmental samples from each location were air-dried at room temperature for 14 days, and 1 gram of each soil sample was suspended in 9 mL of sterile ¼ strength Ringer’s solution (Oxoid) and agitated on a rotary shaker at 160 rpm for 30 min to release microbial cells. The resulting suspensions were then held at 60 °C for 20 min, and serial 10^− 2^ to 10^− 3^ dilutions were prepared using the same diluent. Serial dilutions (200 µl) prepared from each of the samples were spread over the surfaces of 16 isolation media considered to be selective for taxonomically diverse actinomycetes (Table 2). The numbers of presumptive actinomycetes growing on the isolation plates were expressed as the number of colony-forming units (cfu) per gram dry weight soil following incubation at 28 °C for 4 weeks.

**Table 2:** Media used for the selective isolation of actinomycetes

Media	Selective agents	Target organisms	References
Selective medium 1 (SM1) agar	Neomycin (4 µg/ml), nystatin (50 µg/ml), D-sorbitol (10%)	*Amycolatopsis* spp.	Tan et al. (2006)
Selective medium 2 (SM2) agar	Neomycin (4 µg/ml), nystatin (50 µg/ml), D-melezitose (10%)	*Amycolatopsis* spp.	Tan et al. (2006)
Selective medium 3 (SM3) agar	Nalidixic acid (10 µg/ml), novobiocin (10 µg/ml)	*Amycolatopsis* spp.	Tan et al. (2006)
Marine agar	Nalidixic acid (10 µg/ml)	Actinomycetes	ZoBell (1941)
Humic acid-vitamin agar	Nalidixic acid (10 µg/ml), humic acid (1 g/l)	*Streptosporangium* spp.	Hayakawa and Nonomura (1987)
Starch-peptone-yeast extract (M1) agar	Rifampicin (5 µg/ml)	*Salinispora* spp.	Mincer et al. (2002)
Non-sporulating agar	Rifampicin (5 µg/ml)	Rare actinomycetes	Sanglier et al. (1992)
Reasoner’s 2 A (R2A) agar	Nalidixic acid (10 µg/ml)	*Modestobacter* spp.	Reasoner and Geldreich (1985)
Starch-casein agar	Nalidixic acid (10 µg/ml)	*Streptomyces* spp.	Küster and Williams (1964)
Raffinose-histidine agar	Nalidixic acid (10 µg/ml)	Rare *Streptomyces* spp.	Vickers et al. (1984)
Tryptic soy agar	Nalidixic acid (10 µg/ml)	Actinomycetes	Difco (Cat. No 236950)
Oligotrophic agar	Nalidixic acid (10 µg/ml), low carbon and nitrogen content	Rare and uncommon actinomycetes	Jiang et al. (2016)
Minimal medium	Nalidixic acid (10 µg/ml)	Rare and uncommon actinomycetes	Jiang et al. (2016)
Complex medium	Nalidixic acid (10 µg/ml)	*Nocardiopsis* spp.	Chun et al. (2000)
Glycerol-asparagine agar	Nalidixic acid (10 µg/ml)	*Streptomyces* spp.	Shirling and Gottlieb (1966)
Chitin-vitamin agar	Nalidixic acid (10 µg/ml)	*Streptomyces* spp.	Hayakawa and Nonomura (1987)

All of the media were supplemented with cycloheximide (50 µg/ml)

Presumptive actinomycete colonies taken from each of the selective isolation media were subcultured onto yeast extract-malt extract agar (ISP 2) (Shirling and Gottlieb 1966) plates which were incubated at 28 °C for 14 days. Growth from each of the ISP 2 plates was suspended in 25 % (v/v) glycerol held in vials, and the resultant cultures were preserved at -20 °C and − 80 °C. Colonies were assigned to colour-groups based on aerial spore mass, substrate mycelial, and diffusible pigment colours or colony pigments on oatmeal agar plates (ISP 3) using colour charts (Centore 2016).

### Phylogenetic analyses

Isolates representing the colour-groups were shaken individually in ISP 2 broth at 160 rpm for 14 days at 28 °C and the resultant biomass was harvested by centrifugation and washed twice with an equal volume of sterile distilled water. Genomic DNA was extracted using PureLink^®^ DNA Isolation Kits (Invitrogen, USA). PCR-mediated amplification of 16 S rRNA genes was achieved using the universal primers, 27 F and 1525R (Lane 1991). The PCR products were purified and sequenced using an ABI PRISM 3730 XL automatic sequencer with forward primers 27 F (Lane 1991), 518 F (Buchholz-Cleven et al. 1997), MG5F and MG6F (Chun and Goodfellow 1995) and the reverse primer 800R (Chun and Goodfellow 1995).

Chromatogram files in ABI format were checked using Chromas version 1.45, and the primers overlapped to obtain the 16 S rRNA gene nucleotide sequences in FASTA format for each isolate. The resultant 16 S rRNA gene sequences were deposited in GenBank under accession numbers MG770618-…-MG770879 and MK156407-…-MK156414. The 16 S rRNA gene sequences were uploaded onto the EzBioCloud server (Yoon et al. 2017) and pairwise sequence similarities of the nearest phylogenetic neighbours determined. Phylogenetic trees were generated using the MEGA X software package (Kumar et al. 2018) using the neighbour-joining method (Saitou and Nei 1987). The topologies of the resultant trees were evaluated by bootstrap resampling of 1000 replicates after Felsenstein (1985).

### Whole-genome sequencing of selected isolates

Thirty-two isolates (Table S1), which differed markedly from the type strains of their closest phylogenetic neighbours, were grown in ISP 2 broth at 160 rpm for 7 days at 28 °C; the biomass was collected by centrifugation and washed twice with an equal volume of sterile distilled water. The genomic DNA of each isolate was extracted using PureLink^®^ DNA Isolation Kits (Invitrogen, USA). The quantity of the DNA preparations was measured using NanoDrop 2000 (Thermo Scientific, USA). The whole-genome sequences of the isolates were generated using the Illumina HiSeq 2500 next-generation sequencing platform and the ×250 bp paired-end protocol. Assemblies of raw data were achieved using the full Spades assemble strategy on the Bacterial and Viral Bioinformatics Resource Center (BV-BRC) (https://www.bv-brc.org/) (Olson et al. 2023), and the draft genome sequences deposited in the National Centre for Biotechnology Information (NCBI) database (https://submit.ncbi.nlm.nih.gov/) database under the accession numbers (Table S1). The genome sequences were annotated using the Rapid Annotation Subsystem Technology (RAST) server (Aziz et al. 2008), and uploaded onto the Type (Strain) Genome Server (TYGS), a free bioinformatics platform available at https://tygs.dsmz.de (Meier-Kolthoff and Göker 2019), to construct a phylogenomic tree. The presence of BGCs in the genomes of the isolates were detected using the antiSMASH v7.0 (Blin et al. 2023), with default options available at https://antismash.secondarymetabolites.org.

### Antimicrobial screening of representative isolates

Fifty-two isolates, which differed markedly from their phylogenetic neighbours, were assigned to 11 genera. These strains were examined for their ability to inhibit the growth of a panel of bacteria and fungi, namely *Bacillus subtilis* ATCC 6633, *Escherichia coli* ATCC 25,922, *Enterococcus faecalis* ATCC 29,212, *Klebsiella quasipneumoniae* ATCC 700,603, *Pseudomonas aeruginosa* ATCC 27,853, *Staphylococcus aureus* ATCC 25,923, *Aspergillus brasiliensis* ATCC 16,404 and *Candida albicans* ATCC 10,231, using an agar diffusion procedure as described by Balouiri et al. (2016). The resultant preparations were incubated for 24–48 h at optimal temperatures for the wild-type strains, and the activity of the strains was recorded by measuring inhibition zones around the colonies. Inhibition zones with a diameter of ≥ 10 mm, including the well diameter, were considered indicative of antimicrobial activity.

## Results

### Physico-chemical characteristics of environmental samples

All of the samples were moderately alkaline, highly arid with low levels of organic matter and small electrical conductivity values (Table 3). The samples taken from locations 1 to 4 and 6 had electrical conductivity, moisture, pH and organic matter values that fell within a narrow range, namely 0.12–0.20 (dS/m), 0.20–0.42%, 8.44–8.66 and 0.04–0.20%, respectively. In contrast, the sample from location 5 showed the highest electrical conductivity, and moisture content with the lowest pH and equal lowest total organic matter content.

**Table 3:** Physico-chemical properties of the environmental samples

Sampling site	Total organic matter (%)	pH	Electrical conductivity (dS/m)	Moisture content (%)
1	0,04	8,65	0,14	0.32
2	0,16	8,71	0,12	0.36
3	0,18	8,66	0,13	0.37
4	0,12	8,44	0,14	0.42
5	0,04	8,25	0,75	0.72
6	0,20	8,61	0,20	0.20

### Colony counts, strain selection and assignment of representative isolates to colour-groups

**Fig. 3:**
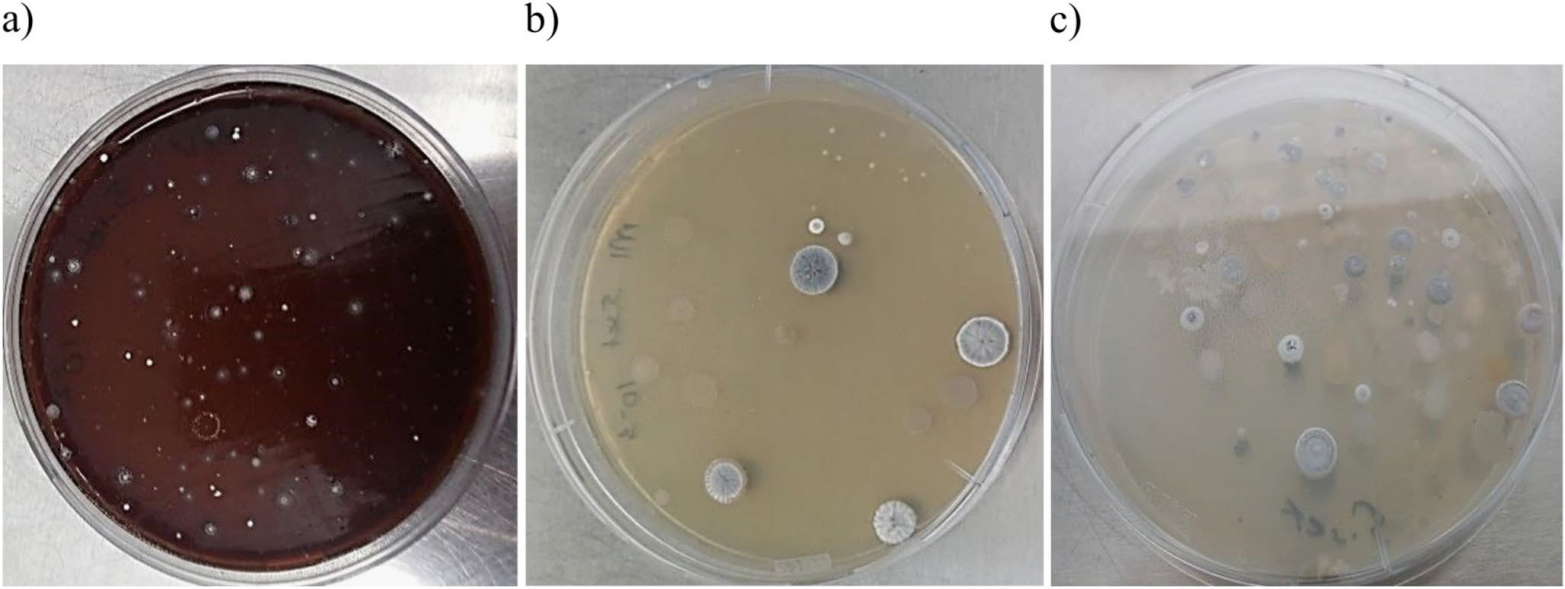
Isolates growing on (**a**) humic acid-vitamin agar plates showing colonies covered by white aerial hyphae, (**b**) M1 agar plates with round colonies carrying a grey aerial spore mass, and (**c**) raffinose-histidine agar plates showing masses of aerial spores following incubation at 28 °C for 28 days

With few exceptions, small numbers of presumptive actinomycete colonies were recorded on the selective isolation plates prepared from environmental samples taken from the sampling locations (Table 4). In all cases the highest counts were recorded from the humic acid-vitamin agar plates with numbers ranging from 55.2 × 10^3^ cfu’s for the sample from location 4 to 217.3 × 10^3^ cfu’s for the sample taken from location 5. In contrast, the lowest counts were found on the three SM media designed to isolate *Amycolatopsis* strains (Tan et al. 2006) and on the tryptic soy agar (TSA) plates. The highest average actinomycete counts were from the samples collected from locations 5 and 6. Four hundred and fifty nine isolates representing distinctive colony types were taken from each of the isolation plates. Of these, 270 representative strains were selected for 16 S rRNA gene sequencing and phylogenetic analysis. Most of these strains produced filamentous colonies covered by either aerial hyphae or by a characteristic aerial spore mass (Fig. 3); presumptive amycelial actinomycete colonies were selected based on colony pigmentation.

**Fig. 4:**
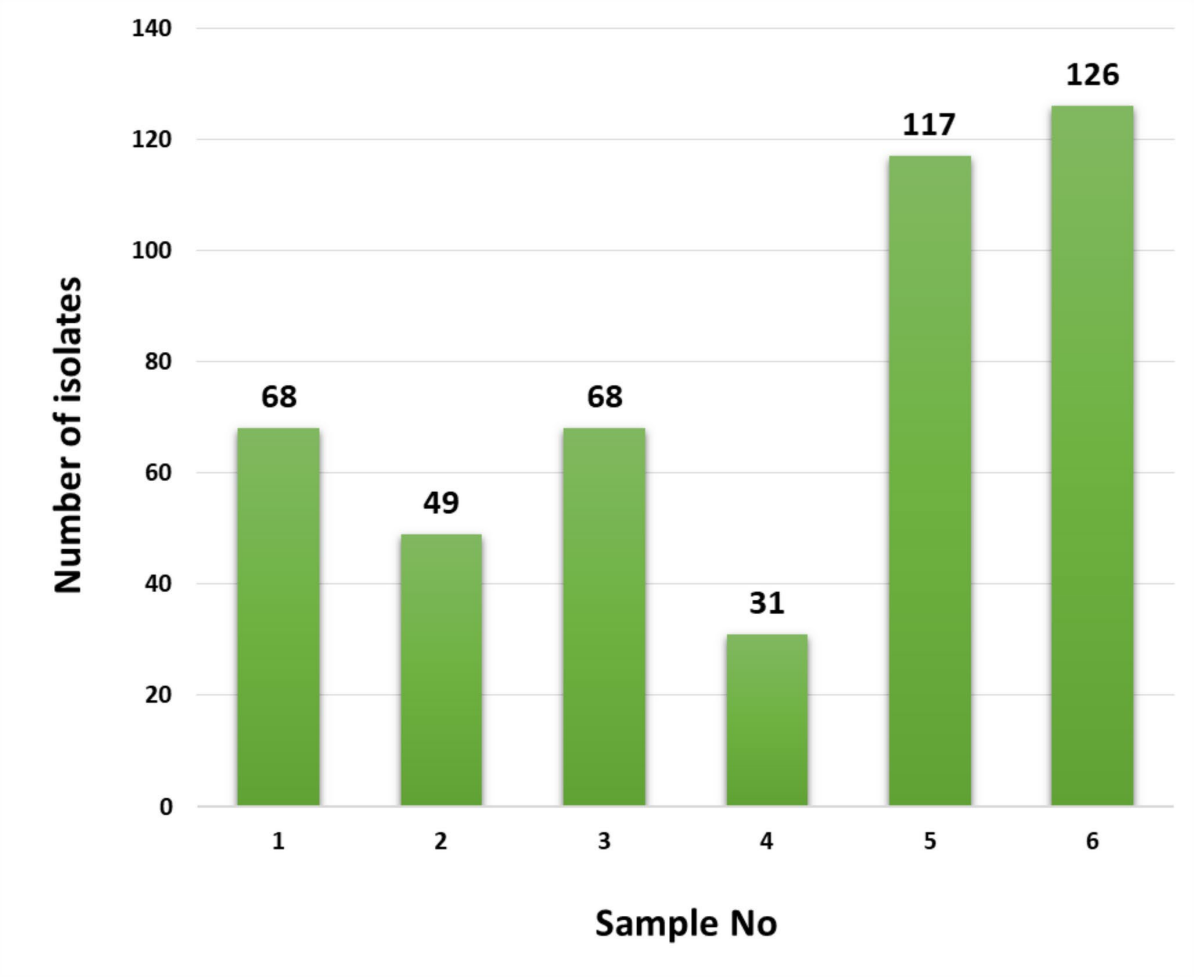
Numbers of strains taken to represent the different colony types of actinomycetes from sampling locations 1 to 6

**Fig. 5:**
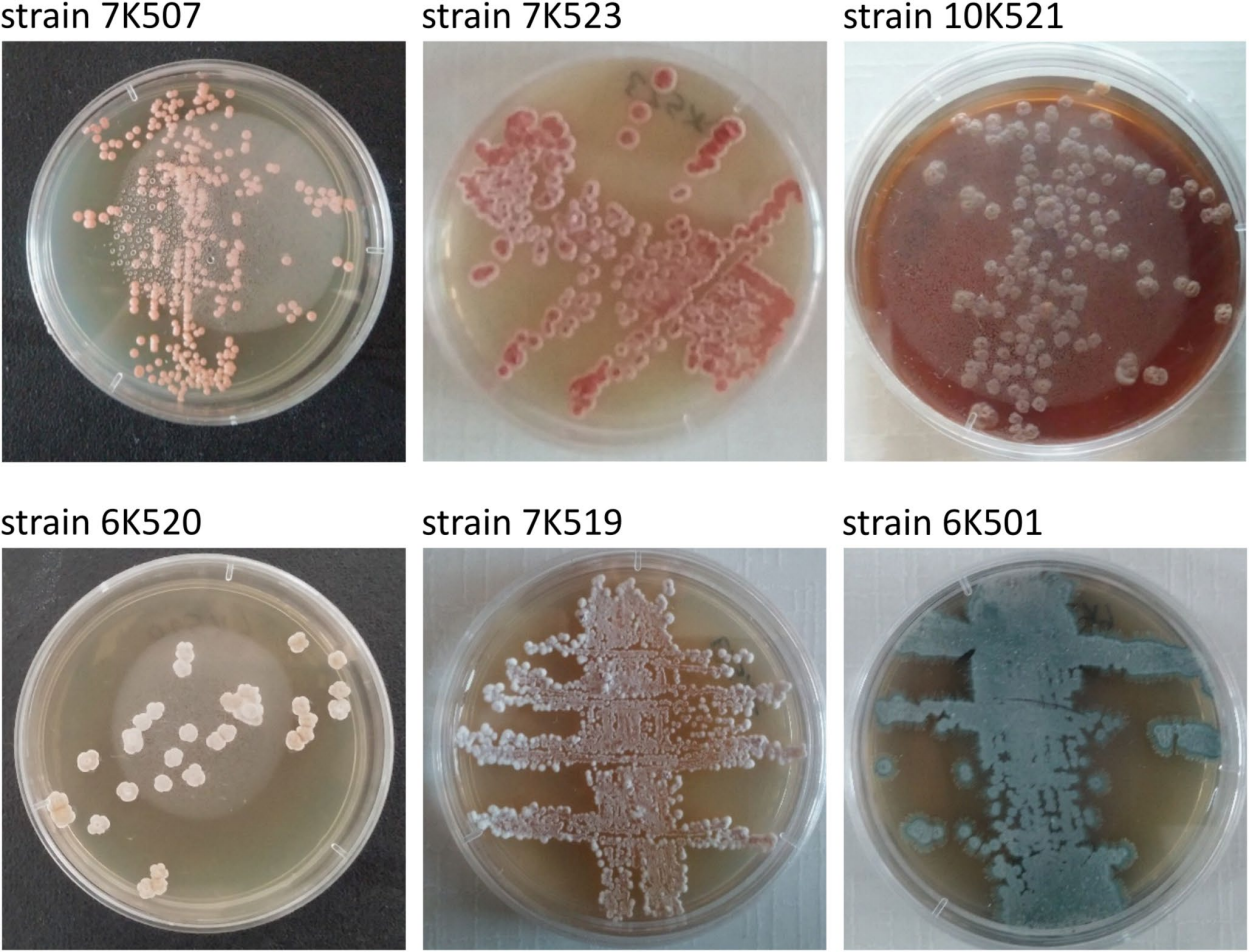
Representative filamentous actinomycetes growing on yeast extract-malt extract (ISP 2) agar plates showing colonies bearing pigmented spores on aerial mycelia after 14 days growth at 28 °C

The most pronounced range of colony types were from the samples collected from locations 5 and 6 (Fig. 4). The representative isolates from the sampling sites were assigned to 88 multiple-membered (2 to 13 isolates) and 35 single-membered colour-groups based on pigmentational properties recorded from the oatmeal agar plates (Table S2). Colony morphologies of a few selected isolates grown on ISP 2 agar, amycelial isolates were distributed to colour-groups based on colony colour (Fig. 5).

**Table 4:** Total number of actinomycetes (10^3^ Cfu/g dry weight soil) growing on isolation media inoculated with serial dilutions prepared from environmental samples collected from the sampling sites following incubation at 28 °C for four weeks

Sample Sites	1	2	3	4	5	6	Total counts
**Media**							
SM1 agar	-	1.0	1.5	-	3.5	0.5	6.5
SM2 agar	1.5	-	1.5	-	3.0	2.5	8.5
SM3 agar	1.5	1.5	0.5	0.5	1.5	6.5	12
Marine agar	15.5	12.8	10.6	7.9	22.4	75.1	144.3
Humic acid-vitamin agar	178.2	78.5	145.5	55.2	217.3	128.5	803.2
M1 agar	4.8	1.6	4.6	2.9	25.4	29.6	68.9
Non-sporulating agar	8.5	2.3	4.1	2.6	25.5	4.6	47.6
R2A agar	4.8	5.2	7.1	1.5	5.0	45.8	69.4
Starch-casein agar	28.8	36.5	34.6	42.5	52.2	42.0	236.6
Raffinose-histidine agar	7.5	13.2	12.8	2.5	38.5	1.0	75.5
Tryptic soy agar	2.0	-	-	1.5	3.5	1.5	8.5
Oligotrophic agar	12.5	4.5	5.0	5.4	6.5	2.0	35.9
Minimal agar	17.5	17.8	20.5	2.0	3.0	-	60.8
Complex agar	1.0	4.5	5.5	1.2	1.5	0.5	14.2
Glycerol-asparagine agar	4.5	1.8	25.5	10.3	1.2	-	43.3
Chitin-vitamin agar	16.5	15.8	26.4	13.2	2.1	3.0	77
**Average counts**	19.0	12.3	19.1	9.3	25.8	21.4	106.9

-, Actinomycete colonies were not present on the isolation plates

### Taxonomic diversity of representatives of colour-groups

The 16 S rRNA gene sequence analyses showed that 259 out of the 270 isolates (96%) taken to represent the colour-groups belong to genera classified in the phylum *Actinomycetota* (Table 5), the remainder were assigned to eleven non-actinomycete taxa (Table S2).

**Table 5:** Assignment of representative Karakum desert isolates to genera, families and orders (*n* = 259 isolates) *

Order	Family	Genus	Isolate
*Corynebacteriales*	*Nocardiaceae*	*Nocardia*	6K504, 6K505, 6K519, 7K517, 7K520, 7K528, KC209, KC320, KC910, KC916
*Jiangellales*	*Jiangellaceae*	*Jiangella*	1K503, 5K116, *5K138^T^, *8K307^T^, *KC603^T^
*Microbacteriales*	*Microbacteriaceae*	*Agromyces*	4K403B
		*Microbacterium*	*5K110
*Micromonosporales*	*Micromonosporaceae*	*Micromonospora*	4K205, 5K118, 5K221, 5K409, 5K544, 7K104, 7K504, 7K530, 8K103, 8K503, 10K527, 12K108, 12K402, 13K111, 13K203, *13K206^T^, 13K208, 15K101, 15K103, 15K303, *15K316, 16K103, 16K305, 16K405, KC202, KC204, KC205, *KC207, KC208, *KC213, KC215, KC605, *KC606, KC707, KC708, *KC721, *KC723
		*Plantactinospora*	5K208, KC601, KC705, KC725, KC728, KC729
*Propionibacteriales*	*Kribbellaceae*	*Kribbella*	7K109, *16K104^T^
	*Nocardioidaceae*	*Nocardioides*	KC13^T^
*Pseudonocardiales*	*Pseudonocardiaceae*	*Pseudonocardia*	5K133, 5K313, 5K418, KC301, KC304, KC311, KC316, KC323, KC325, KC502, KC504, KC510
		*Saccharomonospora*	6K501
		*Saccharopolyspora*	4K102, 5K506, *5K548^T^, 6K514, *7K502^T^, *16K309^T^, *16K404^T^, KC101, KC103, KC408
*Streptomycetales*	*Streptomycetaceae*	*Streptomyces*	3K201, 3K203, 3K401, 4K203, 4K301, 4K405, 5K101, 5K103, 5K109, 5K114, 5K122, 5K125, 5K131, 5K132, 5K135, 5K209, 5K312, 5K316, 5K417, 5K505, 5K520, 5K524, 5K526, 5K531, 5K532, 5K551, 6K103, 6K201, 6K502, 6K525, 7K111, 7K201, 7K301, 7K304, 7K305, 7K501, 7K513, 7K522, 7K529, 8K102, 8K205, 8K206, 8K301, 8K308, *8K508, 10K101, 10K102, 10K208, 10K209, 10K210, 10K212, 10K305, 10K307, 10K402, 10K507, 10K514, 10K516, 11K101, 11K402, 11K502, 12K107, 12K109, 12K203, 12K302, 12K506, 13K103, 13K105, 13K109, 13K204, *13K301^T^, 13K403, 14K201, 14K203, 14K501, 15K302, 15K408, 16K102, 16K105, 16K107, 16K205, 16K207, 16K210, 16K211, 16K303, 16K306, 16K311, 16K401, 16K501, KC11, KC12, KC15, KC18, KC319, KC340, KC403, KC407, KC410, KC416, KC421, KC426, KC501, KC506, KC903, KC907, KC918, KC930
*Streptosporangiales*	*Nocardiopsaceae*	*Nocardiopsis*	3K402, 5K222, 5K521, 10K510, 15K402, 16K403
	*Streptosporangiaceae*	*Nonomuraea*	1K501, 4K104, 4K202, 4K403A, 5K134, 5K320, 5K322, *6K102^T^, 7K110, 7K519, 7K523, 7K532, 8K306, 15K203, 15K301, *KC201^T^, KC306, *KC310^T^, KC327, KC332, *KC333^T^, *KC401, KC406, KC507, KC509, KC512, KC518, KC706, *KC712^T^, KC713, KC727, KC730, KC921, KC924, KC929
		*Spongiactinospora*	*13K107^T^
	*Thermomonosporaceae*	*Actinocorallia*	5K502, 5K516, 5K550
		*Actinomadura*	5K515, 5K555, 6K515, *6K520, 6K521, 6K523, 7K401, *7K507, 7K515, 7K525, 7K527, 7K533, *7K534, 7K537, 10K502, 10K504, 10K511, 10K521, *KC06, *KC216, *KC345, KC608

Based on the classification of Salam et al. (2020) *Isolates included in the phylogenomic analyses

The following codes show the source of the isolates and the selective medium on which they were cultivated (a) The numbers before the “K” code refer to selective isolation medium (i.e. 1–16 as shown in Table 4) and after the “K” code they refer to the environmental sample (i.e. 1–5, as shown in Fig. 1) (first digit) and strain numbers (last two digits), as exemplified by 13K206, isolate 6 from site 2 isolated on minimal medium agar; (b) “KC” refers to isolates recovered from sampling site 6, and the following numbers indicate the isolation medium (1–16 as before) and the remaining digits refer to the number of the isolate from that source, as illustrated by KC712, isolate 12 from non-sporulating agar and; (c) if only two digits appear after “KC” (KC06, KC13, etc.), this indicates a pilot isolation study from the Karakum Desert and that the strains isolated from starch-casein agar from sampling site 6

**Fig. 6:**
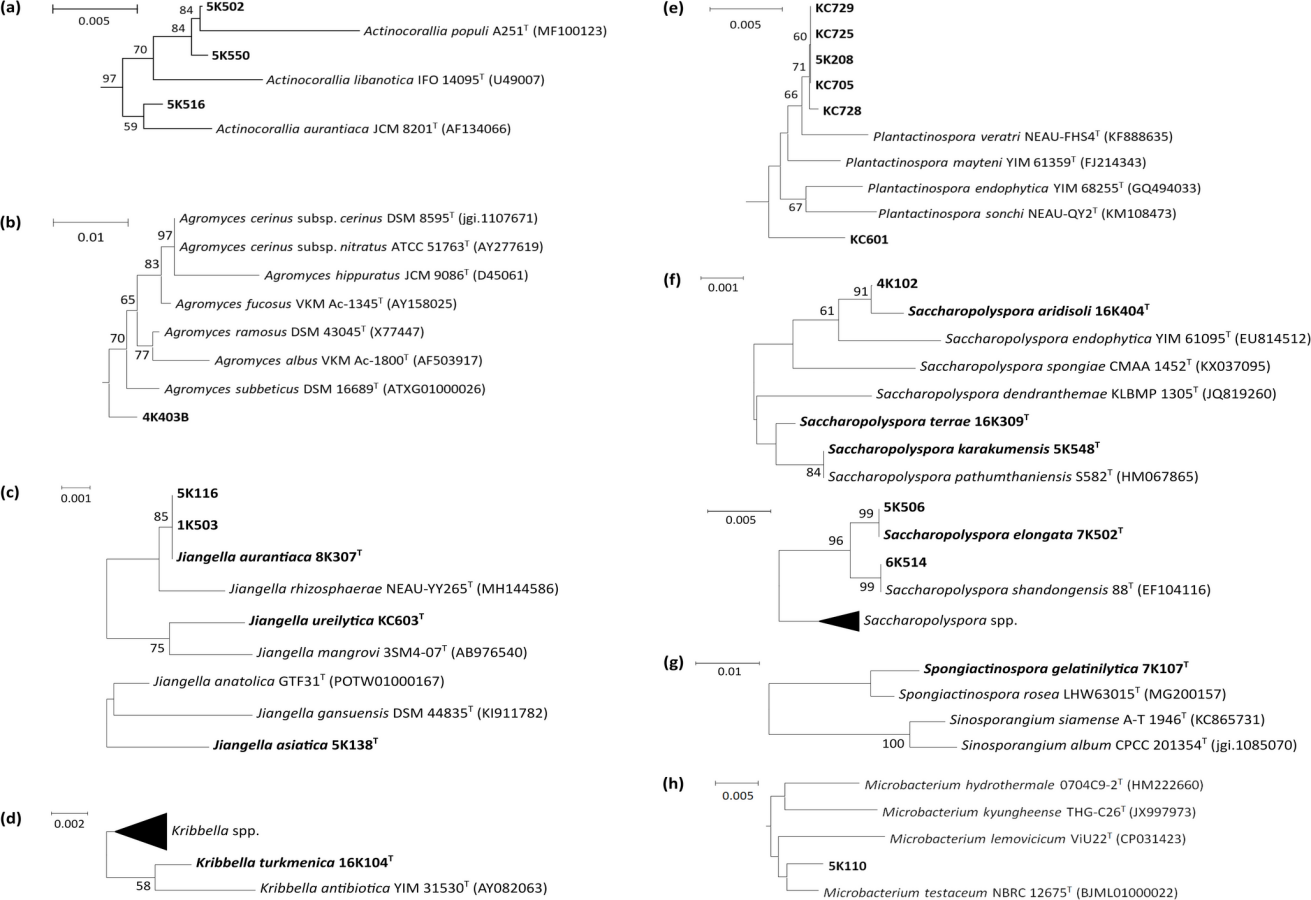
Abbreviated 16 S rRNA gene neighbor-joining trees showing relationships between isolates and their closest phylogenetic type strains in the genera *Actinocorallia* (**a**), *Agromyces* (**b**), *Jiangella* (**c**), *Kribbella* (**d**), *Plantactinospora* (**e**), *Saccharopolyspora* (**f**), *Spongiactinospora* (**g**), and *Microbacterium* (**h**). Scale bars are given in each of the figures. The phylogenetic trees were constructed based on 16 S rRNA gene sequence alignments of 1,371 bp (**a**), 1,395 bp (**b**), 1,440 bp (**c**), 1,334 bp (**d**), 1,370 bp (**e**), 1,270 bp (**f**), 1,316 bp (**g**), and 1,390 bp (**h**)

**Fig. 7:**
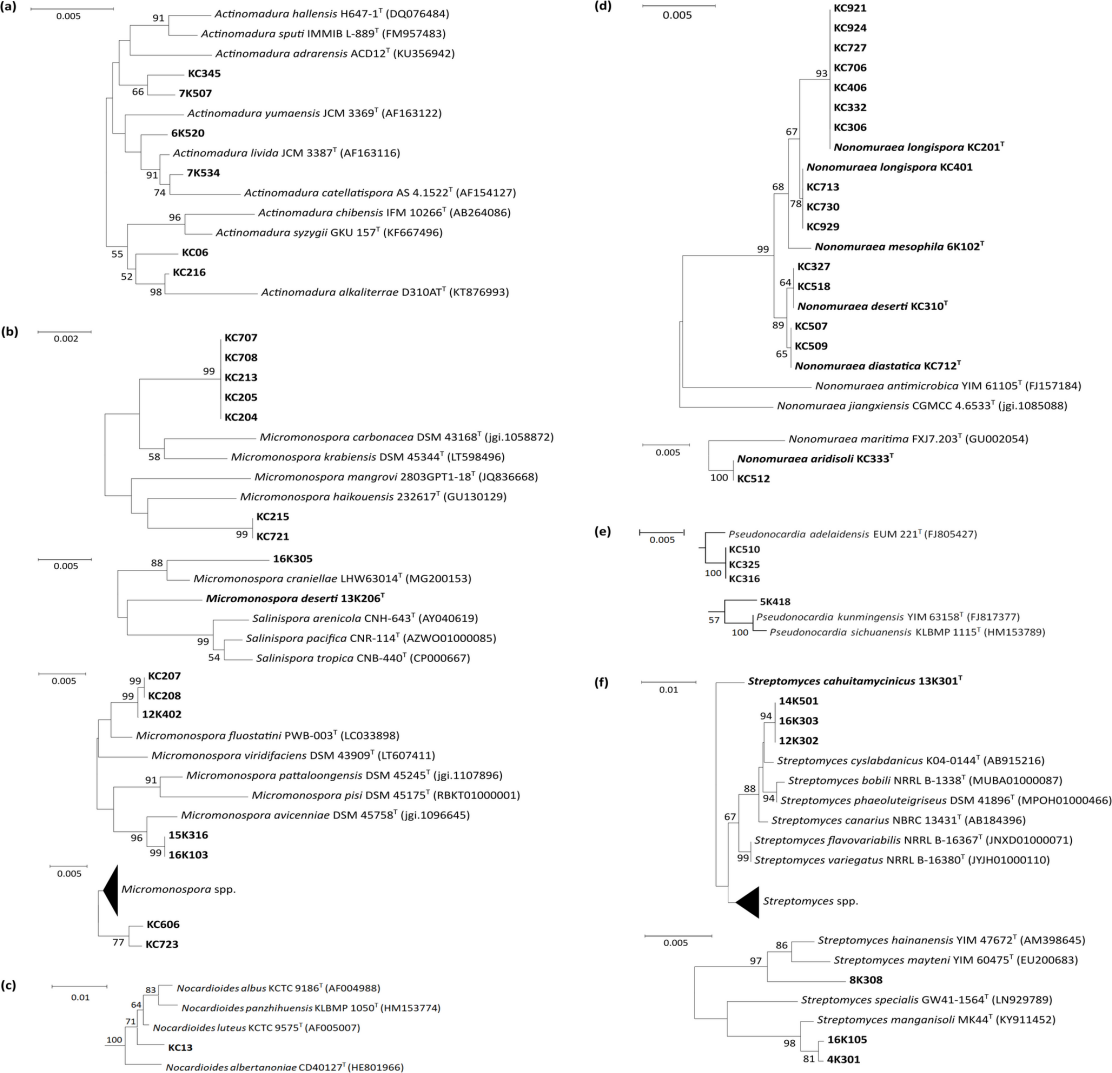
Abbreviated 16 S rRNA gene neighbor-joining trees showing relationships between the isolates and their closest type strains in the genera *Actinomadura* (**a**), *Micromonospora* (**b**), *Nocardioides* (**c**), *Nonomuraea* (**d**), *Pseudonocardia* (**e**), and *Streptomyces* (**f**). Scale bars are given in each of the figures. The phylogenetic trees were constructed based on 16 S rRNA gene sequence alignments of 1,287 bp (**a**), 1,380 bp (**b**), 1,332 bp (**c**), 1,305 bp (**d**), 1,385 bp (**e**), and 1,301 bp (**f**)

Sixty-eight of the actinomycete isolates are considered to represent putative novel species, as their 16 S rRNA gene sequence similarities to the closest known type strains fall below the 99.3% threshold. This threshold was conservatively adopted in this study to indicate high novelty potential, particularly in genera such as *Actinomadura*, *Micromonospora*, *Nocardioides*, *Nonomuraea*, and *Streptomyces* (with 7, 9, 1, 27, and 15 isolates, respectively), which are widely distributed in desert habitats. In addition, there are putative novel species, two isolates from the genus *Actinocorallia* and one isolate each from the genera *Agromyces*, *Kribbella*, *Microbacterium*, *Plantactinospora* and *Spongiactinospora* that have rarely been isolated from desert biomes (Table S3). The relationships between putatively novel isolates and their closest phylogenetic relatives are shown (Figs. 6 and 7). 36 of the isolates (14%) belong to taxa rarely, if ever, isolated from arid desert habitats, namely the genera *Actinocorallia* (3 isolates), *Agromyces* (1), *Jiangella* (5), *Kribbella* (2), *Microbacterium* (1), *Nocardiopsis* (6), *Plantactinospora* (6), *Saccharomonospora* (1), *Saccharopolyspora* (10) and *Spongiactinospora* (1) (Figures S1-S10). Eleven of these isolates (31%) were separated from the type strains of their closest phylogenetic neighbours by relatively long branches and 16 S rRNA gene sequence similarities at or below the 99.3% threshold (Table S3). Eleven of the isolates shared identical or almost identical 16 S rRNA gene sequence similarities with the type strains of validly named species, as shown by relationships between isolates KC101, KC103 and KC408 and *Saccharopolyspora erythraea*, which produces the clinically important macrolide antibiotic, erythromycin A (Cortes et al. 1990). The remaining 223 isolates (86%) representing the colour-groups were assigned to genera known to be common in desert biomes, including *Actinomadura* (22 isolates), *Micromonospora* (37), *Nocardia* (10), *Nocardioides* (1), *Nonomuraea* (35), *Pseudonocardia* (12) and *Streptomyces* (106) (Figures S11 to S16). Fifty-seven of these isolates (26%) were considered to belong to putative novel species using the 99.3% cut-off point. On the other hand, fifty-six isolates shared identical or almost-identical 16 S rRNA gene sequence similarities with the type strains of their nearest phylogenetic neighbours. Identical sequences, for instance, were found between isolates 5K515, 6K523, and 7K401 and the type strain of *Actinomadura geliboluensis*.

### Classification of isolates assigned to colour-groups

**Fig. 8:**
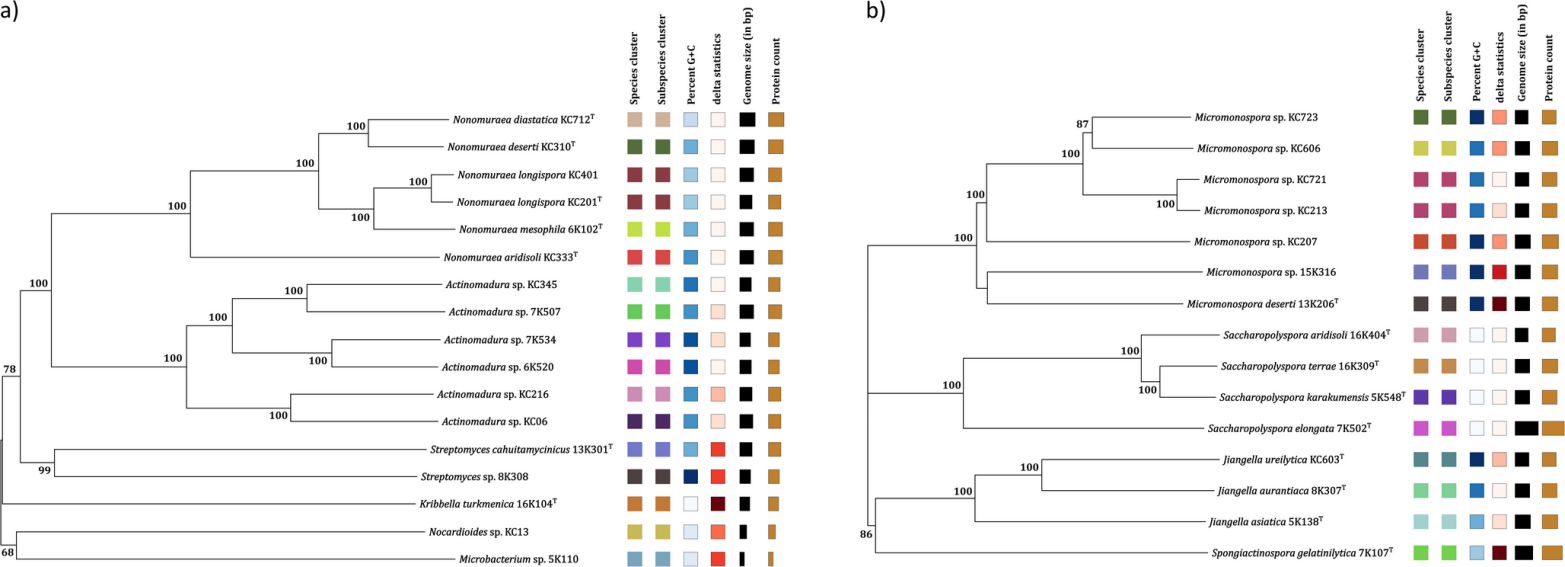
Phylogenomic tree showing relationships between isolates belonging to the genera (**a**) *Actinomadura*, *Kribbella*, *Microbacterium*, *Nocardioides*, *Nonomuraea* and *Streptomyces*, and (**b**) *Spongiactinospora*, *Jiangella*, *Micromonospora* and *Saccharopolyspora*. The numbers above branches are GBDP pseudo-bootstrap support values > 60% from 100 replications, with average branch supports of 96.1% and 94.2%, respectively. Trees were inferred with FastME 2.1.6.1 (Lefort et al. 2015) from GBDP distances calculated from genome sequences. The branch lengths are scaled in terms of the GBDP distance formula d_5_. The trees were rooted at the midpoint (Farris 1972). δ statistics were 0.103 and 0.12, respectively

Most isolates assigned to colour-groups can be classified into genera and species given the distribution of representative marker strains included in the 16 S rRNA gene sequence analyses (Idris 2016; Kusuma 2020; Świecimska et al. 2023). In the present study, for instance, colour-group 88 encompasses all of the *Actinocorallia* strains whereas colour-groups 57 and 86 can be provisionally equated with two validly named species, *A. geliboluensis* and *Actinomadura cremea* (Table S4). Similarly, colour-groups 1, 20, 25, 72 and 85 can be equated with *Micromonospora zamorensis*, *Micromonospora saelicesensis*, *Micromonospora avicenniae*, *Micromonospora citrea* and *Micromonospora fluostatini*, and colour-groups 40, 42 and 76 with *Nocardiopsis trehalosi*, *Nocardiopsis umidischolae* and *Nocardiopsis dassonvillei*. It is also encouraging that the type strains of *Kribbella turkmenica*, *Jiangella ureilytica*, *Nonomuraea diastatica*, *Nonomuraea mesophila*, *Saccharopolyspora terrae* and *Spongiactinospora gelatinilytica* which formed distinct lineages in the phylogenomic tree (Fig. 8) were recovered as single-membered colour-groups (Table S3).

By far the most extensive taxonomic diversity was shown by isolates assigned to the genus *Streptomyces* (Table S4), including ones representing presumptive novel species (Table S2). Indeed, 41 out of the 88 multi-membered colour-groups (47%) were composed of streptomycetes, as exemplified by colour-group 31 which contains isolates shown to be phylogenetically close to the type strains of *Streptomyces griseoincarnatus*, *Streptomyces leeuwenhoekii* and *Streptomyces thinghirensis*. Another relatively large taxon, colour-group 50, consists of four isolates with identical 16 S rRNA gene sequences to the type strains of *Streptomyces calvus*, an isolate most closely related to *Streptomyces albogriseolus* and five similarly related to *Streptomyces atrovirens*.

A case in point is colour-group 23 which encompasses 10 isolates shown to be most closely related to the type strains of *Micromonospora costi*, *Micromonospora halophytica*, *Micromonospora inositola* and *Micromonospora palomenae*; seven of these isolates showed sequence similarities with their closest phylogenomic neighbours below the 99.3% threshold used in this study to delineate potentially new actinomycete species.

Ten of the remaining colour-groups (11.4%) encompassed isolates assigned to the genus *Nonomuraea*. These taxa included colour-group 67 which contains 5 isolates, including *Nonomuraea deserti* KC310^T^, that are most closely related to the type strains of *Nonomuraea candida*. Similarly, colour-group 82 consists of nine isolates, including *Nonomuraea longispora* KC201^T^, which also showed sequence similarities below the 99.3% threshold with the type strains of *Nonomuraea salmonea*. In addition, isolate 1K501 (colour group 44) and isolates 5K134 and 5K322 (colour-group 70) can be considered to be presumptive novel *Nonomuraea* species given their low sequence similarities (98.82 and 98.75%, respectively) with the type strains of *Nonomuraea ceibae* and *Nonomuraea maheshkhaliensis*, respectively.

In terms of colour-group numbers, less taxonomic diversity was found amongst isolates distributed to the remaining genera (Table S4). The number of colour-groups ranged from one with respect to isolates assigned to the genera *Actinomadura*, *Agromyces*, *Kribbella* and *Saccharomonospora* to five in the case of *Actinomadura*, as illustrated by colour-group 47 which contains isolates KC345 and 6K520 that are most closely related to the type strains of *Actinomadura bangladeshensis* and *Actinomadura livida*, respectively. Only three of the colour-groups included isolates belonging to more than one genus, notably colour-group 4, which included five isolates that shared high sequence similarities (99.44 to 99.51%) with *Plantactinospora veratri* NEAU-FHS4^T^, isolate 5K110, a presumptive novel *Microbacterium* species, and *Micromonospora deserti* 13K206^T^, which is most closely related (98.54% sequence similarity) to *Micromonospora nigra* DSM 43,818^T^.

### Antimicrobial activity of selected isolates

The results obtained when rare and presumptive novel isolates were screened against the panel of bacterial and fungal strains (Table 6). Many of the isolates inhibited the growth of the *P. aeruginosa* (44%), *C. albicans* (26%), and *S. aureus* (25%) strains. In contrast, few isolates showed activity against the *B. subtilis*, *E. faecalis* and *E. coli* strains. It is particularly interesting that most of the *Actinomadura* isolates, several of the *Micromonospora*, *Nonomuraea* and *Streptomyces* strains, *J. aurantiaca* 8K307^T^, *J. ureilytica* KC603^T^, *Sps. aridisoli* 16K404^T^, *Sps. elongata* 7K502^T^, *Sps. karakumensis* 5K548^T^ and *Sps. terrae* 16K307^T^ inhibited the growth of *P. aeruginosa* ATCC 27,853, as did *K. turkmeniaca* 16K104^T^ and *Microbacterium* 5K110. Five out of the 8 isolates that inhibited the *E. faecalis* strain belonged to the genus *Nonomuraea*. Among the isolates, only two *Streptomyces* isolates, 13K109 and KC501, inhibited the growth of *A. brasiliensis* ATCC 16,404. None of the isolates showed activity against *K. quasipneumoniae* ATCC 700,603. Antimicrobial activity was not shown by isolates assigned to the genera *Nocardioides*, *Plantactinospora*, *Pseudonocardia* or *Spongiactinospora*. Overall, 32 out of the 57 isolates (56%) inhibited (≥ 10 mm) the growth of one or more of the reference strains.

**Table 6:** Inhibition zones (mm) shown by representative isolates from the Karakum desert in the agar diffusion assays after incubation for 24–48 h at optimal temperatures. In total, 57 isolates were analysed for their antimicrobial activity. Inhibition zones ≥ 10 mm were considered positive for antimicrobial activity. 1, *Aspergillus Brasiliensis* ATCC 16,404; *2*, *Bacillus subtilis* ATCC 6633; 3, *Candida albicans* ATCC 10,231; 4, *Enterococcus faecalis* ATCC 29,212; 5, *Escherichia coli* ATCC 25,922; 6, *Pseudomonas aeruginosa* ATCC 27,853; 7, *Staphylococcus aureus* ATCC 25,923

Strain	1	2	3	4	5	6	7
*Actinomadura*							
5K555	-	-	9	-	-	18	55
^**#**^6K520	-	-	-	-	-	12	-
^**#**^7K507	-	-	6	-	-	5	-
7K515, 7K533, ^**#**^7K534	-	-	-	-	-	-	-
10K521	-	18		14	8	9	-
^**#**^KC06	-	20	7			6	-
^**#**^KC216	-	-	5	-	-	14	-
^**#**^KC345	-	-		-	-	10	-
*Jiangella*							
*^,**#**^5K138^T^	-	-	-	-	-	-	-
*^,**#**^8K307^T^	-	-	-	-	-	14	-
*^,**#**^KC603^T^	-	-	6	-	-	7	-
*Kribbella*							
*^,**#**^16K104^T^	-	-	12	-	-	12	20
*Microbacterium*							
*^,**#**^5K110	-	-	-	-	-	9	-
*Micromonospora*							
^**#**^13K206^T^	-	-	-	-	20	-	**-**
^**#**^15K316	-	-	-	-	-	6	-
^**#**^KC207	-	-	-	-	-	-	20
^**#**^KC213	-	-	-	-	-	12	-
^**#**^KC606	-	-	-	-	-	7	-
^**#**^KC721, ^**#**^KC723	-	-	-	-	-	-	-
*Nocardioides*							
^**#**^KC13^T^	-	-	-	-	-	-	-
*Nonomuraea*							
1K501	-	-	-	-	-	-	-
4K202	-	-	-	-	-	-	7
5K322	-	-	10	-	-	-	-
^**#**^6K102^T^	-	-	-	-	-	-	-
7K110	-	-	8	-	-	-	-
7K523	-	-	7	-	-	-	13
8K306	-	-	-	-	-	7	-
15K203	-	-	8	-	-	11	-
^**#**^KC201^T^	-	-	-	23	-	-	**-**
^**#**^KC310^T^	-	-	-	22	-	-	**-**
^**#**^KC333^T^	-	15	-	23	-	7	25
^**#**^KC401	-	-	-	18	-	-	**-**
^**#**^KC712^T^	-	-	-	24	-	-	**-**
*Plantactinospora*							
*KC601, *KC728	-	-	-	-	-	-	-
*Pseudonocardia*							
5K418, KC304	-	-	-	-	-	-	-
*Saccharopolyspora*							
*^,**#**^5K548^T^	-	-	-	-	-	16	-
*^,**#**^7K502^T^	-	-	-	58	-	15	53
*^,**#**^16K309^T^	-	-	12	-	-	12	40
*^,**#**^16K404^T^	-	-	-	-	-	12	-
*Spongiactinospora*							
*^,**#**^7K107^T^	-	-	-	-	-	-	-
*Streptomyces*							
3K401	-	-	-	-	-	-	-
5K209	-	-	-	-	-	7	12
6K502	-	-	14	-	-	25	-
^**#**^8K308	-	-	-	28	-	-	30
10K307	-	-	-	-	-	-	-
11K402	-	-	60	-	-	-	58
13K105	-	-	12	-	-	-	52
13K109	28	-	-	-	-	12	-
^**#**^13K301^T^	-	-	-	-	-	-	47
16K105	-	35	-	-	-	-	-
16K401	-	-	-	-	-	-	-
KC501	18	-	13	-	-	18	48

*, representative of rare genera. ^**#**^, isolates selected for whole-genome sequencing, the remaining strains represent putatively novel species

### Genomic features of selected isolates

The genome features of the 32 isolates used to generate the phylogenomic tree are shown (Table 7). The sizes of their draft genomes of the strains ranged from 3.71 Mbp for *Microbacterium* 5K110 to 10.86 Mbp for *Non. diastatica* KC712^T^. Many of the isolates can be considered to be extremely gifted *sensu* Baltz (2017) as they have large genomes (≥ 8 Mbp) and hence can be expected to be rich in BGCs encoding for known and novel specialized metabolites. Similarly, the remaining isolates, apart from the *Microbacterium* strain and *Sps. elongata* 7K502^T^, are moderately gifted with genome sizes ranging from 5.30 to 7.76 Mbp in *Noc. turkmenicus* KC13^T^ and *Streptomyces* 8K308, respectively. The digital (d) G + C contents of the isolates ranged from 69.4% in *K. turkmenica* 16K104^T^ to 72.5% in *J. ureilytica* KC603^T^ and *Streptomyces* 8K308, respectively. The lowest number of protein coding genes, 3,466, was recorded for the *Kribbella* strain and the highest number, 9,740 for *Non. diastatica* KC712^T^. The number of rRNA and tRNA operons found in the genomes of the isolates varied from 3 to 17 and from 44 to 79.

The genomes sizes of the *Actinomadura* isolates (8.09–9.80 Mbp) are well within the range (6.17–10.26 Mbp) reported for type strains of *Actinomadura* species. Similar deductions can be drawn from analyses of the *Kribbella*, *Nonomuraea*, *Spongiactinospora*, *Streptomyces*, *Jiangella* and *Micromonospora* isolates. In contrast, the genome sizes of the type strains of *Saccharopolyspora*, namely 6.29–9.02 Mbp (Nouioui et al. 2019), were exceeded by that of *Sps. elongata* 7K502^T^ with a genome size of 10.30 Mbp. It is also interesting that *Actinomadura* isolates 6K520 and 7K534 have similar genomic features as they formed a distinct lineage in the phylogenomic tree (Fig. 8). The genome of *Noc. turkmenicus* KC13^T^ (5.3 Mbp) is larger than expected for a *Nocardioides* strain, but its dDNA G + C content is within the normal range for members of the genus *Nocardioides*. Similarly, *Microbacterium* isolate 5K110, like other *Microbacterium* strains, has a relatively large genome (3.7 Mbp) and a high dDNA G + C content (70.0). These values place the isolate at or near the top of the ranges shown by *Microbacterium* type strains.

**Table 7:** Genome features of presumptively novel actinomycetes. 32 isolates underwent whole-genome sequencing and genomic analyses

Genera and strains	Genome		Digital DNA G + C content (mol%)	Contigs	Genes	Protein coding genes	rRNA operons	tRNA operons	GenBank accession numbers
	range (in bp)	coverage							
*Actinomadura*									
6K520	8,190,264	159x	71.9	445	7,580	7,290	11	61	SMLC00000000
7K507	9,799,144	45x	71.3	563	9,053	8,698	14	63	SMKK00000000
7K534	8,085,395	172x	72.1	565	7,539	7,244	14	61	SMKB00000000
KC06	9,380,427	74x	71.3	421	8,682	8,328	9	67	SMKT00000000
KC216	9,063,532	31x	71.3	667	8,576	8,215	11	79	SMJX00000000
KC345	8,265,010	34x	71.8	600	7,969	7,618	7	63	SMKH00000000
*Jiangella*									
5K138^T^	7,034,063	88x	71.0	134	6,407	6,207	3	45	SMKZ00000000
8K307^T^	6,620,775	152x	71.8	102	6,010	5,816	5	44	SMLB00000000
KC603^T^	6,345,964	105x	72.5	168	5,834	5,632	5	45	SMKL00000000
*Kribbella*									
16K104^T^	7,447,142	20x	69.4	351	7,165	6,877	3	45	SMKR00000000
*Microbacterium*									
5K110	3,713,488	38x	70.0	179	3,593	3,466	4	48	VBUM00000000
*Micromonospora*									
13K206^T^	6,720,287	30x	72.4	615	6,415	5,981	6	49	POUB00000000
15K316	6,957,401	138x	72.4	455	6,457	6,147	5	59	SMKG00000000
KC207	7,188,146	31x	72.2	637	6,588	6,015	3	50	SMKJ00000000
KC213	6,426,733	65x	71.6	363	6,131	5,772	7	53	SMKF00000000
KC606	6,658,798	43x	71.7	429	6,308	5,833	7	48	SMKN00000000
KC721	6,461,179	118x	71.6	441	6,161	5,780	7	79	SMJY00000000
KC723	6,107,068	84x	72.2	292	5,740	5,350	7	66	SMKD00000000
*Nocardioides*									
KC13^T^	5,300,162	63x	69.7	47	5,106	4,962	6	47	JAALAA000000000
*Nonomuraea*									
6K102^T^	10,138,478	97x	70.8	427	9,682	9,217	12	62	SMLD00000000
KC201^T^	9,164,341	47x	70.6	465	8,789	8,281	12	66	SMJZ00000000
KC310^T^	10,694,046	144x	70.9	470	10,145	9,541	9	70	SMKO00000000
KC333^T^	9,868,672	30x	71.3	729	9,771	9,279	17	65	POUD00000000
KC401	9,792,882	73x	70.6	494	9,422	8,943	9	69	VBUN00000000
KC712^T^	10,861,993	70x	70.3	488	10,459	9,740	5	66	SMKP00000000
*Saccharopolyspora*									
5K548^T^	6,670,713	76x	69.7	137	6,234	5,983	5	50	SMLA00000000
7K502^T^	10,298,286	250x	69.7	277	9,326	8,751	11	51	SMKW00000000
16K309^T^	6,653,094	112x	69.7	167	6,243	6,004	7	51	SMKS00000000
16K404^T^	6,008,184	163x	69.6	83	5,389	5,147	8	51	SMKV00000000
*Spongiactinospora*									
7K107^T^	8,049,107	30x	70.8	957	8,033	7,513	8	61	POUA00000000
*Streptomyces*									
8K308	7,755,217	89x	72.5	703	7,341	6,676	9	66	SMKC00000000
13K301^T^	9,035,421	30x	70.8	1024	8,880	8,138	15	69	POUC00000000

### Phylogenomic relationships of taxonomically diverse isolates

The assignment of the selected isolates to well supported clades corresponding to the genera *Actinomadura*, *Jiangella*, *Micromonospora*, *Nonomuraea*, *Saccharopolyspora* and *Streptomyces* in the phylogenomic tree (Fig. 8a and b) confirm the generic relationships found in the corresponding 16 S rRNA gene trees (Figs. 6 and 7). It is also evident that several of the isolates belong to well supported lineages which correspond to ones found in the phylogenetic trees, as exemplified by the assignment of *Actinomadura* isolates KC345 and 7K507, 6K520 and KC06 and KC216 to distinct, albeit related subclades (Fig. 8). The phylogenomic analysis also highlights presumptive novel isolates, including *Noc. turkmenicus* KC13^T^ and *Microbacterium* 5K110, as well as ones which correspond to known lineages, as exemplified by *J. asiatica* 5K318^T^, *Non. deserti* KC310^T^ and *Sps*. *elongata* 7K502^T^.

### Biosynthetic gene clusters

Several of the BGCs detected in the genomes of the *Actinomadura* isolates showed more than 50% gene identity with known bioclusters and were unique to these strains. For example, isolates KC06 and 7K507 harbored bioclusters partially similar to the macrocyclic lactams ML-449 (62% gene identity) (Jørgensen et al. 2010) and vicenistatin (55% gene identity) (Shindo et al. 1993), respectively. Similarly, the genome of *Actinomadura* 7K534 contained BGCs related to the biosynthesis of napyradiomycin (53% gene identity), an antioxidant with anti-inflammatory activity (Choi et al. 2020), and 7-prenylisatin (60% gene identity), an isatin-type antibiotic active against *Bacillus subtilis* (Liang and Wang 2019). Additionally, isolates KC06 and KC216 possessed BGCs similar to those associated with catenulipeptin (60% gene identity), a class III lanthipeptide (Wang and van der Donk 2012), and citrulassin D (80% and 100% gene identity, respectively), a lasso peptide (Tietz et al. 2017).

The genomes of the *Nonomuraea* isolates, including the type strains of *Non. deserti*, *Non. diastatica*, *Non. longispora*, and *Non. mesophila* (Saygin et al. 2020d), harbored biosynthetic gene clusters (BGCs) showing 100% gene identity with clusters responsible for the production of alkylresorcinol, a common phenolic lipid (Kozubek and Tyman 2005), and rhizomide A, a compound exhibiting weak antitumor activity (Wang et al. 2018). Furthermore, these genomes also contained BGCs known to encode indigoidine (80% gene identity), a natural pigment with antioxidant and antimicrobial properties (Yumusak et al. 2019), and GE2270 (80% gene identity), an antibiotic that inhibits protein synthesis in bacteria (Kettenring et al. 1991). In addition, all of the *Nonomuraea* isolates harbored BGCs associated with the production of chlortetracycline (5% gene identity), a broad-spectrum antibiotic active against a range of bacteria (Pulicharla et al. 2015).

As in earlier studies (Carro et al. 2018, 2019a), many of the BGCs detected in the genomes of the *Micromonospora* isolates were strain-specific. For instance, isolates KC207, KC213, KC606, KC721, KC723, 13K206, and 15K316 harbored bioclusters partially similar to known compounds. These included tirandamycin (40% gene identity), an antibiotic that inhibits transcription by disrupting the bacterial RNA polymerase (Reusser 1976); thiocoraline (5% gene identity), a depsipeptide exhibiting antitumoral activity (Sparidans et al. 1999); tallysomycin A (5% gene identity), a glycopeptide-derived antitumor antibiotic (Galm et al. 2011); colabomycin E (9% gene identity), an anti-inflammatory agent that inhibits caspase 1 and the synthesis of interleukins (Petříčková et al. 2014); hedamycin (12% gene identity), a polyketide antibiotic with anticancer properties (Hansen et al. 1995); azicemicin B (13% gene identity), an antimicrobial compound active against Gram-positive bacteria, including mycobacteria (Tsuchida et al. 1995); and crochelin A (11% gene identity), an unusual siderophore (Baars et al. 2018).

The genomes of the *Saccharopolyspora* strains were especially rich in BGCs predicted to encode a broad range of novel antibiotics. Specifically, *Sps. aridisoli* 16K404^T^, *Sps. elongata* 7K502^T^, *Sps. karakumensis* 5K548^T^, and *Sps. terrae* 16K309^T^ contained BGCs associated with the production of lankacidin C (13–20% gene identity), an antibiotic with antitumor activity (Ayoub et al. 2019), and the siderophore staphylobactin (25% gene identity). Additionally, *Sps. aridisoli* 16K404^T^, *Sps. karakumensis* 5K548^T^, and *Sps. terrae* 16K309^T^ harbored gene clusters predicted to encode a specialized metabolite similar to saquayamycin A, an aquayamycin antibiotic active against Gram-positive bacteria (Uchida et al. 1985). Furthermore, *Sps. karakumensis* 5K548^T^ and *Sps. terrae* 16K309^T^ possessed genes associated with the biosynthesis of pepticinnamin E (6% gene identity), a farnesyl-transferase inhibitor with potential applications in cancer and malaria treatment (Santa Maria et al. 2019), and brasilicardin A (30% gene identity), a diterpenoid antibiotic with immunosuppressive properties (Komaki et al. 1999).

The genome of *Spongiactinospora gelatinilytica* 7K107^T^ contained BGCs associated with a variety of bioactive compounds. These included amipurimycin (23% gene identity), a nucleoside antibiotic with antimicrobial activity (Harada and Kishi 1977; Iwasa et al. 1977); epoxomicin (25% gene identity), a proteasome inhibitor with in vivo anti-inflammatory properties (Meng et al. 1999); kedarcidin (5% gene identity), a chromoprotein antitumor antibiotic (Leet et al. 1992); and lobosamides A, B, and C (21% gene identity), polyene macrolactams active against the protozoan parasite *Trypanosoma brucei* (Schulze et al. 2015).

*Stm. cahuitamycinicus* 13K301^T^ and *Streptomyces* 8K308 harbored BGCs similar to those encoding the siderophore desferrioxamine E (50% and 83% gene identity, respectively) and ficellomycin (3% gene identity in both isolates), an aziridine antibiotic with potential therapeutic value (Liu et al. 2017; He et al. 2018). However, only isolate 8K308 contained BGCs associated with the synthesis of azalomycin F3a (26% gene identity), a macrocyclic polyketide (Zhai et al. 2020); herboxidiene (2% gene identity), a polyketide exhibiting herbicidal activity (Miller-Wideman et al. 1992); pentostatin (6% gene identity), a purine nucleoside antibiotic used to treat hematological cancers (Wu et al. 2017); and spiramycin (8% gene identity), a macrolide antibiotic used to treat *Toxoplasma gondii* infections during pregnancy (Montoya et al. 2021). In contrast, only *Stm. cahuitamycinicus* 13K301^T^ was found to harbor BGCs associated with the production of albaflavenone (100% gene identity), a sesquiterpene antibiotic exhibiting antibacterial activity (Zhao et al. 2008), and a bioactive metabolite similar to neocarzinostatin (41% gene identity), a chromoprotein antitumor antibiotic (Edo and Koide 1997).

Although all of the *Jiangella* strains were found to have the capacity to synthesize alkylresorcinol (100% gene identity), most of the BGCs were strain-specific. The genome of *J. aurantiaca* 8K307^T^ contained BGCs associated with the production of tetarimycin A (5% gene identity), a compound that inhibits methicillin-resistant *Staphylococcus aureus* strains (Kallifidas et al. 2012), whereas the genome of *J. asiatica* 5K138^T^ harbored BGCs related to the production of acumicin (4% gene identity), a cyclic depsipeptide with anti-mycobacterial activity (Hawkins et al. 2018). Finally, the genome of *J. ureilytica* KC603^T^ contained BGCs partially similar to those encoding primycin (5% gene identity), an antibiotic active against Gram-positive bacteria (Horváth et al. 1979), and LL-D49194α1 (7% gene identity), an antitumor antibiotic (Dong et al. 2019).

Four out of the ten BGCs detected in the genome of *K. turkmenica* 16K104^T^ were associated with the production of alkylresorcinol (100% gene identity), catenulipeptin (60% gene identity), and ficellomycin (9% gene identity), which were mentioned previously. Five out of the six BGCs identified in the genome of *Microbacterium* 5K110 were associated with the synthesis of known compounds, including carotenoid pigment (50% gene identity) and berninamycin A (26% gene identity), a cyclic thiopeptide antibiotic that inhibits protein synthesis in Gram-positive bacteria (Thompson et al. 1982). The remaining BGC is predicted to encode a novel metabolite. The genome of *Noc. turkmenicus* KC13^T^ contained six BGCs, three of which are novel. The remaining BGCs were associated with the production of desferrioxamine B (60% gene identity), a siderophore used to remove excess iron in patients with transfusion-related hemoglobin disorders (Codd et al. 2018); dynemicin A (5% gene identity), an anthraquinone antibiotic that inhibits Gram-positive bacteria (Konishi et al. 1989); and kijanimicin (4% gene identity), a macrolide antibiotic with antibacterial and antitumor properties (Waitz et al. 1981; Bradner et al. 1983).

### Plant growth promoting properties of selected isolates

The genomes of the isolates, notably those of the *Jiangella* strains, were rich in genes associated with phosphate metabolism and regulation (Table S5), as exemplified by genes *ppa*, *phoR*, *pstS* and *phoU* which express for solubilisation of inorganic phosphate (Lahti et al. 1988), a phosphate transport regulatory protein (Lamarche et al. 2008), a periplasmic protein involved in the synthesis of the phosphate ABC transporter (Willsky and Malamy 1980), and a transport protein regulating metabolism (Muda et al. 1992), respectively. In addition, all of the jiangellae have the genetic capacity to produce a range of phosphatases. In contrast, the genomes of none of the isolates contained genes associated with nitrogen fixation. Apart from the *Spongiactinospora* strain, the genomes of all of the isolates contain genes predicted to encode for indole − 3- glycerol phosphate synthase, the precursor of IAA in the tryptophan biosynthetic pathway in plants (Ouyang et al. 2000). They also have genes encoding for other components of this pathway, including aminase (*trpA* and *trpB*), anthranilate synthase (*trpE*) and anthranilate phosphoribosyl transferase (*trpD*) (Lambrecht and Downs 2013). The level of expression of IAA is influenced by the biosynthetic pathway, the location of the genes involved and their regulation (Duca et al. 2014). The genomes of most of the isolates were equipped with genes associated with the synthesis of chitinases, endoxylanase and ß-glucosidases. In contrast, the production of pectate lyase and 1,4-β-xylosidase was mainly restricted to the *Jiangella*, *Micromonospora*, *Nonomuraea* and *Streptomyces* strains. Genes predicted to encode for cutinases, which hydrolyse cutin ester bonds (Chen et al. 2013), were detected in the genomes of *Micromonospora* isolates KC213 and KC721, and *Stm*. *cahuitamycinicus* 13K301^T^. Cellulases (Juturu and Wu 2014), chitinases (Oyeleye and Normi 2018) and pectinases (Liu and Kokare 2017) are also noted for their industrial potential.

Genes predicted to encode for siderophores are discontinuously distributed across the genomes of the isolates (Table S6). In contrast, the genetic capacity of the isolates to produce ferric iron ABC transporters is restricted to the genera *Actinomadura*, *Jiangella*, *Kribbella* and *Micromonospora* whereas only *Micromonospora* isolates KC723 and 15K316, and *Stm. cahuitamycinicus* 13K301^T^ have more genes associated with the synthesis of desferrioxamine E (Smits and Duffy 2011).

### Genes of representative isolates associated with adaptation to desert habitats

The genomes of all of the isolates contained genes (e.g. *dnaK*, *grpE*, *hrcA*, *groL* and *groES*) linked with heat shock responses (Li et al. 2011) though only *Non. aridisoli* KC333^T^ contained genes *cspA* and *cspC*, which express for protein families that respond to cold shock (Jones and Inouye 1994). In contrast, without exception, the isolates had the capacity to produce cold shock protein SCO 4325. Other common genes include *ahpD*, which encodes for the synthesis of alkyl hydroperoxide reductase, an enzyme involved in compensatory interactions amongst hydrogen peroxide stress genes (Bsat et al. 1996) and in the protection of DNA against oxidative damage (Jacobson et al. 1989). Several isolates, especially those assigned to the genera *Kribbella*, *Nocardioides*, *Nonomuraea*, *Saccharopolyspora* and *Streptomyces*, have the ability to synthesize sarcosine oxidase, which is also associated with oxidative stress responses (Kappes et al. 1999; Mandon et al. 2003). Similarly, all of the strains, apart from the *Jiangella* and *Spongiactinospora* isolates, contained *katG*, a gene that encodes for catalase peroxidase, an enzyme which offers protection against reactive oxygen species (Normand et al. 2012). Many of the isolates, particularly the *Jiangella* and *Micromonospora* strains, have the potential to produce aquaporin Z, a protein that mediates the transport of water molecules across cell membranes (Johansson et al. 2000). Genes associated with carotenoid biosynthesis (e.g. *carB*, *crtB* and *crtL*) were found mainly in the genomes of the *Actinomadura* and *Saccharopolyspora* isolates (Table S7). Genes encoding for choline dehydrogenase (*chdH*) and choline kinase (*chkA*) were restricted mainly to the genomes of the *Nonomuraea* isolates whereas gene *betC*, which expresses for choline sulfatase, was detected in the genome of the *Jiangella* and *Micromonospora* strains, these genes are associated with oxidative stress and uptake of betaine and choline (Boncompagni et al. 1999, 2000; Nau-Wagner et al. 2012). The genomes of all of the isolates contained genes predicted to encode for copper resistance, as exemplified by *copC* and *copD*, which mediate resistance to copper by accumulating it in the periplasm and membrane proteins (Cha and Cooksey 1991). These genes were detected in all of the isolates, apart from *Actinomadura* KC06 and *J. asiatica* 5K138^T^ in the case of the former and *Spa. gelatinilytica* 7K107^T^ with respect to the latter. In contrast, the capacity to resist arsenic was restricted to *Microbacterium* 5K110, *Micromonospora* KC207 and 15K316, *Noc. turkmenicus* KC13^T^, *Non. diastatica* KC201^T^, *Non. longispora* KC201^T^, *Sps. elongata* 7K502^T^ and *Sps. terrae* 16K309^T^. Interestingly, resistance to chromium compounds was limited to the five *Nonomuraea* strains, *Microbacterium* 5K110 and *Micromonospora* KC207 and *Noc. turkmenicus* KC13^T^. The genomes of all isolates encode DNA gyrase subunits A and B, which are essential for DNA replication and supercoiling. Although these enzymes are not inherently involved in antibiotic resistance, mutations in their encoding genes (*gyr*A and *gyr*B) have been associated with resistance to fluoroquinolones (Ruiz 2003). Similarly, most of the isolates have the capacity to produce β-lactamases. In contrast, resistance to vancomycin is found to be rare among the isolates.

The genomes of all of the isolates contained genes associated with DNA repair (Table S8), as exemplified by the UvrABC excinuclease system which repairs DNA damage by cutting DNA strands on both sides of the damaged region (Sancar and Rupp 1983). Similarly, all of the isolates harboured genes predicted to express for ATP-dependent DNA ligases (*lig*C and *lig*D), DNA repair proteins (*rad*A and *rec*N), exodeoxyribonucleases (III, VII small and large subunits), endonucleases (III, IV and VIII), RecA protein and SOS-response repressor and protease (*lex*A). Three genes, *recF*, *recO* and *recR*, which are associated with DNA recombination and repair proteins, were detected in all the genomes, except *K. turkmeniaca* 16K104^T^. This isolate was one of the few strains with the capacity to produce exodeoxyribonuclease V alpha (*rec*D), beta (*rec*B) and gamma (*rec*C) chains, which are part of the bacterial RecBCD pathway (Pavankumar et al. 2010).

The genomes of all of the isolates contained *whiB*, a gene which encodes sporulation protein WhiB that is needed for the differentiation of aerial hyphae into mature spores in *Streptomyces coelicolor* (Molle et al. 2000). Gene *whiD*, which also contributes to spore differentiation, was detected in the genomes of the *Jiangella*, *Micromonospora*, *Saccharopolyspora* and *Streptomyces* strains. Several of the isolates contained genes (e.g. whiE-ks, whiE-clf, whiE VII) that express for spore pigments in *Streptomyces* strains (Swiercz and Elliot 2012), as illustrated by whiE VII, which is present in the genomes of all of the *Micromonospora* strains, most of the *Actinomadura* and *Nonomuraea* isolates, and in *Spa. gelatinilytica* 7K107^T^ and *Stm. cahuitamycinicus* 13K301^T^ (Table S9).

## Discussion

Since the assignment of filamentous actinomycetes, notably streptomycetes, to colour-groups provide an insight into the level of cultivable actinomycete diversity in natural habitats (Goodfellow et al. 2018; Świecimska et al. 2023), it can be concluded that the Karakum Desert habitats contain diverse communities of mainly filamentous actinomycetes. The extent of this diversity is similar to that found in corresponding studies on hyper-arid and extremely hyper-arid Atacama Desert soils (Okoro et al. 2009; Busarakam 2014; Idris 2016; Świecimska et al. 2023). The isolates obtained from this study were assigned to 17 genera, 11 families and 8 orders (Table 5). A similarly, broad range of taxa were found in comparable surveys of actinomycete communities in Atacama Desert soils (Busarakam 2014; Idris 2016; Goodfellow et al. 2018) though representatives of the families *Jiangellaceae* and *Microbacteriaceae* were not detected in these studies. In contrast, putative novel *Actinomadura*, *Kribbella*, *Nocardia*, *Nocardiopsis*, *Nonomuraea*, *Pseudonocardia* and *Streptomyces* strains were isolated from both Atacama and Karakum Desert soils. Streptomycetes isolated from these biomes and from the Kazakhstan Desert (Ziyat et al. 2019) proved to be the richest source of presumptive novel species. An interesting feature of the present study was the isolation of presumptive novel members of rare genera such as *Jiangella*, *Plantactinospora* and *Spongiactinospora*. Several of the selective media did not to support the growth of the target actinomycetes but did allow that of non-target taxa. The failure, for instance, to isolate *Amycolatopsis* strains on SM1, SM2 and SM3 agar media designed for this purpose (Tan et al. 2006) may be due to their absence in the Karakum Desert environmental samples whereas the growth of members of other taxa, such as *Actinocorallia*, *Actinomadura*, *Micromonospora*, *Nocardia*, *Pseudonocardia*, *Saccharopolyspora* and *Streptomyces*, can be attributed to the lack of competition on the isolation plates reflecting the low incidence of actinomycete propagules in the soil suspensions used to inoculate them. Similar “anomalous” results have been reported previously (Vickers et al. 1984; Busarakam 2014).

AntiSMASH predicts BGCs and potential natural products based on the percentages of genes from the closest known bioclusters which show BLAST hits with corresponding BGCs in the genomes under consideration. The number of bioclusters found in the genomes of the strains ranges from 4 in the genome of *Microbacterium* 5K110 to 50 in that of *Micromonospora* KC207. The genomes of all of the isolates contained bioclusters predicted to encode for core metabolites like ectoine, a protective molecule which enables bacteria to counter extreme conditions (Graf et al. 2008), arylpolyene-like compounds that are structurally and functionally similar to carotenoids (Schöner et al. 2016) and which show antimicrobial and antioxidant activity (Narsing Rao et al. 2017), geosmin, a volatile terpene responsible for earthy odours (Gerber and Lechevalier 1965) and melanin pigments which have antioxidant, antitumor and antimicrobial activity and protect microorganisms from UV-radiation (Vasanthabharathi and Jayalakshmi 2020). In contrast, most of the bioclusters predicted to encode for druggable molecules, including antibiotics, were discontinuously distributed across the genomes of the isolates. In general, the genomes of the selected isolates showed similar patterns of BGCs, notably ones predicted to encode for non-ribosomal peptide synthetases (NRPS), type I polyketide synthases (T1PKS), lanthipeptides and terpenes. Microorganisms which contain lanthipeptide gene clusters tend produce novel compounds, such as antibiotics, which inhibit the growth of multidrug-resistant *S. aureus* (Repka et al. 2017). The average number of NRPS bioclusters per genome was 4 out of 135 bioclusters, the corresponding numbers for the T1PKS and terpenes were 3 (112 bioclusters) and 4 (140 bioclusters), respectively. Many of the bioclusters were discontinuously distributed across the phylogenomic lineages with some shown to be taxon and strain specific, results that are in agreement with recent data on closely related *Streptomyces* species (Vicente et al. 2018; Park and Andam 2019; Martinet et al. 2020). Critically, 336 of the 861 BGCs (39%) did not show any similarity to known compounds while the number of bioclusters sharing more than 50% gene identity with known metabolites was low at 130 bioclusters (15%). *Actinomadura* and *Nonomuraea* strains are difficult to recognise on isolation plates and because of this they have rarely featured in bioprospecting campaigns even though it is apparent that they have the ability to produce new bioactive compounds (Sungthong and Nakaew 2015; Saygin et al. 2020d; Ay 2021). Eighty out of the 216 bioclusters (37%) detected in the genomes of the *Actinomadura* strains are associated with the production of new specialized metabolites. Similarly, 97 from a total of 204 bioclusters (48%) found in the genomes of the *Nonomuraea* strains were predicted to encode for new, uncharacterized compounds. *Micromonospora* and *Saccharopolyspora* strains are important sources of new antibiotics, especially ones of therapeutic value (Bérdy 2005; Hifnawy et al. 2020; Sayed et al. 2020b). However, their full potential in this respect only become apparent when it was shown that they had large genomes predicted to encode for new and uncharacterized specialized metabolites (Carro et al. 2018, 2019a, b). In this study, the genomes of the *Micromonospora* isolates, including *M. deserti* 13K206^T^ (Saygin et al. 2020c), contained between 15 and 49 BGCs, notably ones associated with the production of antibiotics, siderophores and terpenes, results similar to those reported by Carro et al. (2018). The genomes of *Sps. aridisoli* 16K404^T^, *Sps. elongata* 7K502^T^, *Sps. karakumensis* 5K548^T^ and *Sps. terrae* 16K309^T^ harbored between 18 and 29 bioclusters predicted to encoded for a broad range of specialized compounds, particularly antibiotics. Sixty one out of the 171 bioclusters detected in the genomes of the micromonosporae (36%) are associated with the synthesis of novel antibiotics; the corresponding numbers for the *Saccharopolyspora* strains; are 33 and 100 BGCs (33%), respectively. Isolate 7K107^T^, a Karakum Desert strain, was proposed as the type strain of *Desertactinospora gelatinilytica* (Saygin et al. 2019b) then transferred to the genus *Spongiactinospora* as *Spa. gelatinilytica* (Ay et al. 2020). The type strain of *Spa. gelatinilytica* has a large genome rich in BGCs. It is particularly interesting that 16 out of the 38 bioclusters detected in the genome of isolate 7K107^T^ did not show any similarity to known BGCs. The *Streptomyces* isolates, *Stm. cahuitamycinicus* 13K301^T^ and *Streptomyces* 8K308, have large genomes replete in BGCs. The genomes of these strains contained 34 and 29 bioclusters, 13 (38,2%) and 15 (51,7%) of which did not show any similarity to ones coding for known compounds. As expected, the genome of the moderately gifted *Jiangella*, *Kribbella*, *Microbacterium* and *Nocardioides* isolates have fewer BGCs than their extremely gifted counterparts. Members of these taxa rarely feature in bioprospecting campaigns although they are known to produce bioactive compounds (Gullo et al. 1988; Matson and Bush 1989; Matson et al. 1993; Alitalo et al. 2003; Li et al. 2004; El-Refai et al. 2011; Han et al. 2014; Jiao et al. 2017). In this study, 11 out of the 21 BGCs detected in the genomes of the *Jiangella* isolates did not show any similarity to known bioclusters thereby providing further evidence that members of this genus are a potential source of new antibiotics. Six out of the ten BGCs detected in the genome of *K. turkmenica* 16K104^T^ were novel whereas *Noc. turkmenicus* KC13^T^ contains six BGCs, 3 of which are novel, and a novel BGC in *Microbacterium* 5K110.

Enhanced crop production and control of plant pathogens are key factors in reducing threats to global food security (Boukhatem et al. 2022). Free-living and mutualistic bacteria use direct and indirect mechanisms to promote and protect plant growth (Hayat et al. 2010; Glick 2012; Nouioui et al. 2019). Comparative mining of the genomes of the isolates revealed the presence of genes associated with direct (phosphate solubilisation, phytohormone production) and indirect (synthesis of lytic enzymes and siderophores) mechanisms beneficial to the growth and protection of plants. Bacteria and fungi synthesize siderophores in response to iron limitation (Saha et al. 2016). These compounds chelate ferric iron (Fe^+ 3^) thereby making iron available for microbial and plant cells (Kloepper et al. 1980). Taxonomically diverse rhizobacteria enhance the growth of plants by making iron available to them through the synthesis of siderophores (Ahmed and Holmström 2014; Grobelak and Hiller 2017) whereas the ability of the latter to bind iron tightly is used to control phytopathogens by restricting its availability (Köhl et al. 2019). Karakum Desert isolates are quite capable of promoting and protecting plant growth properties (Tables S5 and S6). Isolates belonging to the genus *Micromonospora* have a genome rich in genes associated with siderophore production compared to other genera.

Bacteria in extreme habitats have developed ways of responding to environmental stress (Abdel-Mageed et al. 2020). In the present study, the genomes of the isolates were found to contain between 18 and 44 putative stress-related genes, notably ones associated with carbon starvation, drought tolerance, oxidative and osmotic stress, detoxification and temperature fluxes. Some genes associated with environmental adaptation are common to all of the isolates but others are either discontinuously distributed or strain specific (Table S7). Similar results have been reported for actinomycetes isolated from hyper-arid Atacama Desert soils (Busarakam et al. 2016). Under nutrient-limiting conditions, such as those in desert soils, bacteria need to acquire and store carbon. The genomes of most of the *Actinomadura* strains and all of the *Jiangella*, *Kribbella*, *Nocardioides*, *Nonomuraea*, *Saccharopolyspora* and *Streptomyces* isolates contained a gene (*cstA*) that encodes for starvation protein A, which activates peptide uptake thereby indicating that strains are adapted to low carbon conditions (Rasmussen et al. 2013). It is also notable that the genomes of the *Actinomadura*, *Nocardioides*, *Nonomuraea* and *Saccharopolyspora* isolates, *J. aurantiaca* 8K307^T^ and *Stm. cahuitamycinicus* 13K301^T^ contain *coxD* and *coxE* genes, which encode CO uptake thereby showing that these strains have the ability to function as chemolithotrophs (Lorite et al. 2000), as is the case with *Blastococcus*, *Geodermatophilus* and *Modestobacter* strains that are abundant in the Atacama Desert soils (Idris et al. 2017; Bull et al. 2018; Golińska et al. 2020).

## Conclusion

Novel, rare and gifted filamentous actinomycetes isolated from natural habitats, notably the extremebiosphere, remain a unique source of specialized metabolites of biotechnological importance, including antibiotics of clinical value and plant growth promoting compounds relevant to sustainable agriculture. Yet, the initial steps in natural product pipelines tend to be overshadowed by later stages of the process, such as chemical exploration of drug leads. Despite this, the selective isolation, dereplication, taxonomic characterization and screening of representative actinomycetes from neglected and unexplored extreme biomes provides a practical and effective way of selecting rare and novel isolates in the search for new bioactive compounds using state-of-the-art technologies, notably genome mining (Świecimska et al. 2023). In addition, advanced analytical procedures are now available to detect and dereplicate new specialized metabolites from complex biological extracts of selected isolates (Atanasov et al. 2021; Ossai et al. 2022) and marine sediments (Tuttle et al. 2019).

In the present study, the relevance of the early stages of natural product pipelines was revealed by the assignment of representative isolates from the Karakum Desert sampling sites to 17 genera, 11 families and eight orders of the phylum *Actinomycetota*. This extensive cultivable biodiversity included many novel and putatively new species belonging to rare taxa, such as members of the genera *Actinocorallia*, *Actinomadura*, *Jiangella*, *Nonomuraea* and *Spongiactinospora*, which encompasses strains which can be difficult to isolate from natural habitats. The isolation of members of such taxonomically diverse taxa reflects the use of a broad-range of selective media, as exemplified by the growth of novel and putatively novel *Nonomuraea* strains on SM1, SM3, marine, humic acid-vitamin, M1, non-sporulating, R2A, starch-casein and glycerol-asparagine agar. In contrast, isolates representing several taxa were isolated from plates of humic acid-vitamin which is known to be a relatively non-selective medium (Busarakam 2014; Idris 2016). This wealth of taxonomic data show that pristine Karakum Desert habitats are hot spots of filamentous actinomycetes that belong to taxa known to be a source of a chemically diverse specialized metabolites.

The results of the antimicrobial diffusion assays are particularly encouraging as nearly half of the representative isolates inhibited the growth of one or more of the reference strains. In general, these data are in line with those from corresponding results recorded for taxonomically diverse, filamentous actinomycetes isolated from hyper-arid and extreme hyper-arid Atacama Desert soils. The combined datasets shows that high hit rates can be achieved when dereplicated filamentous actinomycetes from arid desert habitats are examined in primary antimicrobial screens. Given the urgent need to find new antibiotics to control the spread of Gram-negative pathogens, it is particularly significant that *Enterococcus faecalis* ATCC 29,212 was inhibited by the *Actinomadura*, *Nonomuraea*, *Saccharopolyspora* and *Streptomyces* isolates. Also, *Actinomadura*, *Jiangella* and *Saccharopolyspora* isolates inhibited the growth of *Pseudomonas aeruginosa* ATCC 27,853, albeit less to.

The genomes of most of the novel and presumptive novel isolates representing ten of the genera were rich both in BGCs predicted to encode for an amazing array of specialized metabolites and genes associated with the production of compounds that promote and protect plant growth. Many of the BGCs were strain or taxon specific providing further evidence of the value of taxonomic approaches to drug discovery. Interestingly, the genomes of some of the isolates, such as the *Jiangella*, *Micromonospora*, *Nonomuraea* and *Saccharopolyspora* strains, contained bioclusters which did not show any similarity to ones expressing for known compounds thereby underscoring the significant of genome mining in the search for novel specialized metabolites. In contrast, the genomes of most of the strains, notably the *Jiangella* isolates, contained genes associated with phosphate metabolism whereas those predicted to encode for siderophores were discontinuously distributed across the genomes of the tested isolates.

It is not surprising that most extensive taxonomic diversity was shown by the isolates assigned to the genus *Streptomyces* as representatives of this taxon tend to be dominant in arid desert habitats. However, a unique feature of this culture-based study is that the representative streptomycetes were shown to belong to either putatively novel or rare *Streptomyces* species known or predicted to encode for new specialized metabolites. *Streptomyces asenjonii* and *Streptomyces leeuwenhoekii*, which were isolated from Atacama Desert, have capacity to produce new bioactive compounds with antibacterial, anti-cancer and anti-inflammatory properties. In this content, *Stm. cahuitamycinicus* 13K301^T^ and *Streptomyces* isolate 8K308 are of particular interest as most of the BGCs embedded in their genomes showed little, if any, similarity to ones coding for known compounds. These are significant developments as streptomycetes isolated from natural habitats remain the most important source of new chemically diverse antibiotics. Similar deductions can be drawn for the putatively novel and rare species that were shown to belong to the other genera, notably *Micromonospora*, *Nonomuraea* and *Saccharopolyspora*. Consequently, it can be concluded that the most tangible outcome of this culture-based bioprospecting strategy is the generation of a high quality library of novel and rare filamentous actinomycetes with the capacity to produce a multiplicity of novel specialized metabolites that can be exploited for useful purposes, such as drug discovery and the promotion and protection of plant growth. Further, work is required to build upon these developments not least the need to understand the ecological and evolutionary processes that drive the taxonomic and metabolic diversity of filamentous actinomycetes in Karakum Desert habitats.

It is encouraging that integrated strategies are being developed to improve natural product pipelines in order to promote the sustainable discovery and development of novel antibiotics (Miethke et al. 2021), and to foster an improved understanding of plant-microbe interactions to preserve natural ecosystems and to develop a more productive and sustainable agriculture (Riesco et al. 2022), where appropriate culture-dependent bioprospecting campaigns like the present one should be a part of such integrated strategies in order to address critical problems facing humankind, including the need to discovery a new generation of antibiotics to control multidrug-resistant microbial pathogens, bioinoculants to suppress the growth of phytopathogens, and the selection of filamentous actinomycetes that enhance plant growth, alleviate plant stress, reduce reliance on fertilizers and pesticides, and raise soil fertility.

Primary requirements for culture-dependent studies include access to hot spots of actinomycete diversity, associated culture-collections and genomic databases, as well as to chemical libraries of natural products. However, extreme biomes by their very nature are fragile, as witnessed by the destruction of microbial communities in the hyper-arid core of the Atacama Desert following unprecedented rainfall (Azua-Bustos et al. 2018). Consequently, stringent, enforceable measures are needed to protect extreme desert ecosystems, such as those in the Karakum Desert, from fossil fuel and mining interests along the lines promoted by Cockell and Jones (2009) as well as strategies to mitigate climate change. Indeed, it is in our interest as microbiologists to promote microbial conservation as an essential service to humankind.

## Electronic supplementary material

Below is the link to the electronic supplementary material.

Supplementary Material 1

## Data Availability

The datasets (genome and 16 S rRNA gene) generated and analysed for the current study are available in the National Center for Biotechnology Information (NCBI) repository (https://www.ncbi.nlm.nih.gov/).
