# Supplementary material for: Karakum desert: a unique source of cultivable novel and rare actinomycetes with a remarkable biosynthetic potential
Source: World J Microbiol Biotechnol. 2025 Jun 24;41(7):202. doi: 10.1007/s11274-025-04399-3 (PMC12187853; doi:10.1007/s11274-025-04399-3)
Supplement: Supplementary file 1 — Supplementary Material 1 [file 11274_2025_4399_MOESM1_ESM.pdf]

Supplementary File

Karakum Desert: A Unique Source of Cultivable Novel and Rare Actinomycetes with a Remarkable Biosynthetic Potential

Hayrettin Saygin[1,†], Nevzat Sahin[1], Michael Goodfellow[2]

[1]Department of Molecular Biology and Genetics, Faculty of Sciences, Ondokuz Mayis University, 55139, Samsun, Turkey

[2]School of Natural and Environmental Sciences, Newcastle University, Ridley Building 2, Newcastle upon Tyne NE1 7RU, UK

[†]**Corresponding authors:** Hayrettin Saygin

Tel : +903623121919 - Ext: 5436

Fax: +903624576081

E-mail: hayrettin.saygin@omu.edu.tr

Table S1. Accession numbers of the whole-genome sequences for the selected isolates.

Isolates	Accession numbers
Actinomadura sp. KC06	SMKT00000000
Actinomadura sp. KC216	SMJX00000000
Actinomadura sp. KC345	SMKH00000000
Actinomadura sp. 6K520	SMLC00000000
Actinomadura sp. 7K507	SMKK00000000
Actinomadura sp. 7K534	SMKB00000000
Jiangella ureilytica KC603^T	SMKL00000000
Jiangella asiatica 5K138^T	SMKZ00000000
Jiangella aurantiaca 8K307^T	SMLB00000000
Kribbella turkmenica 16K104^T	SMKR00000000
Microbacterium sp. 5K110	VBUM00000000
Micromonospora sp. KC207	SMKJ00000000
Micromonospora sp. KC213	SMKF00000000
Micromonospora sp. KC606	SMKN00000000
Micromonospora sp. KC721	SMJY00000000
Micromonospora sp. KC723	SMKD00000000
Micromonospora deserti 13K206^T	POUB00000000
Micromonospora sp. 15K316	SMKG00000000
Nocardoides turkmenicus KC13^T	JAALAA000000000
Nonomuraea deserti KC310^T	SMKO00000000
Nonomuraea aridisoli KC333^T	POUD00000000
Nonomuraea longispora KC401	VBUN00000000
Nonomuraea diastatica KC712^T	SMKP00000000
Nonomuraea longispora KC201^T	SMJZ00000000
Nonomuraea mesophila 6K102^T	SMLD00000000
Saccharopolyspora terrae 16K309^T	SMKS00000000
Saccharopolyspora aridisoli 16K404^T	SMKV00000000
Saccharopolyspora karakumensis 5K548^T	SMLA00000000
Saccharopolyspora elongata 7K502^T	SMKW00000000
Spongiactinospora gelatinilytica 7K107^T	POUA00000000
Streptomyces cahuitamycinicus 13K301^T	POUC00000000
Streptomyces sp. 8K308	SMKC00000000

Table S2. Assignment of isolates to multi- and single-membered colour-groups (n = 459 isolates).

Colour group	Aerial spore mass colour	Substrate mycelial colour	Colour of any diffusible pigments	Isolate codes*
**A. Multi-membered colour-groups**				
1.	-	Black	Dark olive	**5K221**, KC914
2.	Bluish white	Blackish green	Moderate yellowish brown	**4K405, 5K103, 12K506,** KC337, KC503
3.	-	Blackish red	Very deep red	6K511, 7K524, **8K103, KC202**, **KC605**, KC703
4.	-	Brilliant orange yellow	-	***5K110, 5K208,** 5K219, 7K509, 8K209, 10K201, ***13K206^{T}**, **KC705, KC725**, **KC728**, **KC729**
5.	Yellowish white	Brilliant orange yellow	-	5K546, **6K504, 6K519, 7K528,** KC16
6.	Pinkish white	Brilliant yellow	-	4K201, **5K505,** 5K527, **8K205, 12K203,** 16K206, **16K501**
7.	White	Brilliant yellow	-	12K104, **KC916**
8.	Light gray	Brownish black	-	**5K109,** 6K301, 7K302, **10K102**, **16K211**
9.	Medium gray	Brownish black	-	5K119, **5K135,** 5K315, **6K525, 11K502, KC319**, KC521
10.	-	Brownish orange	-	5K554, **15K303, 16K405**
11.	Grayish pink	Brownish orange	-	**3K201,** 10K202
12.	Moderate bluish green	Dark bluish green	-	**6K501,** 6K510
13.	-	Dark brown	Moderate brown	7K103, **10K305**
14.	Light gray	Dark brown	Light brown	**5K417,** 6K402, **7K304**
15.	White	Dark brown	Light brown	***13K301^{T},** 13K303
16.	-	Dark grayish reddish brown	Deep brown	**4K205,** 8K101, 13K101, 13K108, **13K111, 13K203, 15K101**
17.	-	Dark grayish yellow	-	**5K316,** 10K301
18.	-	Dark grayish yellow	Grayish yellow	***KC213**, KC704, KC718
19.	Light gray	Dark grayish yellow	-	7K303, 8K305, **10K402, 12K302,** 14K303, 14K304, **14K501, 16K303,** 16K310
20.	-	Dark olive brown	-	**5K118, 7K104,** 12K404, 13K202, **13K208, 15K103**
21.	-	Dark olive brown	Moderate olive brown	3K301, **6K103, 7K305**
22.	-	Dark olive green	Grayish green	12K503, **KC708**
23.	-	Dark orange yellow	-	10K213, 12K502, 15K102, **KC204**, **KC205**, **KC215**, ***KC606**, **KC707**, ***KC721, *KC723**
24.	Medium gray	Dark red	-	***KC333^{T}**, KC514, KC517
25.	-	Dark reddish brown	-	***15K316, 16K103**, KC711, KC904
26.	-	Dark reddish brown	Light reddish brown	KC203, KC309, KC341, KC414, KC508, KC519, **KC713**, **KC730**, **KC929**

27.	-	Dark yellow	-	7K101, **7K111,** KC722, **8K206, 10K209**
28.	Light gray	Dark yellow	-	**3K203, 7K201,** 7K203, KC731
29.	-	Dark yellowish brown	Light brown	**5K101,** KC214, KC314
30.	Pale blue	Dark yellowish brown	Light yellowish brown	3K103, **KC416, KC506**
31.	-	Deep brown	Strong brown	**7K529, 8K508, KC340**, KC402, **KC501**, KC513, KC912, **KC918**, KC923
32.	Pale blue	Deep greenish yellow	-	7K403, **KC421**
33.	White	Deep orange	Light yellowish brown	**6K521,** 7K503, 8K511, **10K511, 10K521**
34.	-	Deep orange yellow	-	**4K102, *16K404^{T},** KC312, **KC608,** KC911
35.	Dark grayish yellow	Deep orange yellow	Vivid yellow	12K305, 15K307, **KC11**, **KC12**, **KC407**
36.	White	Deep reddish brown	Light yellowish brown	KC338, **KC406**, **KC727**, **KC921**
37.	-	Deep yellowish brown	Light yellowish brown	6K516, 10K526, **KC507**, **KC509**
38.	Light gray	Deep yellowish brown	Light olive gray	5K504, **5K520, 5K526, 7K501,** 7K508, 7K512, **7K513,** 10K509, 13K502, KC321
39.	-	Earlier brilliant yellow	-	16K402, **KC209**, **KC910**
40.	-	Earlier dark orange yellow	-	12K205, **16K403**
41.	-	Earlier dark yellow	-	**4K203,** 5K508, **10K507, 10K514,** 13K304, 13K306, **14K201, 14K203,** 16K301
42.	-	Earlier deep yellow	-	**3K402,** 14K401, 15K305, **15K402,** KC701
43.	-	Earlier grayish yellow	-	**5K125, 5K122**, 5K411, **8K102**, **10K101,** 10K522
44.	-	Earlier light orange	-	**1K501, 7K519,** 15K315
45.	-	Earlier pale yellow	-	5K137, 5K510, **5K418**, 7K521, 16K106, 16K304, 16K308, **KC304, KC323**
46.	Yellowish white	Earlier strong orange	-	7K402, **10K210**
47.		Earlier strong yellow		***6K520, *KC345**
48.	-	Earlier strong yellowish brown	-	10K404, **11K402,** 15K309, 15K401, **15K408, 16K205**
49.	-	Earlier yellowish white	-	**4K403B,** 5K542
50.	Light gray	Grayish brown	-	**5K114,** 6K101, 6K522, 7K514, 10K103, **11K101**, **13K103**, **13K109,** 15K501, **16K102**, **16K207,** 16K209, KC326
51.	-	Grayish red	-	7K105, **16K311**
52.	-	Grayish yellowish brown	-	**5K531, 5K532,** 6K507, 6K513, 10K519
53.	White	Light brown	Light grayish brown	**13K403,** 15K404, **16K401**
54.	Yellowish white	Light brown	-	**6K502,** 8K502, 11K503
55.	-	Light grayish red	-	**13K105,** KC404
56.	-	Light orange yellow	-	5K111, **5K133, 5K313**
57.	White	Light orange yellow	-	**5K515, 6K523, 7K401,** KC307, KC424

58.	-	Light yellow	-	***KC06,** KC206, ***KC216**
59.	Pinkish white	Light yellow	-	7K511, **KC320**
60.	White	Light yellow	-	10K505, **12K109, KC903**
61.	Medium gray	Moderate brown	Light brown	**8K301,** 10K304
62.	White	Moderate brown	Light brown	**10K307,** KC920
63.	-	Moderate yellow	-	5K311, 10K303, **KC311**, **KC316**, **KC325**, KC334, **KC502**, **KC510**, KC516, KC520
64.	Light gray	Moderate yellow	-	**6K201,** 7K202, 10K211, 13K201, **16K210**
65.	Pinkish white	Moderate yellow	-	KC714, **12K107, 16K107**
66.	White	Moderate yellow	-	**7K109,** KC328
67.	-	Moderate yellowish brown	-	5K307, 5K309, ***KC310^{T}**, **KC327**, **KC518**
68.	Light gray	Moderate yellowish brown	-	5K306, **5K312,** 6K304, **7K522,** 13K302, 13K305, **15K302,** 15K310
69.	Yellowish white	Moderate yellowish brown	-	**5K551,** 10K503, 10K104, **KC15**, **KC410**
70.	-	Pale orange yellow	-	**5K134, 5K320, 5K322, 7K532,** KC322
71.	White	Pale yellow	-	***5K138^{T},** 5K539, 7K112, 7K526, ***7K534**
72.	-	Reddish black	-	5K522, **5K544,** 7K518, **7K530, 8K503,** 10K508, **10K527, 12K108**
73.	-	Strong brown	Strong yellowish brown	5K108, **5K131, 5K132**
74.	Light pink	Strong brown	-	5K201, **10K208, 10K212**
75.	Yellowish white	Strong orange	-	5K536, 5K553, **6K505**, **7K517, 7K520**
76.	-	Strong orange yellow	-	4K207, **5K222,** 5K419, **5K521, 10K510**
77.	-	Strong reddish orange	-	**5K506,** 5K523, 5K545, 5K547, **6K514, *7K502^{T}**
78.	Gray	Strong reddish orange	-	5K552, **10K516**
79.	-	Strong yellow	-	**KC301**, **KC504**, KC515
80.	-	Strong yellowish brown	-	**4K104**, 5K127, 5K120, **7K110**
81.	Medium gray	Very dark red	Light reddish brown	10K512, **KC403**, **KC907**
82.	White	Very deep red	Grayish reddish orange	3K302, 6K302, ***KC201^{T}**, **KC306**, KC331, **KC332,** KC505, **KC706**, **KC924**
83.	White	Vivid orange	-	**1K503, 5K116,** 5K126, 5K310, ***8K307^{T}**
84.	Bluish white	Vivid red	-	KC329, **KC512**
85.	-	Vivid reddish orange	-	5K204, 6K401, **12K402, *KC207**, **KC208**
86.	-	Vivid yellow	-	5K105, 5K517, 5K528, **6K515, 7K527, 7K525,** 7K531, **7K537, 10K502, 10K504**
87.	White	Vivid yellow	Light orange yellow	**7K301,** 10K306, 14K301, **16K306, KC18**
88.	White	Yellowish white	-	**5K502, 5K516, 5K550**

B. Single-membered colour-groups

89.	Light gray	Brownish orange	Light orange yellow	**KC426**
90.	-	Dark brown	Grayish brown	**3K401**
91.	-	Dark brown	-	**15K301**
92.	Pale yellow	Dark orange yellow	-	**KC103**
93.	White	Dark orange yellow	Light orange yellow	**KC408**
94.	-	Dark reddish brown	Moderate orange yellow	***KC401**
95.	-	Dark reddish orange	-	**7K523**
96.	-	Deep orange	Dark brown	**KC601**
97.	White	Deep orange yellow	Brilliant orange yellow	**KC930**
98.	-	Deep yellow	-	**5K209**
99.	-	Deep yellowish brown	-	**13K204**
100.	White	Earlier light orange yellow	-	**5K555**
101.		Earlier light yellow		***5K548^{T}**
102.		Earlier moderate yellow		***6K102^{T}**
103.	-	Earlier strong orange yellow	-	**7K533**
104.	-	Earlier strong reddish orange	-	**16K305**
105.	-	Grayish yellow	-	**15K203**
106.	White	Grayish yellow	-	**4K202**
107.	-	Light grayish yellowish brown	-	**4K403A**
108.	-	Light olive brown	-	**16K105**
109.	White	Light olive brown	-	**KC101**
110.	-	Light orange	-	***16K309^{T}**
111.	-	Light yellowish brown	-	***8K308**
112.	-	Light yellowish pink	-	***7K507**
113.	-	Moderate orange yellow	-	**8K306**
114.	White	Moderate reddish brown	-	***7K107^{T}**
115.	-	Moderate reddish orange	-	**7K515**
116.	-	Olive black	Olive gray	**5K409**
117.	Light gray	Pale greenish yellow	-	**4K301**
118.	White	Pale orange yellow	-	***16K104^{T}**
119.		Pale yellow		***KC603^{T}**
120.	-	Strong brown	Light brown	***KC712^{T}**
121.	White	Strong brown	Light brown	**5K524**

122.	-	Very deep red	-	**7K504**
123.	-	Yellowish white	-	***KC13^{T}**
**Non-actinomycete isolates**				
124.	-	Brilliant greenish yellow	-	5K303, **5K501**
125.	-	Brilliant yellow	-	**7K506**
126.	-	Deep pink	-	5K305, 5K324, **10K302**
127.	-	Grayish reddish orange	-	**3K102**
128.	White	Pale yellowish white	-	**KC615**
129.	-	Pale yellowish white	-	**7K510**, 12K103, **13K307**
130.	-	Strong pink	-	**5K112, 5K207,** 5K301
131.	-	Vivid greenish yellow	-	3K202, **5K212**
132.	-	Vivid red	-	**1K502**

Isolates given in bold were included in the 16S rRNA gene sequencing studies; those included in the whole genome sequencing analyses on asterisk.

*The isolate codes are explained in the footnote to Table 5.

Key: -, isolates that did not form aerial hyphae and/or produce diffusible pigments

Table S3. 16S rRNA gene sequence similarity values between isolates and their nearest phylogenetic neighbours.

Results in bold indicate isolates considered to be novel as their sequence similarity to the type strain of their closest phylogenetic neighbour was at or below the 99.3% threshold. (n = 270 isolates)

Isolates	Colour group	Most closely related type strains	Similarity (%)	Nucleotide differences/total number of nucleotides	Length of 16S rRNA genes
**5K502**	**88**	***Actinocorallia aurantiaca* JCM 8201^T**	**99.16**	**12/1424**	**1476**
5K516	88	*Actinocorallia aurantiaca* JCM 8201^T	99.51	7/1425	1472
**5K550**	**88**	***Actinocorallia libanotica* IFO 14095^T**	**99.07**	**13/1402**	**1472**
**KC06**	**58**	***Actinomadura darangshiensis* DSLS-70^T**	**98.52**	**21/1416**	**1481**
**KC216**	**58**	***Actinomadura darangshiensis* DSLS-70^T**	**98.80**	**17/1417**	**1482**
**KC345**	**47**	***Actinomadura bangladeshensis* 3-46-b3^T**	**98.41**	**23/1443**	**1493**
**KC608**	**34**	***Actinomadura bangladeshensis* 3-46-b3^T**	**98.82**	**17/1442**	**1483**
5K515	57	*Actinomadura geliboluensis* A8036^T	100.00	0/1441	1481
5K555	100	*Actinomadura deserti* BMP B8004^T	99.44	8/1440	1484
6K515	86	*Actinomadura cremea* JCM 3308^T	99.93	1/1428	1481
**6K520**	**47**	***Actinomadura livida* JCM 3387^T**	**99.03**	**14/1442**	**1495**
6K521	33	*Actinomadura deserti* BMP B8004^T	99.44	8/1440	1478
6K523	57	*Actinomadura geliboluensis* A8036^T	100.00	0/1441	1481
7K401	57	*Actinomadura geliboluensis* A8036^T	100.00	0/1441	1481
**7K507**	**112**	***Actinomadura bangladeshensis* 3-46-b3^T**	**98.89**	**16/1443**	**1489**
**7K515**	**115**	***Actinomadura darangshiensis* DSLS-70^T**	**98.52**	**21/1416**	**1480**
7K525	86	*Actinomadura cremea* JCM 3308^T	99.86	2/1428	1483
7K527	86	*Actinomadura cremea* JCM 3308^T	99.93	1/1428	1481
**7K533**	**103**	***Actinomadura cremea* JCM 3308^T**	**99.09**	**13/1429**	**1482**
7K534	71	*Actinomadura livida* JCM 3387^T	99.31	10/1444	1494
7K537	86	*Actinomadura cremea* JCM 3308^T	99.93	1/1428	1482
10K502	86	*Actinomadura cremea* JCM 3308^T	99.93	1/1428	1479
10K504	86	*Actinomadura cremea* JCM 3308^T	99.93	1/1428	1483
10K511	33	*Actinomadura sediminis* YIM M 10931^T	100.00	0/1445	1486
10K521	33	*Actinomadura deserti* BMP B8004^T	99.38	9/1440	1482
**4K403B**	**49**	***Agromyces subbeticus* DSM 16689^T**	**98.76**	**18/1450**	**1495**
KC603^T	119	*Jiangella mangrovi* 3SM4-07^T	99.44	8/1441	1477
1K503	83	*Jiangella rhizosphaerae* NEAU-YY265^T	99.72	4/1442	1484
5K116	83	*Jiangella rhizosphaerae* NEAU-YY265^T	99.72	4/1442	1473
5K138^T	71	*Jiangella alba* DSM 45237^T	99.45	8/1443	1483
8K307^T	83	*Jiangella rhizosphaerae* NEAU-YY265^T	99.72	4/1442	1473
**16K104^T**	**118**	***Kribbella albertanoniae* BC640^T**	**99.17**	**12/1442**	**1480**

7K109	66	*Kribbella pittospori* PIP 158^{T}	99.93	1/1346	1484
**5K110**	**4**	***Microbacterium testaceum* DSM 20166^{T}**	**99.17**	**11/1444**	**1485**
KC202	3	*Micromonospora costi* CS1-12^{T}	99.65	5/1438	1475
**KC204**	**23**	***Micromonospora halophytica* DSM 43171^{T}**	**99.24**	**11/1439**	**1476**
**KC205**	**23**	***Micromonospora halophytica* DSM 43171^{T}**	**99.24**	**11/1439**	**1476**
KC207	85	*Micromonospora fluostatini* PWB-003^{T}	99.44	8/1437	1473
KC208	85	*Micromonospora fluostatini* PWB-003^{T}	99.44	8/1437	1474
**KC213**	**18**	***Micromonospora halophytica* DSM 43171^{T}**	**99.17**	**12/1439**	**1475**
**KC215**	**23**	***Micromonospora palomenae* NEAU-CX1^{T}**	**99.10**	**13/1439**	**1480**
KC605	3	*Micromonospora costi* CS1-12^{T}	99.65	5/1438	1475
**KC606**	**23**	***Micromonospora costi* CS1-12^{T}**	**99.03**	**14/1438**	**1481**
**KC707**	**23**	***Micromonospora halophytica* DSM 43171^{T}**	**99.24**	**11/1439**	**1479**
**KC708**	**22**	***Micromonospora halophytica* DSM 43171^{T}**	**99.24**	**11/1439**	**1480**
**KC721**	**23**	***Micromonospora palomenae* NEAU-CX1^{T}**	**99.10**	**13/1439**	**1477**
**KC723**	**23**	***Micromonospora inositola* DSM 43819^{T}**	**99.03**	**14/1439**	**1476**
4K205	16	*Micromonospora costi* CS1-12^{T}	99.65	5/1438	1478
5K118	20	*Micromonospora saelicesensis* Lupac 09^{T}	99.79	3/1437	1479
5K221	1	*Micromonospora zamorensis* DSM 45600^{T}	100.00	0/1439	1476
5K409	116	*Micromonospora citrea* DSM 43903^{T}	99.79	3/1437	1477
5K544	72	*Micromonospora citrea* DSM 43903^{T}	99.51	7/1437	1479
7K104	20	*Micromonospora saelicesensis* Lupac 09^{T}	99.79	3/1437	1477
7K504	122	*Micromonospora aurantiaca* ATCC 27029^{T}	100.00	0/1437	1467
7K530	72	*Micromonospora citrea* DSM 43903^{T}	99.51	7/1437	1476
8K103	3	*Micromonospora costi* CS1-12^{T}	99.65	5/1438	1470
8K503	72	*Micromonospora citrea* DSM 43903^{T}	99.51	7/1437	1478
10K527	72	*Micromonospora citrea* DSM 43903^{T}	99.51	7/1437	1479
12K108	72	*Micromonospora citrea* DSM 43903^{T}	99.51	7/1437	1478
12K402	85	*Micromonospora fluostatini* PWB-003^{T}	99.51	7/1437	1477
13K111	16	*Micromonospora costi* CS1-12^{T}	99.65	5/1438	1481
13K203	16	*Micromonospora costi* CS1-12^{T}	99.65	5/1438	1476
**13K206^{T}**	**4**	***Micromonospora nigra* DSM 43818^{T}**	**98.54**	**21/1438**	**1480**
13K208	20	*Micromonospora saelicesensis* Lupac 09^{T}	99.79	3/1437	1478
15K101	16	*Micromonospora costi* CS1-12^{T}	99.65	5/1438	1478
15K103	20	*Micromonospora saelicesensis* Lupac 09^{T}	99.79	3/1437	1475
15K303	10	*Micromonospora saelicesensis* Lupac 09^{T}	99.72	4/1437	1478
15K316	25	*Micromonospora avicenniae* DSM 45758^{T}	99.37	9/1437	1474
16K103	25	*Micromonospora avicenniae* DSM 45758^{T}	99.37	9/1437	1478
16K305	104	*Micromonospora fiedleri* MG-37^{T}	99.57	6/1381	1462
16K405	10	*Micromonospora vinacea* GUI63^{T}	99.72	4/1418	1474

KC209	39	*Nocardia carnea* NBRC 14403^T	99.58	6/1439	1483
KC320	59	*Nocardia carnea* NBRC 14403^T	99.58	6/1439	1482
KC910	39	*Nocardia carnea* NBRC 14403^T	99.58	6/1439	1482
KC916	7	*Nocardia carnea* NBRC 14403^T	99.58	6/1439	1480
6K504	5	*Nocardia flavorosea* NBRC 108225^T	99.44	8/1439	1480
6K505	75	*Nocardia carnea* NBRC 14403^T	100.00	0/1439	1481
6K519	5	*Nocardia flavorosea* NBRC 108225^T	99.72	4/1439	1477
7K517	75	*Nocardia carnea* NBRC 14403^T	99.93	1/1439	1473
7K520	75	*Nocardia carnea* NBRC 14403^T	99.72	4/1439	1481
7K528	5	*Nocardia carnea* NBRC 14403^T	99.72	4/1439	1480
**KC13^T**	123	***Nocardioides luteus* KCTC 9575^T**	**99.30**	**10/1436**	**1473**
3K402	42	*Nocardiopsis umidischolae* 66/93^T	100.00	0/1441	1488
5K222	76	*Nocardiopsis dassonvillei* subsp. *dassonvillei* DSM 43111^T	100.00	0/1455	1496
5K521	76	*Nocardiopsis dassonvillei* subsp. *dassonvillei* DSM 43111^T	100.00	0/1455	1497
10K510	76	*Nocardiopsis dassonvillei* subsp. *dassonvillei* DSM 43111^T	100.00	0/1455	1496
15K402	42	*Nocardiopsis umidischolae* 66/93^T	100.00	0/1441	1497
16K403	40	*Nocardiopsis trehalosi* NBRC 14201^T	100.00	0/1453	1494
**KC201^T**	82	***Nonomuraea salmonea* DSM 43678^T**	**97.98**	**29/1437**	**1479**
**KC306**	82	***Nonomuraea salmonea* DSM 43678^T**	**97.98**	**29/1433**	**1465**
**KC310^T**	67	***Nonomuraea candida* HMC10^T**	**98.07**	**27/1398**	**1481**
**KC327**	67	***Nonomuraea candida* HMC10^T**	**98.07**	**27/1398**	**1489**
**KC332**	82	***Nonomuraea salmonea* DSM 43678^T**	**97.98**	**29/1437**	**1479**
**KC333^T**	24	***Nonomuraea maritima* FXJ7.203^T**	**98.93**	**15/1397**	**1403**
**KC401**	94	***Nonomuraea salmonea* DSM 43678^T**	**98.12**	**27/1437**	**1479**
**KC406**	36	***Nonomuraea salmonea* DSM 43678^T**	**97.98**	**29/1437**	**1479**
**KC507**	37	***Nonomuraea candida* HMC10^T**	**98.07**	**27/1398**	**1487**
**KC509**	37	***Nonomuraea candida* HMC10^T**	**98.05**	**27/1388**	**1466**
**KC512**	84	***Nonomuraea maritima* FXJ7.203^T**	**98.93**	**15/1397**	**1403**
**KC518**	67	***Nonomuraea candida* HMC10^T**	**98.07**	**27/1398**	**1481**
**KC706**	82	***Nonomuraea salmonea* DSM 43678^T**	**97.98**	**29/1433**	**1465**
**KC712^T**	120	***Nonomuraea candida* HMC10^T**	**98.07**	**27/1398**	**1480**
**KC713**	26	***Nonomuraea salmonea* DSM 43678^T**	**98.12**	**27/1433**	**1465**
**KC727**	36	***Nonomuraea salmonea* DSM 43678^T**	**97.98**	**29/1437**	**1488**
**KC730**	26	***Nonomuraea salmonea* DSM 43678^T**	**98.12**	**27/1437**	**1487**
**KC921**	36	***Nonomuraea salmonea* DSM 43678^T**	**97.98**	**29/1437**	**1487**
**KC924**	82	***Nonomuraea salmonea* DSM 43678^T**	**97.98**	**29/1437**	**1485**
**KC929**	26	***Nonomuraea salmonea* DSM 43678^T**	**98.12**	**27/1433**	**1465**
**1K501**	44	***Nonomuraea ceibae* XMU 110^T**	**98.82**	**17/1436**	**1485**
4K104	80	*Nonomuraea glycinis* NEAU-BB2C19^T	99.79	3/1444	1455

4K202	106	*Nonomuraea flavida* ex1^T	99.51	7/1439	1487
4K403A	107	*Nonomuraea glycinis* NEAU-BB2C19^T	99.65	5/1444	1486
**5K134**	70	***Nonomuraea maheshkhaliensis* 16-5-14^T**	**98.75**	**18/1441**	**1483**
5K320	70	*Nonomuraea glycinis* NEAU-BB2C19^T	99.72	4/1443	1485
**5K322**	70	***Nonomuraea maheshkhaliensis* 16-5-14^T**	**98.75**	**18/1441**	**1487**
**6K102^T**	102	***Nonomuraea salmonea* DSM 43678^T**	**98.33**	**24/1437**	**1484**
7K110	80	*Nonomuraea glycinis* NEAU-BB2C19^T	99.72	4/1443	1481
7K519	44	*Nonomuraea ceibae* XMU 110^T	99.79	3/1428	1472
7K523	95	*Nonomuraea harbinensis* NEAU-yn31^T	99.65	5/1447	1487
7K532	70	*Nonomuraea glycinis* NEAU-BB2C19^T	99.65	5/1444	1486
**8K306**	113	***Nonomuraea jabiensis* A4036^T**	**98.96**	**15/1441**	**1483**
**15K203**	105	***Nonomuraea polychroma* DSM 43925^T**	**98.41**	**23/1443**	**1484**
**15K301**	91	***Nonomuraea turkmeniaca* DSM 43926^T**	**98.73**	**18/1416**	**1480**
**KC601**	96	***Plantactinospora veratri* NEAU-FHS4^T**	**98.68**	**19/1438**	**1482**
KC705	4	*Plantactinospora veratri* NEAU-FHS4^T	99.51	7/1438	1478
KC725	4	*Plantactinospora veratri* NEAU-FHS4^T	99.51	7/1438	1478
KC728	4	*Plantactinospora veratri* NEAU-FHS4^T	99.44	8/1438	1472
KC729	4	*Plantactinospora veratri* NEAU-FHS4^T	99.51	7/1438	1475
5K208	4	*Plantactinospora veratri* NEAU-FHS4^T	99.50	7/1404	1409
KC301	79	*Pseudonocardia cypriaca* KT2142^T	99.51	7/1442	1486
KC304	45	*Pseudonocardia adelaidensis* EUM 221^T	99.36	9/1411	1487
KC311	63	*Pseudonocardia zijingensis* 6330^T	99.58	6/1418	1480
KC316	63	*Pseudonocardia adelaidensis* EUM 221^T	99.57	6/1411	1483
KC323	45	*Pseudonocardia adelaidensis* EUM 221^T	99.43	8/1411	1484
KC325	63	*Pseudonocardia adelaidensis* EUM 221^T	99.57	6/1411	1483
KC502	63	*Pseudonocardia zijingensis* 6330^T	99.51	7/1419	1483
KC504	79	*Pseudonocardia cypriaca* KT2142^T	99.51	7/1442	1484
KC510	63	*Pseudonocardia adelaidensis* EUM 221^T	99.57	6/1411	1482
5K133	56	*Pseudonocardia hierapolitana* PM2084^T	99.93	1/1443	1480
5K313	56	*Pseudonocardia hierapolitana* PM2084^T	100.00	0/1443	1479
5K418	45	*Pseudonocardia kunmingensis* YIM 63158^T	99.35	9/1388	1489
6K501	12	*Saccharomonospora azurea* NA-128^T	100.00	0/1447	1488
KC101	109	*Saccharopolyspora erythraea* NRRL 2338^T	99.93	1/1445	1479
KC103	92	*Saccharopolyspora erythraea* NRRL 2338^T	100.00	0/1445	1485
KC408	93	*Saccharopolyspora erythraea* NRRL 2338^T	99.93	1/1445	1482
4K102	34	*Saccharopolyspora endophytica* YIM 61095^T	99.65	5/1445	1483
5K506	77	*Saccharopolyspora shandongensis* 88^T	99.44	8/1417	1486
5K548^T	101	*Saccharopolyspora pathumthaniensis* S582^T	99.79	3/1445	1486
6K514	77	*Saccharopolyspora shandongensis* 88^T	99.58	6/1417	1489

7K502^{T}	77	*Saccharopolyspora shandongensis* 88^{T}	99.44	8/1417	1486
16K309^{T}	110	*Saccharopolyspora pathumthaniensis* S582^{T}	99.65	5/1445	1460
16K404^{T}	34	*Saccharopolyspora endophytica* YIM 61095^{T}	99.65	5/1410	1415
**7K107^{T}**	114	***Spongiactinospora rosea* LHW63015^{T}**	**98.69**	**19/1452**	**1493**
KC11	35	*Streptomyces ambofaciens* ATCC 23877^{T}	99.59	6/1449	1491
KC12	35	*Streptomyces ambofaciens* ATCC 23877^{T}	99.59	6/1449	1488
KC15	69	*Streptomyces kurssanovii* NBRC 13192^{T}	99.93	1/1444	1486
KC18	87	*Streptomyces luteus* TRM 45540^{T}	99.93	1/1450	1487
KC319	9	*Streptomyces ambofaciens* ATCC 23877^{T}	99.59	6/1449	1491
KC340	31	*Streptomyces thinghirensis* DSM 41919^{T}	99.38	9/1447	1487
KC403	81	*Streptomyces ambofaciens* ATCC 23877^{T}	99.59	6/1449	1490
KC407	35	*Streptomyces ambofaciens* ATCC 23877^{T}	99.59	6/1449	1487
KC410	69	*Streptomyces kurssanovii* NBRC 13192^{T}	99.93	1/1444	1487
KC416	30	*Streptomyces kurssanovii* NBRC 13192^{T}	100.00	0/1444	1489
KC421	32	*Streptomyces kurssanovii* NBRC 13192^{T}	99.93	1/1444	1484
KC426	89	*Streptomyces kurssanovii* NBRC 13192^{T}	99.93	1/1444	1488
**KC501**	31	***Streptomyces thinghirensis* DSM 41919^{T}**	**99.10**	**13/1447**	**1488**
KC506	30	*Streptomyces ambofaciens* ATCC 23877^{T}	99.59	6/1449	1491
KC903	60	*Streptomyces ambofaciens* ATCC 23877^{T}	99.86	2/1449	1488
KC907	81	*Streptomyces ambofaciens* ATCC 23877^{T}	99.59	6/1449	1490
KC918	31	*Streptomyces thinghirensis* DSM 41919^{T}	99.38	9/1447	1489
KC930	97	*Streptomyces thinghirensis* DSM 41919^{T}	99.31	10/1447	1488
3K201	11	*Streptomyces roseolilacinus* NBRC 12815^{T}	99.51	7/1441	1492
3K203	28	*Streptomyces atrovirens* NRRL B-16357^{T}	99.93	1/1449	1487
**3K401**	90	***Streptomyces krungchingensis* KC-035^{T}**	**98.48**	**22/1448**	**1488**
4K203	41	*Streptomyces roseolilacinus* NBRC 12815^{T}	99.51	7/1441	1493
**4K301**	117	***Streptomyces manganisoli* MK44^{T}**	**99.29**	**10/1405**	**1496**
4K405	2	*Streptomyces rochei* NRRL B-2410^{T}	100.00	0/1448	1490
**5K101**	29	***Streptomyces xantholiticus* NBRC 13354^{T}**	**99.23**	**11/1435**	**1488**
**5K103**	2	***Streptomyces coeruleofuscus* NBRC 12757^{T}**	**99.17**	**12/1446**	**1485**
5K109	8	*Streptomyces calvus* ISP 5010^{T}	100.00	0/1448	1490
5K114	50	*Streptomyces calvus* ISP 5010^{T}	100.00	0/1448	1487
5K122	43	*Streptomyces roseolilacinus* NBRC 12815^{T}	99.51	7/1441	1489
5K125	43	*Streptomyces spinoverrucosus* NBRC 14228^{T}	99.72	4/1445	1486
5K131	73	*Streptomyces thinghirensis* DSM 41919^{T}	99.36	9/1405	1410
5K132	73	*Streptomyces thinghirensis* DSM 41919^{T}	99.36	9/1403	1407
5K135	9	*Streptomyces calvus* ISP 5010^{T}	99.86	2/1448	1485
**5K209**	98	***Streptomyces barkulensis* RC 1831^{T}**	**98.65**	**19/1407**	**1490**
5K312	68	*Streptomyces roseolilacinus* NBRC 12815^{T}	99.51	7/1441	1493

**5K316**	17	***Streptomyces coeruleofuscus* NBRC 12757^T**	**99.17**	**12/1446**	**1488**
5K417	14	*Streptomyces nitrosporeus* NBRC 3362^T	99.58	6/1445	1488
5K505	6	*Streptomyces marokkonensis*Ap1^T	99.86	2/1449	1486
5K520	38	*Streptomyces fradiae* DSM 40063^T	99.72	4/1450	1491
5K524	121	*Streptomyces xantholiticus* NBRC 13354^T	99.37	9/1436	1488
5K526	38	*Streptomyces rubrogriseus* LMG 20318^T	99.79	3/1448	1489
5K531	52	*Streptomyces marokkonensis* Ap1^T	99.65	5/1449	1491
5K532	52	*Streptomyces marokkonensis* Ap1^T	99.86	2/1449	1493
5K551	69	*Streptomyces kurssanovii* NBRC 13192^T	99.93	1/1444	1489
6K103	21	*Streptomyces spinoverrucosus* NBRC 14228^T	99.93	1/1445	1488
6K201	64	*Streptomyces atrovirens* NRRL B-16357^T	99.93	1/1449	1486
6K502	54	*Streptomyces marokkonensis* Ap1^T	99.38	9/1449	1489
6K525	9	*Streptomyces calvus* ISP 5010^T	100.00	0/1448	1490
7K111	27	*Streptomyces calvus* ISP 5010^T	99.93	1/1448	1486
7K201	28	*Streptomyces calvus* ISP 5010^T	100.00	0/1448	1485
7K301	87	*Streptomyces luteus* TRM 45540^T	100.00	0/1450	1491
7K304	14	*Streptomyces nitrosporeus* NBRC 3362^T	99.58	6/1445	1489
7K305	21	*Streptomyces spinoverrucosus* NBRC 14228^T	99.65	5/1445	1488
7K501	38	*Streptomyces rubrogriseus* LMG 20318^T	100.00	0/1448	1486
7K513	38	*Streptomyces marokkonensis* Ap1^T	99.38	9/1449	1492
7K522	68	*Streptomyces luteus* TRM 45540^T	99.66		1490
7K529	31	*Streptomyces leeuwenhoekii* C34^T	99.86	2/1450	1488
8K102	43	*Streptomyces roseolilacinus* NBRC 12815^T	99.51	7/1441	1493
8K205	6	*Streptomyces roseolilacinus* NBRC 12815^T	99.51	7/1441	1490
8K206	27	*Streptomyces calvus* ISP 5010^T	100.00	0/1448	1487
**8K301**	61	***Streptomyces coeruleofuscus* NBRC 12757^T**	**99.10**	**13/1446**	**1494**
**8K308**	111	***Streptomyces hainanensis* YIM 47672^T**	**99.17**	**12/1442**	**1491**
8K508	31	*Streptomyces griseoincarnatus* LMG 19316^T	100.00	0/1450	1491
10K101	43	*Streptomyces roseolilacinus* NBRC 12815^T	99.51	7/1441	1492
10K102	8	*Streptomyces calvus* ISP 5010^T	100.00	0/1448	1485
10K208	74	*Streptomyces roseolilacinus* NBRC 12815^T	99.51	7/1441	1489
10K209	27	*Streptomyces albidochromogenes* NBRC 101003^T	99.45	8/1448	1486
10K210	46	*Streptomyces albidochromogenes* NBRC 101003^T	99.45	8/1448	1485
10K212	74	*Streptomyces roseolilacinus* NBRC 12815^T	99.51	7/1441	1493
10K305	13	*Streptomyces albidochromogenes* NBRC 101003^T	99.45	8/1448	1485
10K307	62	*Streptomyces atrovirens* NRRL B-16357^T	99.31	10/1448	1487
10K402	19	*Streptomyces albidochromogenes* NBRC 101003^T	99.45	8/1448	1480
10K507	41	*Streptomyces atrovirens* NRRL B-16357^T	100.00	0/1449	1491
10K514	41	*Streptomyces asenjonii* KNN 35.1b^T	99.86	2/1382	1488

10K516	78	*Streptomyces cacaoi* subsp. *cacaoi* NRRL B-1220^{T}	99.93	1/1451	1491
11K101	50	*Streptomyces atrovirens* NRRL B-16357^{T}	99.93	1/1449	1486
11K402	48	*Streptomyces albidochromogenes* NBRC 101003^{T}	99.45	8/1448	1490
11K502	9	*Streptomyces marokkonensis* Ap1^{T}	99.38	9/1449	1489
12K107	65	*Streptomyces roseolilacinus* NBRC 12815^{T}	99.51	7/1441	1491
12K109	60	*Streptomyces atrovirens* NRRL B-16357^{T}	99.93	1/1449	1487
12K203	6	*Streptomyces roseolilacinus* NBRC 12815^{T}	99.51	7/1441	1492
12K302	19	*Streptomyces canarius* NBRC 13431^{T}	99.52	7/1447	1488
12K506	2	*Streptomyces rochei* NRRL B-2410^{T}	100.00	0/1448	1490
13K103	50	*Streptomyces calvus* ISP 5010^{T}	100.00	0/1448	1490
**13K105**	55	***Streptomyces gelaticus* NRRL B-2928^{T}**	**98.83**	**17/1448**	**1486**
13K109	50	*Streptomyces albogriseolus* NRRL B-1305^{T}	99.59	6/1449	1487
**13K204**	99	***Streptomyces badius* NRRL B-2567^{T}**	**98.83**	**17/1448**	**1490**
**13K301^{T}**	15	***Streptomyces variegatus* NRRL B-16380^{T}**	**98.90**	**16/1448**	**1489**
13K403	53	*Streptomyces bellus* ISP 5185^{T}	99.79	3/1414	1494
14K201	41	*Streptomyces roseolilacinus* NBRC 12815^{T}	99.51	7/1441	1490
14K203	41	*Streptomyces roseolilacinus* NBRC 12815^{T}	99.51	7/1441	1492
14K501	19	*Streptomyces canarius* NBRC 13431^{T}	99.52	7/1447	1489
15K302	68	*Streptomyces fragilis* NRRL 2424^{T}	99.93	1/1447	1489
15K408	48	*Streptomyces roseolilacinus* NBRC 12815^{T}	99.58	6/1441	1492
16K102	50	*Streptomyces calvus* ISP 5010^{T}	100.00	0/1448	1487
**16K105**	108	***Streptomyces manganisoli* MK44^{T}**	**99.22**	**11/1405**	**1492**
16K107	65	*Streptomyces roseolilacinus* NBRC 12815^{T}	99.51	7/1441	1491
16K205	48	*Streptomyces albidochromogenes* NBRC 101003^{T}	99.45	8/1448	1485
16K207	50	*Streptomyces calvus* ISP 5010^{T}	100.00	0/1448	1486
16K210	64	*Streptomyces calvus* ISP 5010^{T}	100.00	0/1448	1488
16K211	8	*Streptomyces calvus* ISP 5010^{T}	100.00	0/1448	1487
16K303	19	*Streptomyces canarius* NBRC 13431^{T}	99.52	7/1447	1485
16K306	87	*Streptomyces luteus* TRM 45540^{T}	100.00	0/1450	1491
**16K311**	51	***Streptomyces gelaticus* NRRL B-2928^{T}**	**98.83**	**17/1448**	**1488**
**16K401**	53	***Streptomyces fragilis* NRRL 2424^{T}**	**99.03**	**14/1447**	**1482**
16K501	6	*Streptomyces marokkonensis* Ap1^{T}	99.86	2/1449	1488
7K506	125	*Bacillus galliciensis* BFLP-1^{T}	99.46	8/1472	1514
13K307	129	*Lysinibacillus macroides* DSM 54^{T}	100.00	0/1474	1474
1K502	132	*Methylobacterium oryzae* CBMB20^{T}	100.00	0/1403	1435
5K112	130	*Methylobacterium oryzae* CBMB20^{T}	99.93	1/1405	1443
5K207	130	*Methylobacterium oryzae* CBMB20^{T}	100.00	0/1405	1446
10K302	126	*Methylobacterium oryzae* CBMB20^{T}	100.00	0/1405	1446
5K501	124	*Myxococcus virescens* NBRC 100334^{T}	100.00	0/1460	1494

7K510	129	*Paenibacillus lautus* NBRC 15380^T	98.71	19/1477	1522
KC615	128	*Shimazuella kribbensis* KCTC 9933^T	98.18	26/1426	1466
5K212	131	*Sphingomonas zeae* JM-791^T	99.50	7/1393	1455
3K102	127	*Stenotrophomonas rhizophila* DSM 14405^T	99.73	1/1469	1510

Table S4. Classification of isolates in colour-groups based on the distribution of reference strains identified in the 16S rRNA gene sequence analyses. Isolates in bold are shown with their nearest phylogenetic neighbours, isolates showing 16S rRNA gene sequence similarities at or below 99.3% are putatively novel species. (n = 459 isolates).

A. Multimembered colour-groups

1.	**5K221** (M*icromonospora zamorensis* DSM 45600^{T}; 100.00 %), KC914
2.	**4K405** (*Streptomyces rochei* NRRL B-2410^{T}; 100.00 %), **5K103** (*Streptomyces coeruleofuscus* NBRC 12757^{T}; 99.17 %), **12K506** (*Streptomyces rochei* NRRL B-2410^{T}; 100.00 %), KC337, KC503
3.	6K511, 7K524, **8K103** (*Micromonospora costi* CS1-12^{T}; 99.65 %), **KC202** (*Micromonospora costi* CS1-12^{T}; 99.65 %), **KC605** (*Micromonospora costi* CS1-12^{T}; all 99.65 %), KC703
4.	***5K110** (*Microbacterium testaceum* DSM 20166^{T}; 99.17 %)**,** **5K208** (*Plantactinospora veratri* NEAU-FHS4^{T}; 99.50 %), 5K219, 7K509, ******Micromonospora deserti*** **13K206^{T}** (*Micromonospora nigra* DSM 43818^{T}; 98.54 %), **KC729** (*Plantactinospora veratri* NEAU-FHS4^{T}; 99.51 %), 10K201, **KC705** (*Plantactinospora veratri* NEAU-FHS4^{T}; 99.51 %), **KC725** (*Plantactinospora veratri* NEAU-FHS4^{T}; 99.51 %), **KC728** (*Plantactinospora veratri* NEAU-FHS4^{T}; 99.44 %), 8K209
5.	5K546, **6K504** (*Nocardia flavorosea* NBRC 108225^{T}; 99.44 %)**,** **6K519** (*Nocardia flavorosea* NBRC 108225^{T}; 99.72 %), **7K528 (***Nocardia carnea* NBRC 14403^{T}; 99.72 %)**,** KC16
6.	4K201, **5K505** (*Streptomyces marokkonensis* Ap1^{T}; 99.86 %), 5K527, **8K205** (*Streptomyces roseolilacinus* NBRC 12815^{T} %), 99.51, **12K203** (*Streptomyces roseolilacinus* NBRC 12815^{T}; 99.51 %), 16K206, **16K501** (*Streptomyces marokkonensis* Ap1^{T}; 99.86 %)
7.	12K104, **KC916** (*Nocardia carnea* NBRC 14403^{T}; 99.58 %)
8.	**5K109** (*Streptomyces calvus* ISP 5010^{T}; 100.00 %), 6K301, 7K302, **10K102** (*Streptomyces calvus* ISP 5010^{T}; 100.00 %), **16K211** (*Streptomyces calvus* ISP 5010^{T}; 100.00 %)
9.	5K119, **5K135** (*Streptomyces calvus* ISP 5010^{T}; 99.86 %), 5K315, **6K525** (*Streptomyces calvus* ISP 5010^{T}; 100.00 %), **11K502** (*Streptomyces marokkonensis* Ap1^{T}; 99.38 %), **KC319** (*Streptomyces ambofaciens* ATCC 23877^{T}; 99.59 %), KC521
10.	5K554, **15K303** (*Micromonospora saelicesensis* Lupac 09^{T}; 99.72 %)**,** **16K405** (*Micromonospora vinacea* GUI63^{T}; 99.72 %)
11.	**3K201** (*Streptomyces roseolilacinus* NBRC 12815^{T}; 99.51 %), 10K202
12.	**6K501** (*Saccharomonospora azurea* NA-128^{T}; 100.00 %)**,** 6K510
13.	7K103, **10K305** (*Streptomyces albidochromogenes* NBRC 101003^{T}; 99.45 %)
14.	**5K417** (*Streptomyces nitrosporeus* NBRC 3362^{T}; 99.58 %), 6K402, **7K304** (*Streptomyces nitrosporeus* NBRC 3362^{T}; 99.58 %)
15.	******Streptomyces cahuitamycinicus*** **13K301^{T}** (*Streptomyces variegatus* NRRL B-16380^{T}; 98.90 %), 13K303
16.	**4K205** (*Micromonospora costi* CS1-12^{T}; 99.65 %), 8K101, 13K101, 13K108, **13K111** (*Micromonospora costi* CS1-12^{T}; 99.65 %), **13K203** (*Micromonospora costi* CS1-12^{T}; 99.65 %), **15K101** (*Micromonospora costi* CS1-12^{T}; 99.65 %)
17.	**5K316** (*Streptomyces coeruleofuscus* NBRC 12757^{T}; 99.17 %), 10K301
18.	***KC213** (*Micromonospora halophytica* DSM 43171^{T}; 99.17 %), KC704, KC718
19.	7K303, 8K305, **10K402** (*Streptomyces albidochromogenes* NBRC 101003^{T}; 99.45 %), **12K302** (*Streptomyces canarius* NBRC 13431^{T}; 99.52 %), 14K303, 14K304, **14K501** (*Streptomyces canarius* NBRC 13431^{T}; 99.52 %), **16K303** (*Streptomyces canarius* NBRC 13431^{T}; 99.52 %), 16K310

20.	**5K118** (*Micromonospora saelicesensis* Lupac 09^{T}; 99.79 %)**,** **7K104** (*Micromonospora saelicesensis* Lupac 09^{T}; 99.79 %)**,** 12K404, 13K202, **13K208** (*Micromonospora saelicesensis* Lupac 09^{T}; 99.79 %)**,** **15K103** (*Micromonospora saelicesensis* Lupac 09^{T}; 99.79 %)
21.	3K301, **6K103** (*Streptomyces spinoverrucosus* NBRC 14228^{T}; 99.93 %), **7K305** (*Streptomyces spinoverrucosus* NBRC 14228^{T}; 99.65 %)
22.	12K503, **KC708** (*Micromonospora halophytica* DSM 43171^{T}; 99.24 %)
23.	10K213, 12K502, 15K102, **KC204** (*Micromonospora halophytica* DSM 43171^{T}; 99.24 %), **KC205 (***Micromonospora halophytica* DSM 43171^{T}; 99.24 %), **KC215** (*Micromonospora palomenae* NEAU-CX1^{T}; 99.10 %), ***KC606** (*Micromonospora costi* CS1-12^{T} 99.03 %), **KC707** (*Micromonospora halophytica* DSM 43171^{T}; 99.24 %), ***KC721** (*Micromonospora palomenae* NEAU-CX1^{T}; 99.10 %), ***KC723** (*Micromonospora inositola* DSM 43819^{T}; 99.03 %)
24.	******Nonomuraea aridisoli*** **KC333^{T}** (*Nonomuraea maritima* FXJ7.203^{T}; 98.93 %), KC514, KC517
25.	***15K316** (*Micromonospora avicenniae* DSM 45758^{T}; 99.37 %)**,** **16K103** (*Micromonospora avicenniae* DSM 45758^{T}; 99.37 %), KC711, KC904
26.	KC203, KC309, KC341, KC414, KC508, KC519, **KC713** (*Nonomuraea salmonea* DSM 43678^{T}; 98.12 %), **KC730** (*Nonomuraea salmonea* DSM 43678^{T}; 98.12 %), **KC929** (*Nonomuraea salmonea* DSM 43678^{T}; 98.12 %)
27.	7K101, **7K111** (*Streptomyces calvus* ISP 5010^{T}; 99.93 %), KC722, **8K206** (*Streptomyces calvus* ISP 5010^{T}; 100.00%), **10K209** (*Streptomyces albidochromogenes* NBRC 101003^{T}; 99.45 %),
28.	**3K203** (*Streptomyces atrovirens* NRRL B-16357^{T}; 99.93 %), **7K201** (*Streptomyces calvus* ISP 5010^{T}; 100.00 %), 7K203, KC731
29.	**5K101** (*Streptomyces xantholiticus* NBRC 13354^{T}; 99.23 %), KC214, KC314
30.	3K103, **KC416** (*Streptomyces kurssanovii* NBRC 13192^{T}; 100.00 %), **KC506** (*Streptomyces ambofaciens* ATCC 23877^{T}; 99.59 %)
31.	**7K529** (*Streptomyces leeuwenhoekii* C34^{T}; 99.86 %), **8K508** (*Streptomyces griseoincarnatus* LMG 19316^{T}; 100.00 %), **KC340** (*Streptomyces thinghirensis* DSM 41919^{T}; 99.38 %), KC402, **KC501** (*Streptomyces thinghirensis* DSM 41919^{T}; 99.10 %), KC513, KC912, **KC918** (*Streptomyces thinghirensis* DSM 41919^{T}; 99.38 %), KC923
32.	7K403, **KC421** (*Streptomyces kurssanovii* NBRC 13192^{T}; 99.93 %)
33.	**6K521** (*Actinomadura deserti* BMP B8004^{T}; 99.44 %)**,** 7K503, 8K511, **10K511** (*Actinomadura sediminis* YIM M 10931^{T}; 100.00 %)**,** **10K521** (*Actinomadura deserti* BMP B8004^{T}; 99.38 %)
34.	**4K102** (*Saccharopolyspora endophytica* YIM 61095^{T}; 99.65 %)**,** ******Saccharopolyspora aridisoli*** **16K404^{T}** (*Saccharopolyspora endophytica* YIM 61095^{T}; 99.65 %), KC312, **KC608** (*Actinomadura bangladeshensis* 3-46-b3^{T}; 99.82 %)**,** KC911
35.	12K305, 15K307, **KC11** (*Streptomyces ambofaciens* ATCC 23877^{T}; 99.59 %), **KC12** (*Streptomyces ambofaciens* ATCC 23877^{T}; 99.59 %), **KC407** (*Streptomyces ambofaciens* ATCC 23877^{T}; 99.59 %)
36.	KC338, **KC406** *(Nonomuraea salmonea* DSM 43678^{T}; 97.98 %), **KC727** (*Nonomuraea salmonea* DSM 43678^{T}; 97.98 %), **KC921** (*Nonomuraea salmonea* DSM 43678^{T}; 97.98 %)
37.	6K516, 10K526, **KC507** (*Nonomuraea candida* HMC10^{T}; 98.07 %), **KC509** (*Nonomuraea candida* HMC10^{T}; 98.05 %)
38.	5K504, **5K520** (*Streptomyces fradiae* DSM 40063^{T}; 99.72), **5K526** (*Streptomyces rubrogriseus* LMG 20318^{T}; 99.79), **7K501** (*Streptomyces rubrogriseus* LMG 20318^{T}; 100.00 %), 7K508, 7K512, **7K513** (*Streptomyces marokkonensis* Ap1^{T}; 99.38 %), 10K509, 13K502, KC321
39.	16K402, **KC209** (*Nocardia carnea* NBRC 14403^{T}; 99.58 %), **KC910** (*Nocardia carnea* NBRC 14403^{T}; 99.58 %)
40.	12K205, **16K403** (*Nocardiopsis trehalosi* NBRC 14201^{T}; 100.00 %)
41.	**4K203** (*Streptomyces roseolilacinus* NBRC 12815^{T}; 99.51 %), 5K508, **10K507** (*Streptomyces atrovirens* NRRL B-16357^{T}; 100.00 %), **10K514** (*Streptomyces asenjonii* KNN 35.1b^{T}; 99.86 %), 13K304, 13K306, **14K201** (*Streptomyces roseolilacinus* NBRC 12815^{T}; 99.51 %), **14K203** (*Streptomyces*

	roseolilacinus NBRC 12815^T; 99.51 %), 16K301
42.	**3K402** (*Nocardiopsis umidischolae* 66/93^T; 100.00 %)**,** 14K401, 15K305, **15K402** (*Nocardiopsis umidischolae* 66/93^T; 100.00 %)**,** KC701
43.	**5K125** (*Streptomyces spinoverrucosus* NBRC 14228^T; 99.72 %), **5K122** (*Streptomyces roseolilacinus* NBRC 12815^T; 99.51 %), 5K411, **8K102** (*Streptomyces roseolilacinus* NBRC 12815^T; 99.51 %), **10K101** (*Streptomyces roseolilacinus* NBRC 12815^T; 99.51 %), 10K522,
44.	**1K501** (*Nonomuraea ceibae* XMU 110^T; 98.82 %)**, 7K519** (*Nonomuraea ceibae* XMU 110^T; 99.79)**,** 15K315
45.	5K137, 5K510, **5K418** (*Pseudonocardia kunmingensis* YIM 63158^T 99.35 %), 7K521, 16K106, 16K304, 16K308, **KC304** (*Pseudonocardia adelaidensis* EUM 221^T; 99.36 %), **KC323** (*Pseudonocardia adelaidensis* EUM 221^T; 99.43 %)
46.	7K402, **10K210** (*Streptomyces albidochromogenes* NBRC 101003^T; 99.45 %)
47.	***6K520** (*Actinomadura livida* JCM 3387^T; 99.03 %), ***KC345** (*Actinomadura bangladeshensis* 3-46-b3^T; 98.41 %)
48.	10K404, **11K402** (*Streptomyces albidochromogenes* NBRC 101003^T; 99.45 %), 15K309, 15K401, **15K408** (*Streptomyces roseolilacinus* NBRC 12815^T; 99.58 %), **16K205** (*Streptomyces albidochromogenes* NBRC 101003^T; 99.45 %),
49.	**4K403B** (*Agromyces subbeticus* DSM 16689^T; 98.76 %), 5K542
50.	**5K114** (*Streptomyces calvus* ISP 5010^T; 100.00 %), 6K101, 6K522, 7K514, 10K103, **11K101** (*Streptomyces atrovirens* NRRL B-16357^T; 99.93 %), **13K103** (*Streptomyces calvus* ISP 5010^T; 100.00 %), **13K109** (*Streptomyces albogriseolus* NRRL B-1305^T; 99.59 %), 15K501, **16K102** (*Streptomyces calvus* ISP 5010^T; 100.00 %), **16K207** (*Streptomyces calvus* ISP 5010^T; 100.00 %), 16K209, KC326
51.	7K105, **16K311** (*Streptomyces gelaticus* NRRL B-2928^T; 98.83)
52.	**5K531** (*Streptomyces marokkonensis* Ap1^T; 99.65 %), **5K532** (*Streptomyces marokkonensis* Ap1^T; 99.86 %), 6K507, 6K513, 10K519
53.	**13K403** (*Streptomyces bellus* ISP 5185^T; 99.79 %), 15K404, **16K401** (*Streptomyces fragilis* NRRL 2424^T; 99.03 %)
54.	**6K502** (*Streptomyces marokkonensis* Ap1^T; 99.38 %), 8K502, 11K503
55.	**13K105** (*Streptomyces gelaticus* NRRL B-2928^T; 98.83 %), KC404
56.	5K111, **5K133** (*Pseudonocardia hierapolitana* PM2084^T; 99.93 %)**, 5K313** (*Pseudonocardia hierapolitana* PM2084^T; 100.00 %)
57.	**5K515** (*Actinomadura geliboluensis* A8036^T; 100.00 %)**, 6K523** (*Actinomadura geliboluensis* A8036^T; 100.00 %)**, 7K401** (*Actinomadura geliboluensis* A8036^T; 100.00 %), KC307, KC424
58.	***KC06** (*Actinomadura darangshiensis* DSLS-70^T; 98.52%), KC206, ***KC216** (*Actinomadura darangshiensis* DSLS-70^T; 98.80 %)**,**
59.	7K511, **KC320** (*Nocardia carnea* NBRC 14403^T; 99.58 %)
60.	10K505, **12K109** (*Streptomyces atrovirens* NRRL B-16357^T; 99.93 %), **KC903** (*Streptomyces ambofaciens* ATCC 23877^T; 99.86 %)
61.	**8K301** (*Streptomyces coeruleofuscus* NBRC 12757^T; 99.10 %), 10K304
62.	**10K307** (*Streptomyces atrovirens* NRRL B-16357^T; 99.31 %), KC920
63.	5K311, 10K303, **KC311 (***Pseudonocardia zijingensis* 6330^T; 99.58 %), **KC316** (*Pseudonocardia adelaidensis* EUM 221^T; 99.57 %*),* **KC325** (*Pseudonocardia adelaidensis* EUM 221^T; 99.57 %), KC334, **KC502** (*Pseudonocardia zijingensis* 6330^T; 99.51 %), **KC510** (*Pseudonocardia adelaidensis* EUM 221^T; 99.57 %), KC516, KC520
64.	**6K201** (*Streptomyces atrovirens* NRRL B-16357^T; 99.93 %), 7K202, 10K211, 13K201, **16K210** (*Streptomyces calvus* ISP 5010^T; 100.00 %)
65.	KC714, **12K107** (*Streptomyces roseolilacinus* NBRC 12815^T; 99.51 %), **16K107** (*Streptomyces roseolilacinus* NBRC 12815^T; 99.51 %)

66.	**7K109** (*Kribbella pittospori* PIP 158^T; 99.93 %)**,** KC328
67.	5K307, 5K309, ***Nonomuraea deserti KC310^T** (*Nonomuraea candida* HMC10^T; 98.07 %), **KC327** (*Nonomuraea candida* HMC10^T; 98.07 %), **KC518** (*Nonomuraea candida* HMC10^T; 98.07 %)
68.	5K306, **5K312** (*Streptomyces roseolilacinus* NBRC 12815^T; 99.51 %), 6K304, **7K522** (*Streptomyces luteus* TRM 45540^T; 99.66 %), 13K302, 13K305, **15K302** (*Streptomyces fragilis* NRRL 2424^T; 99.93 %), 15K310
69.	**5K551** (*Streptomyces kurssanovii* NBRC 13192^T; 99.93 %), 10K503, 10K104, **KC15** (*Streptomyces kurssanovii* NBRC 13192^T; 99.93 %), **KC410** (*Streptomyces kurssanovii* NBRC 13192^T; 99.93 %)
70.	**5K134** (*Nonomuraea maheshkhaliensis* 16-5-14^T; 98.75 %)**, 5K320** (*Nonomuraea glycinis* NEAU-BB2C19^T; 99.72 %)**, 5K322** (*Nonomuraea maheshkhaliensis* 16-5-14^T; 98.75 %)**, 7K532** (*Nonomuraea glycinis* NEAU-BB2C19^T; 99.65 %)**,** KC322
71.	***Jiangella asiatica 5K138^T** (*Jiangella alba* DSM 45237^T; 99.45 %)**,** 5K539, 7K112, 7K526, ***7K534** (*Actinomadura livida* JCM 3387^T; 99.31 %)
72.	5K522, **5K544** (*Micromonospora citrea* DSM 43903^T; 99.51%), 7K518, **7K530** (*Micromonospora citrea* DSM 43903^T; 99.51 %)**, 8K503** (*Micromonospora citrea* DSM 43903^T; 99.51 %), 10K508, **10K527** (*Micromonospora citrea* DSM 43903^T; 99.51 %), **12K108** (*Micromonospora citrea* DSM 43903^T; 99.51 %)
73.	5K108, **5K131** (*Streptomyces thinghirensis* DSM 41919^T; 99.36 %), **5K132** (*Streptomyces thinghirensis* DSM 41919^T; 99.36
74.	5K201, **10K208** (*Streptomyces roseolilacinus* NBRC 12815^T; 99.51 %), **10K212** (*Streptomyces roseolilacinus* NBRC 12815^T; 99.51 %)
75.	5K536, 5K553, **6K505** (*Nocardia carnea* NBRC 14403^T; 100.00 %), **7K517** (*Nocardia carnea* NBRC 14403^T; 99.93 %), **7K520** (*Nocardia carnea* NBRC 14403^T; 99.72 %)
76.	4K207, **5K222** (*Nocardiopsis dassonvillei* subsp. *dassonvillei* DSM 43111^T; 100.00 %)**,** 5K419, **5K521** (*Nocardiopsis dassonvillei* subsp. *dassonvillei* DSM 43111^T; 100.00 %), **10K510** (*Nocardiopsis dassonvillei* subsp. *dassonvillei* DSM 43111^T; 100.00 %),
77.	**5K506** (*Saccharopolyspora shandongensis* 88^T; 99.44 %), 5K523, 5K545, 5K547, **6K514** (*Saccharopolyspora shandongensis* 88^T; 99.58 %)**, *Saccharopolyspora elongata 7K502^T** (*Saccharopolyspora shandongensis* 88^T; 99.44 %)
78.	5K552, **10K516** (*Streptomyces cacaoi* subsp. *cacaoi* NRRL B-1220^T; 99.93 %)
79.	**KC301** (*Pseudonocardia cypriaca* KT2142^T; 99.51 %), **KC504** (*Pseudonocardia cypriaca* KT2142^T; 99.51 %), KC515
80.	**4K104** (*Nonomuraea glycinis* NEAU-BB2C19^T; 99.79 %), 5K127, 5K120, **7K110** (*Nonomuraea glycinis* NEAU-BB2C19^T; 99.72)
81.	10K512, **KC403** (*Streptomyces ambofaciens* ATCC 23877^T; 99.59 %), **KC907** (*Streptomyces ambofaciens* ATCC 23877^T; 99.59 %)
82.	3K302, 6K302, ***Nonomuraea longispora KC201^T** (*Nonomuraea salmonea* DSM 43678^T; 97.98 %), **KC306** (*Nonomuraea salmonea* DSM 43678^T; 97.98 %), KC331, **KC332** (*Nonomuraea salmonea* DSM 43678^T; 97.98 %), KC505, **KC706** (*Nonomuraea salmonea* DSM 43678^T; 97.98 %), **KC924** (*Nonomuraea salmonea* DSM 43678^T; 97.98 %)
83.	**1K503** (*Jiangella rhizosphaerae* NEAU-YY265^T; 99.72 %), **5K116** (*Jiangella rhizosphaerae* NEAU-YY265^T; 99.72 %)**,** 5K126, 5K310, ***Jiangella aurantiaca 8K307^T** (*Jiangella rhizosphaerae* NEAU-YY265^T; 99.72 %)
84.	KC329, **KC512** (*Nonomuraea maritima* FXJ7.203^T; 98.93 %)
85.	5K204, 6K401, **12K402** (*Micromonospora fluostatini* PWB-003^T; 99.51), ***KC207** (*Micromonospora fluostatini* PWB-003^T; 99.44 %), **KC208** (*Micromonospora fluostatini* PWB-003^T; 99.44 %)
86.	5K105, 5K517, 5K528, **6K515** (*Actinomadura cremea* JCM 3308^T; 99.93 %), **7K527** (*Actinomadura cremea* JCM 3308^T; 99.93 %)**, 7K525** (*Actinomadura cremea* JCM 3308^T; 99.86 %), 7K531, **7K537** (*Actinomadura cremea* JCM 3308^T; 99.93 %), **10K502** (*Actinomadura cremea* JCM 3308^T; 99.93 %), **10K504** (*Actinomadura cremea* JCM 3308^T; 99.93 %)

87.	**7K301** (*Streptomyces luteus* TRM 45540^T; 100.00 %), 10K306, 14K301, **16K306** (*Streptomyces luteus* TRM 45540^T; 100.00 %), **KC18** (*Streptomyces luteus* TRM 45540^T; 99.93 %)
88.	**5K502** (*Actinocorallia aurantiaca* JCM 8201^T; 99.16 %), **5K516** (*Actinocorallia aurantiaca* JCM 8201^T; 99.51 %), **5K550** (*Actinocorallia libanotica* IFO 14095^T; 99.07 %)

B. Single membered colour-groups

89.	**KC426** (*Streptomyces kurssanovii* NBRC 13192^T; 99.93 %)
90.	**3K401** (*Streptomyces krungchingensis* KC-035^T; 98.48 %)
91.	**15K301** (*Nonomuraea turkmeniaca* DSM 43926^T; 98.73 %)
92.	**KC103** (*Saccharopolyspora erythraea* NRRL 2338^T; 100.00 %)
93.	**KC408** (*Saccharopolyspora erythraea* NRRL 2338^T; 99.93 %)
94.	***Nonomuraea longispora* KC401** (*Nonomuraea salmonea* DSM 43678^T; 98.12 %)
95.	**7K523** (*Nonomuraea harbinensis* NEAU-yn31^T; 99.65 %)
96.	**KC601** (*Plantactinospora veratri* NEAU-FHS4^T; 98.68 %)
97.	**KC930** (*Streptomyces thinghirensis* DSM 41919T; 99.31 %)
98.	**5K209** (*Streptomyces barkulensis* RC 1831^T; 98.65 %)
99.	**13K204** (*Streptomyces badius* NRRL B-2567^T; 98.83 %)
100.	**5K555** (*Actinomadura deserti* BMP B8004^T; 99.44 %)
101.	***Saccharopolyspora karakumensis* 5K548^T** (*Saccharopolyspora pathumthaniensis* S582^T; 99.79 %)
102.	***Nonomuraea mesophila* 6K102^T** (*Nonomuraea salmonea* DSM 43678^T; 98.33 %)
103.	**7K533** (*Actinomadura cremea* JCM 3308^T; 99.09 %)
104.	**16K305** (*Micromonospora fiedleri* MG-37^T; 99.57 %)
105.	**15K203** (*Nonomuraea polychroma* DSM 43925^T; 98.41 %)
106.	**4K202** (*Nonomuraea flavida* ex1^T; 99.51 %)
107.	**4K403A** (*Nonomuraea glycinis* NEAU-BB2C19^T; 99.65 %)
108.	**16K105** (*Streptomyces manganisoli* MK44^T; 99.22 %)
109.	**KC101** (*Saccharopolyspora erythraea* NRRL 2338^T; 99.93 %)
110.	***Saccharopolyspora terrae* 16K309^T** (*Saccharopolyspora pathumthaniensis* S582^T; 99.65 %)
111.	***8K308** (*Streptomyces hainanensis* YIM 47672^T; 99.17 %)
112.	***7K507** (*Actinomadura bangladeshensis* 3-46-b3^T; 98.89 %)
113.	**8K306** (*Nonomuraea jabiensis* A4036^T; 98.96 %)
114.	***Spongiactinospora gelatinilytica* 7K107^T** (*Spongiactinospora rosea* LHW63015^T; 98.69 %)

115.	**7K515** (*Actinomadura darangshiensis* DSLS-70^{T}; 98.52 %)
116.	**5K409** (*Micromonospora citrea* DSM 43903^{T}; 99.79 %)
117.	**4K301** (*Streptomyces manganisoli* MK44^{T}; 99.29 %)
118.	***Kribbella turkmenica* 16K104^{T}** (*Kribbella albertanoniae* BC640^{T}; 99.17 %)
119.	***Jiangella ureilytica* KC603^{T}** (*Jiangella mangrovi* 3SM4-07^{T}; 99.44 %)
120.	***Nonomuraea diastatica* KC712^{T}** (*Nonomuraea candida* HMC10^{T}; 98.07 %)
121.	**5K524** (*Streptomyces xantholiticus* NBRC 13354^{T}; 99.37 %)
122.	**7K504** (*Micromonospora aurantiaca* ATCC 27029^{T}; 100.00 %)
123.	***Nocardioides turkmenicus* KC13^{T}** (*Nocardioides luteus* KCTC 9575^{T}; 99.30)
**Non-actinomycete isolates**	
124.	5K303, **5K501** (*Myxococcus virescens* NBRC 100334^{T}; 100.00 %)
125.	**7K506 (***Bacillus galliciensis* BFLP-1^{T}; 99.46 &)
126.	5K305, 5K324, **10K302 (***Methylobacterium oryzae* CBMB20^{T}; 100.00 %)
127.	**3K102 (***Stenotrophomonas rhizophila* DSM 14405^{T}; 99.73 %)
128.	***Shimazuella alba* KC615^{T}** (*Shimazuella kribbensis* KCTC 9933^{T}; 98.18 %)
129.	**7K510 (***Paenibacillus lautus* NBRC 15380^{T}; 98.71 %), 12K103, **13K307 (***Lysinibacillus macroides* DSM 54^{T}; 100.00 %)
130.	**5K112 (***Methylobacterium oryzae* CBMB20^{T}; 99.93 %), **5K207 (***Methylobacterium oryzae* CBMB20^{T}; 100.00 %), 5K301
131.	3K202, **5K212 (***Sphingomonas zeae* JM-791^{T}; 99.50 %)
132.	**1K502 (***Methylobacterium oryzae* CBMB20^{T}; 100.00 %)

Table S5. Genes associated with phosphate metabolism, tryptophan synthesis and the production of degradative enzymes detected in the genomes of selected isolates. In total, 32 isolates were screened.

Function	KC06	KC216	KC345	6K520	7K507	7K534	KC603T	5K138T	8K307T	16K104T	5K110	KC207	KC213	KC606	KC721	KC723	13K206T	15K316	KC13T	KC201T	KC310T	KC333T	KC401	KC712T	6K102T	5K548T	7K502T	16K309T	16K404T	7K107T	8K308	13K301T
**Phosphate metabolism**																																
Alkaline phosphatase (EC 3.1.3.1)	-	-	-	-	-	-	+	+	-	-	+	+	+	+	-	-	+	+	+	-	+	+	-	+	-	-	-	-	-	-	+	-
Alkaline phosphatase like protein	-	-	-	-	-	-	-	-	-	-	-	+	+	+	+	+	+	+	-	-	-	-	-	-	-	-	-	-	-	-	-	-
Exopolyphosphatase (EC 3.6.1.11)	+	+	+	+	+	+	+	+	+	+	+	+	+	+	+	+	+	+	+	+	+	+	+	+	+	+	+	+	+	+	+	+
Inorganic pyrophosphatase ppa (EC 3.6.1.1)	+	+	+	+	+	+	+	+	+	+	+	+	+	+	+	+	+	+	+	+	+	+	+	+	+	+	+	+	+	+	+	+
NAD(P) transhydrogenase alpha subunit (EC 1.6.1.2) Tha	+	+	-	+	-	-	+	+	+	+	-	-	-	-	-	-	-	-	-	-	-	-	-	-	-	+	+	-	-	-	-	-
NAD(P) transhydrogenase beta subunit (EC 1.6.1.2) THb	+	+	-	+	-	-	+	+	+	+	-	-	-	-	-	-	-	-	+	-	-	-	-	-	-	+	+	-	-	-	-	+
Phosphate ABC transporter, periplasmic phosphate-binding protein PstS (TC 3.A.1.7.1)	+	+	+	+	+	+	+	+	+	+	+	+	+	+	+	+	+	+	+	+	+	+	+	+	+	+	+	+	+	+	+	+
Phosphate regulon sensor protein PhoR (SphS) (EC 2.7.13.3)	+	+	+	+	+	+	+	+	+	+	+	+	+	+	+	+	+	+	+	+	+	+	+	+	+	+	+	+	+	+	+	+
Phosphate regulon transcriptional regulatory protein PhoB (SphR)	+	+	+	+	+	+	+	+	+	+	+	+	+	+	+	+	+	+	+	+	+	+	+	+	+	+	+	+	+	+	+	+
Phosphate transport ATP-binding protein PstB (TC 3.A.1.7.1)	+	+	+	+	+	+	+	+	+	+	+	+	+	+	+	+	+	+	+	+	+	+	+	+	+	+	+	+	+	+	+	+
Phosphate transport regulator (distant homolog of PhoU)	+	+	+	+	+	+	+	+	+	+	-	+	+	+	+	+	+	+	+	+	+	+	+	+	+	-	-	-	-	+	+	+
Phosphate transport system permease protein PstA (TC 3.A.1.7.1)	+	+	+	+	+	+	+	+	+	+	+	+	+	+	+	+	+	+	+	+	+	+	+	+	+	+	+	+	+	+	+	+
Phosphate transport system permease protein PstC (TC 3.A.1.7.1)	+	+	+	+	+	+	+	+	+	+	+	+	+	+	+	+	+	+	+	+	+	+	+	+	+	+	+	+	+	+	+	+
Phosphate transport system regulatory protein PhoU	+	+	+	+	+	+	+	+	+	+	+	+	+	+	+	+	+	+	+	+	+	+	+	+	+	+	+	+	+	+	+	+
Polyphosphate kinase (EC 2.7.4.1)	+	+	+	+	+	+	+	+	+	+	+	+	+	+	+	+	+	+	+	+	+	+	+	+	+	+	+	+	+	+	+	+
Predicted ATPase related to phosphate starvation-inducible protein PhoH	+	+	+	+	+	+	+	+	+	+	+	+	+	+	+	+	+	+	+	+	+	+	+	+	+	+	+	+	+	+	+	+
Probable low-affinity inorganic phosphate transporter	+	+	+	+	+	+	+	+	+	+	+	+	+	+	+	+	+	+	+	+	+	+	+	+	+	-	-	-	-	+	+	+
Pyrophosphate-energized proton pump (EC 3.6.1.1)	+	+	+	+	+	+	+	+	+	+	-	+	+	+	+	+	+	+	+	+	+	+	+	+	+	+	+	+	+	+	+	+
Secreted alkaline phosphatase	+	+	+	+	+	+	+	+	+	+	+	+	+	+	+	+	+	+	+	+	+	+	+	+	+	+	+	+	+	+	+	+
Soluble pyridine nucleotide transhydrogenase (EC 1.6.1.1)	+	+	+	+	+	+	+	-	+	-	-	+	-	-	-	-	-	-	-	-	+	-	+	+	+	-	-	-	-	-	-	-
**Tryptophan synthesis**																																
Acting phosphoribosylanthranilate	+	+	+	+	+	+	+	+	+	+	+	+	+	+	+	+	+	+	+	+	+	+	+	+	+	+	+	+	+	-	+	+

isomerase (EC 5.3.1.24)																																
Aminodeoxychorismate lyase (EC 4.1.3.38)	+	+	+	+	+	+	+	+	+	+	+	+	+	+	+	+	+	+	+	+	+	+	+	+	+	+	+	+	+	-	+	+
Anthranilate phosphoribosyltransferase (EC 2.4.2.18)	+	+	+	+	+	+	+	+	+	+	+	+	+	+	+	+	+	+	+	+	+	+	+	+	+	+	+	+	+	-	+	+
Anthranilate synthase, amidotransferase component (EC 4.1.3.27)	+	+	+	+	+	+	+	+	+	+	+	+	+	+	+	+	+	+	-	+	+	+	+	+	+	+	+	+	+	-	-	-
Anthranilate synthase, aminase component (EC 4.1.3.27)	+	+	+	+	+	+	+	+	+	+	-	+	+	+	+	+	+	+	+	+	+	+	+	+	+	+	+	+	+	-	+	+
Aromatic-L-amino-acid decarboxylase (EC 4.1.1.28)	-	-	-	-	-	-	+	+	+	-	+	+	-	-	-	-	-	-	-	-	-	+	-	-	-	+	+	+	+	-	-	-
Indole-3-glycerol phosphate synthase (EC 4.1.1.48)	+	+	+	+	+	+	+	+	+	+	+	+	+	+	+	+	+	+	+	+	+	+	+	+	+	+	+	+	+	-	+	+
Monoamine oxidase (1.4.3.4)	-	-	-	-	-	-	+	+	+	-	+	+	-	-	-	-	-	-	-	+	+	-	+	+	+	+	+	+	+	-	-	+
Para-aminobenzoate synthase, amidotransferase component (EC 2.6.1.85)	+	+	-	+	+	+	+	+	+	+	+	+	+	+	+	+	+	+	+	+	+	+	+	+	+	+	+	+	+	-	+	+
Para-aminobenzoate synthase, aminase component (EC 2.6.1.85)	+	+	-	+	+	+	+	+	+	+	+	+	+	+	+	+	+	+	+	+	+	+	+	+	+	+	+	+	+	-	+	+
Phosphoribosylanthranilate isomerase (EC 5.3.1.24)	-	-	-	-	-	-	-	-	-	-	-	-	-	-	-	-	-	-	-	+	+	+	+	+	+	-	-	-	-	-	-	-
Tryptophan synthase alpha chain (EC 4.2.1.20)	+	+	+	+	+	+	+	+	+	+	+	+	+	+	+	+	+	+	+	+	+	+	+	+	+	+	+	+	+	-	+	+
Tryptophan synthase beta chain (EC 4.2.1.20)	+	+	+	+	+	+	+	+	+	+	+	+	+	+	+	+	+	+	+	+	+	+	+	+	+	+	+	+	+	-	+	+
Tryptophan-associated membrane protein	+	+	+	+	+	+	+	-	+	+	+	+	+	+	+	+	+	+	+	-	-	-	-	-	-	-	+	-	-	-	+	+
**Degradative enzymes**																																
Alpha-amylase (EC 3.2.1.1)	+	+	+	+	+	+	+	+	+	+	+	+	-	-	-	-	+	-	-	+	+	+	+	+	+	+	+	+	+	+	+	+
Cellulase (EC 3.2.1.4)	-	-	-	+	-	+	-	-	-	-	-	+	-	-	-	+	-	-	-	-	-	-	-	-	-	-	-	-	-	-	+	-
Chitinase (EC 3.2.1.14)	+	+	+	+	+	+	+	+	-	+	-	+	+	+	+	+	+	+	+	+	+	+	+	+	+	+	+	+	+	+	+	+
Chitin binding protein	+	+	+	+	+	+	-	-	-	+	-	-	-	-	-	-	+	+	+	+	+	+	+	+	+	+	+	+	+	+	+	+
Cutinase	-	-	-	-	-	-	-	-	-	-	-	-	+	-	+	-	-	-	-	-	-	-	-	-	-	-	-	-	-	-	-	+
Endo-1,4-ß-xylanase (EC 3.2.1.8)	-	-	-	+	+	+	-	-	+	+	+	+	+	+	+	+	+	-	+	+	+	+	+	+	+	+	+	+	+	-	+	+
Endoglucanase precursor cellulase (EC 3.2.1.4)	+	-	-	+	+	-	-	-	-	+	-	+	-	-	-	-	+	-	-	-	-	-	-	-	-	-	+	-	-	+	+	-
Esterase/lipase	+	+	+	+	+	+	+	+	+	+	-	+	+	+	+	+	+	+	-	+	+	+	+	+	+	+	+	+	+	+	+	+
Glucoamylase (EC 3.2.1.3)	+	-	+	+	+	+	+	+	+	+	+	+	-	+	-	-	+	+	-	+	+	+	+	+	+	+	+	+	+	+	+	+
Laccase (EC 1.10.3.2)	-	-	-	-	-	-	-	-	-	+	-	+	-	-	-	-	-	-	+	+	+	+	+	+	+	-	+	-	-	+	+	-
Mannan endo-1,4-beta-mannosidase (EC 3.2.1.78)	-	-	-	-	-	-	-	-	-	-	-	+	+	+	+	+	+	-	-	+	+	-	+	+	+	-	-	-	-	-	+	+
Pectate lyase (EC 4.2.2.2)	-	-	-	+	-	+	-	+	+	+	-	+	+	+	+	+	+	-	-	+	+	+	+	+	+	-	-	-	+	-	+	+
Pectinesterase (EC 3.1.1.11)	-	-	-	-	-	-	-	-	-	-	-	-	-	+	-	-	-	-	-	-	-	-	-	-	-	-	-	-	-	-	+	-
Phosphoglucomutase (EC 5.4.2.2)	-	-	-	-	-	-	+	+	+	+	+	+	+	+	+	+	+	+	+	+	+	+	+	+	+	-	-	-	-	+	-	+
Secreted cellulase (EC 3.2.1.14)	-	-	-	-	-	-	-	-	-	-	-	+	+	-	+	-	-	-	-	-	-	-	-	-	-	-	-	-	-	-	-	-

Secreted pectinesterase (EC 3.1.1.11)	-	-	-	-	-	-	-	-	-	-	-	-	-	-	-	-	-	-	-	-	-	-	-	-	-	-	-	-	-	-	-	+
ß-glucosidase (EC 3.2.1.21)	+	+	+	+	+	+	+	+	+	+	+	+	+	+	+	+	+	+	+	+	+	+	+	+	+	+	+	+	+	-	+	+
Trehalase (EC 3.2.1.28)	+	+	+	+	+	+	-	-	-	-	-	+	+	+	+	+	+	+	-	-	-	+	-	-	-	-	-	-	-	+	-	+
Type III effector HrpW, hairpin with pectate lyase domain	-	-	-	-	-	-	-	-	-	-	-	-	-	+	-	-	-	-	-	-	-	-	-	-	-	-	-	-	-	-	-	+
Xylan 1,4-beta-xylosidase (EC 3.2.1.37)	-	-	+	+	+	+	+	+	+	+	+	+	+	-	+	+	+	-	-	+	+	+	+	+	+	-	-	-	-	-	+	+

Table S6. Genes associated with siderophore production detected in the genomes of selected isolates. In total, 32 isolates were screened.

Function	KC06	KC216	KC345	6K520	7K507	7K534	KC603^T	5K138^T	8K307^T	16K104^T	5K110	KC207	KC213	KC606	KC721	KC723	13K206^T	15K316	KC13^T	KC201^T	KC310^T	KC333^T	KC401	KC712^T	6K102^T	5K548^T	7K502^T	16K309^T	16K404^T	7K107^T	8K308	13K301^T
**Iron acquisition**																																
Ferric iron ABC transporter, ATP-binding protein	-	-	-	+	-	+	+	-	+	+	-	+	-	+	+	+	+	-	+	-	-	-	-	-	-	-	-	-	-	-	-	-
Ferric iron ABC transporter, iron-binding protein	-	-	-	+	-	+	+	-	+	+	-	+	-	+	+	+	+	-	+	-	-	-	-	-	-	-	-	-	-	-	-	-
Ferric iron ABC transporter, permease protein	-	-	-	+	-	+	+	-	+	+	-	+	-	+	+	+	+	-	+	-	-	-	-	-	-	-	-	-	-	-	-	-
**Siderophore Desferrioxamine E**																																
Desferrioxamine E biosynthesis protein DesA	-	-	-	-	-	-	-	-	-	-	-	-	-	-	-	+	-	+	-	-	-	-	-	-	-	-	-	-	-	-	-	+
Desferrioxamine E biosynthesis protein DesB	-	-	-	-	-	-	-	-	-	-	-	-	-	-	-	+	-	+	-	-	-	-	-	-	-	-	-	-	-	-	-	+
Desferrioxamine E biosynthesis protein DesC	-	-	-	-	-	-	-	-	-	-	-	-	-	-	-	+	-	+	-	-	-	-	-	-	-	-	-	-	-	-	+	+
Desferrioxamine E biosynthesis protein DesD	-	-	-	-	-	-	-	-	-	+	-	-	-	-	-	+	-	+	-	-	-	-	-	-	-	-	-	-	-	-	+	+
Hypothetical protein associated with desferrioxamine E biosynthesis	+	+	+	-	-	-	-	-	-	-	-	-	-	+	-	+	-	-	+	-	-	-	-	-	-	-	-	-	-	-	+	+
Putative Desferrioxamine E transporter	+	+	-	+	+	+	-	-	-	-	-	-	-	+	-	+	-	+	+	+	-	+	-	+	-	-	-	-	-	+	+	+
**Ferrous iron transporter EfeUOB**																																
Ferrous iron transport periplasmic protein EfeO, contains peptidase-M75 domain and (frequently) cupredoxin-like domain	-	-	-	-	-	-	-	-	-	-	+	+	-	-	-	-	-	-	-	-	-	-	-	-	-	-	+	-	-	-	-	+
Ferrous iron transport permease EfeU	-	-	-	-	-	-	-	-	-	-	+	+	-	-	-	-	-	-	-	-	-	-	-	-	-	-	+	-	-	-	-	+
Ferrous iron transport peroxidase EfeB	-	-	-	-	-	-	-	-	-	-	+	+	-	-	-	-	-	-	-	-	-	-	-	-	-	-	+	-	-	-	-	+
**Siderophore assembly kit**																																
2,3-dihydroxybenzoate-AMP ligase (EC 2.7.7.58)	-	-	-	-	-	-	-	-	-	-	-	-	-	-	-	-	-	+	-	-	-	-	-	-	-	-	-	-	-	-	-	+
2,3-dihydroxybenzoate-AMP ligase (EC 2.7.7.58) of siderophore biosynthesis	-	-	-	-	-	-	-	-	-	-	-	-	-	-	-	+	-	-	-	-	-	-	-	-	-	-	-	-	-	-	-	-
ABC-type Fe3+-siderophore transport system, ATPase component	-	-	-	-	-	-	-	-	-	+	-	-	-	-	-	+	-	+	+	-	-	+	-	-	-	-	-	-	-	-	+	+
ABC-type Fe3+-siderophore transport system, periplasmic iron-binding component	-	-	-	-	-	-	-	-	-	+	-	-	-	-	-	-	-	+	-	-	-	+	-	-	-	-	-	-	-	-	-	-
ABC-type Fe3+-siderophore transport system, permease 2 component	-	-	-	-	-	-	-	-	-	+	-	-	-	-	-	+	-	-	+	-	-	+	-	-	-	-	-	-	-	-	+	+
ABC-type Fe3+-siderophore transport system, permease component	-	-	-	-	-	-	-	-	-	+	-	-	-	-	-	+	-	+	+	-	-	+	-	-	-	-	-	-	-	-	+	+
Ferric hydroxamate ABC transporter (TC 3.A.1.14.3), ATP-binding protein FhuC	-	-	-	-	-	-	-	-	-	+	-	-	-	-	-	-	-	+	+	-	-	+	-	-	-	-	-	-	-	-	-	+

Ferric hydroxamate ABC transporter (TC 3.A.1.14.3), periplasmic substrate binding protein FhuD	-	-	-	-	-	-	-	-	-	+	-	-	-	-	-	-	-	+	+	-	-	+	-	-	-	-	-	-	-	-	-	+
Ferric hydroxamate ABC transporter (TC 3.A.1.14.3), permease component FhuB	-	-	-	-	-	-	-	-	-	-	-	-	-	-	-	-	-	+	+	-	-	+	-	-	-	-	-	-	-	-	+	+
Iron-siderophore transport system, ATP-binding component	-	-	-	-	-	-	-	-	-	-	-	-	-	-	-	-	-	+	-	-	-	-	-	-	-	-	-	-	-	-	-	-
Iron-siderophore transport system, permease component	-	-	-	-	-	-	-	-	-	-	-	-	-	-	-	+	-	+	-	-	-	-	-	-	-	-	-	-	-	-	-	-
Iron-siderophore transport system, transmembran component	-	-	-	-	-	-	-	-	-	-	-	-	-	-	-	+	-	+	-	-	-	-	-	-	-	-	-	-	-	-	+	-
Putative ABC iron siderophore transporter, fused permease and ATPase domains	-	-	-	-	-	-	-	-	-	-	-	-	-	-	-	-	-	-	-	-	-	-	-	-	-	-	-	-	-	-	+	-
Putative reductoisomerase in siderophore biosynthesis gene cluster	-	-	-	-	-	-	-	-	-	-	-	-	-	-	-	-	-	-	-	-	-	+	-	-	-	-	-	-	-	-	+	-
Siderophore biosynthesis diaminobutyrate--2-oxoglutarate aminotransferase (EC 2.6.1.76)	-	-	-	-	-	-	-	-	-	-	-	-	-	-	-	-	-	-	-	-	-	+	-	-	-	-	-	-	-	-	-	+
Siderophore biosynthesis L-2,4-diaminobutyrate decarboxylase	-	-	-	-	-	-	-	-	-	+	-	-	-	-	-	+	-	+	+	-	-	+	-	-	-	-	-	-	-	-	+	+
Siderophore biosynthesis non-ribosomal peptide synthetase modules	-	-	-	-	-	-	-	-	-	-	-	-	-	-	-	+	-	-	-	-	-	+	-	-	-	-	-	-	-	-	+	+
Siderophore biosynthesis protein, monooxygenase	-	-	-	-	-	-	-	-	-	+	-	-	-	-	-	+	-	+	+	-	-	+	-	-	-	-	-	-	-	-	+	+
Siderophore synthetase component, ligase	-	-	-	-	-	-	-	-	-	+	-	-	-	-	-	+	-	+	+	-	-	+	-	-	-	-	-	-	-	-	+	+
Siderophore synthetase large component, acetyltransferase	-	-	-	-	-	-	-	-	-	+	-	-	-	-	-	+	-	-	-	-	-	+	-	-	-	-	-	-	-	-	+	+
Siderophore synthetase small component, acetyltransferase	-	-	-	-	-	-	-	-	-	+	-	-	-	-	-	+	-	+	-	-	-	-	-	-	-	-	-	-	-	-	+	+
Thiazolinyl imide reductase in siderophore biosynthesis gene cluster	-	-	-	-	-	-	-	-	-	-	-	-	-	-	-	-	-	-	-	-	-	-	-	-	-	-	-	-	-	-	+	-
Thioesterase in siderophore biosynthesis gene cluster	-	-	-	-	-	-	-	-	-	-	-	-	-	-	-	+	-	-	-	-	-	-	-	-	-	-	-	-	-	-	-	+

Table S7. Some putative stress related genes detected in the genomes of selected isolates. In total, 32 isolates were screened.

Function	KC06	KC216	KC345	6K520	7K507	7K534	KC603^T	5K138^T	8K307^T	16K104^T	5K110	KC207	KC213	KC606	KC721	KC723	13K206^T	15K316	KC13^T	KC201^T	KC310^T	KC333^T	KC401	KC712^T	6K102^T	5K548^T	7K502^T	16K309^T	16K404^T	7K107^T	8K308	13K301^T
**Cold shock**																																
protein cspA	-	-	-	-	-	-	-	-	-	-	-	-	-	-	-	-	-	-	-	-	-	+	-	-	-	-	-	-	-	-	-	-
protein cspB, SCO4325	+	+	+	+	+	+	+	+	+	+	+	+	+	+	+	+	+	+	+	+	+	+	+	+	+	+	+	+	+	+	+	+
protein cspC	-	-	-	-	-	-	-	-	-	-	-	-	-	-	-	-	-	-	-	-	-	+	-	-	-	-	-	-	-	-	-	-
**Heat shock**																																
protein hsp20	-	-	-	-	+	-	-	+	+	-	-	+	+	+	+	+	+	+	-	-	-	-	-	-	-	-	+	-	-	-	-	-
protein hsp22.5	+	+	-	-	-	-	-	-	-	-	-	-	-	-	-	-	-	-	-	-	-	-	-	-	-	-	-	-	-	-	-	-
protein htrA	-	-	-	-	-	-	-	-	-	-	-	+	-	-	-	-	-	-	-	-	-	-	-	-	-	-	-	-	-	-	-	-
**Osmotic stress**																																
Aquaporin Z protein	+	+	-	-	-	-	+	+	+	+	+	+	+	+	+	+	+	+	+	-	-	+	-	-	-	-	-	-	-	-	+	-
Cyclic beta-1,2-glucan synthase (EC 2.4.1.-)	-	-	-	-	-	-	-	-	-	-	-	-	+	+	+	-	-	-	-	-	-	-	-	-	-	-	-	-	-	-	-	-
Glycerol uptake facilitator protein	+	+	+	+	+	+	+	+	+	+	+	-	-	-	-	-	-	-	+	+	+	+	+	+	+	+	+	+	+	+	+	+
Outer membrane protein A precursor	-	-	+	+	+	-	-	-	-	-	-	-	-	-	-	-	-	-	-	-	-	-	-	+	-	-	-	-	-	-	-	+
**Oxidative stress**																																
Alkyl hydroperoxide reductase subunit C-like protein	+	+	+	+	+	+	+	+	+	-	-	+	+	+	+	+	+	+	+	+	+	+	+	+	+	+	+	+	+	+	+	+
Choline binding ABC transport system ATP-binding protein choV	-	-	-	-	-	-	-	+	-	-	-	-	-	-	-	-	-	-	-	-	-	-	-	-	+	-	-	-	-	-	-	-
Choline kinase (EC 2.7.1.32)	-	-	-	-	-	-	-	-	-	-	-	-	-	-	-	-	-	-	-	+	+	+	+	+	+	-	-	-	-	-	-	-
Choline-sulfatase (EC 3.1.6.6)	-	-	+	-	+	-	+	+	+	-	-	-	-	-	-	-	-	-	-	+	+	+	+	+	+	-	-	+	-	+	-	-
Choline oxidase (EC 1.1.3.17)	-	-	-	-	-	-	-	-	-	-	-	-	-	-	-	-	-	-	-	-	-	-	-	-	-	+	+	+	+	-	-	+
Catalase-peroxidase katG (EC 1.11.1.21)	+	+	+	+	+	+	-	-	-	+	+	+	+	+	+	+	+	+	+	+	+	+	+	+	+	+	+	+	+	-	+	+
Choline dehydrogenase (EC 1.1.99.1)	-	-	-	-	-	-	-	-	+	-	-	-	-	-	-	-	-	-	+	+	+	+	+	+	+	+	-	-	-	-	-	+
Flavohemoglobin, nitric oxide dioxygenase	+	+	+	+	+	+	+	+	+	-	-	+	+	+	+	+	+	+	-	+	+	+	+	+	+	+	+	+	+	+	+	+
Glucose-methanol-choline (GMC) oxidoreductase	+	+	+	+	+	+	-	+	-	+	+	-	+	-	+	-	-	+	+	-	-	-	-	-	-	+	+	-	-	+	+	+
High-affinity choline uptake protein BetT	-	-	+	-	+	-	-	-	-	+	+	-	-	-	-	-	-	-	+	-	-	-	-	-	-	+	+	+	+	-	-	+
Manganese catalase (EC 1.11.1.6)	-	-	-	-	-	-	+	+	-	+	+	-	-	-	-	-	-	+	-	-	-	-	-	-	-	+	+	+	+	-	-	-
Nitrite-sensitive transcriptional repressor	-	-	-	+	-	-	-	-	-	-	-	-	-	-	-	-	-	+	-	-	-	+	-	-	-	-	+	+	-	+	-	+
Phytochrome, two component sensor histidine kinase	+	+	+	+	+	-	-	-	-	-	-	+	+	+	+	+	+	+	-	+	+	+	+	+	+	-	-	-	-	-	-	-
Sarcosine oxidase (EC 1.5.3.1)	+	+	-	-	+	-	-	+	-	+	-	-	-	+	-	-	-	-	+	+	+	+	+	+	+	+	+	+	+	+	+	+
Transcriptional regulator crp/fnr family	+	+	+	+	+	+	-	-	+	-	-	-	-	-	-	-	+	-	-	+	+	+	+	+	+	-	-	-	-	+	+	-
**Carbon starvation**																																
Carbon starvation protein A	-	-	+	+	+	+	+	+	+	+	-	-	-	-	-	-	-	-	+	+	+	+	+	+	+	+	+	+	+	-	+	+

Starvation sensing protein RspA	-	-	-	-	-	-	-	+	-	+	-	-	-	-	-	-	-	-	-	-	-	-	-	-	-	-	-	-	-	-	-	-
**Carbon monoxide dehydrogenase maturation factors**																																
Carbon monoxide oxidation accessory protein CoxD	+	+	+	+	+	+	-	-	+	-	-	-	-	-	-	-	-	-	+	+	+	+	+	+	+	+	+	+	+	-	-	+
Carbon monoxide oxidation accessory protein CoxE	+	+	+	+	+	+	-	-	+	-	-	-	-	-	-	-	-	-	+	+	+	+	+	+	+	+	+	+	+	-	-	+
Carbon monoxide oxidation accessory protein CoxG	-	-	-	-	-	-	-	-	-	-	-	-	-	-	-	-	-	-	-	-	-	+	-	-	-	+	+	+	+	-	-	+
**Carotenoid production**																																
(2E,6E)-farnesyl diphosphate synthase (EC 2.5.1.10)	+	+	+	+	+	+	-	-	-	-	-	-	-	-	-	-	-	+	+	-	-	-	-	-	-	+	+	+	+	-	-	+
CrtT-methyltransferase-like protein	-	-	-	-	-	-	-	-	-	-	-	-	-	-	-	-	-	-	-	-	-	-	-	-	-	+	-	+	+	-	-	+
CrtV-methyltransferase-like protein	-	-	-	-	-	-	-	-	-	-	-	-	-	-	-	-	-	-	-	-	-	-	-	-	-	+	-	+	+	-	-	+
Geranylgeranyl diphosphate synthase (EC 2.5.1.29)	+	+	+	+	+	+	-	-	-	-	-	-	-	-	-	-	-	+	-	-	-	-	-	-	-	+	+	+	+	-	-	+
Lycopene cyclase	-	-	-	-	-	-	-	-	-	-	-	-	-	-	-	-	-	-	-	-	-	-	-	-	-	+	-	+	+	-	-	-
Neurosporene desaturase (EC 1.-.-.-)	-	-	-	+	+	+	-	-	-	-	-	-	-	-	-	-	-	-	-	-	-	-	-	-	-	-	-	-	-	-	-	-
phi-Carotenoid synthase (EC 1.3.-.- and EC 2.1.1-)	-	-	-	-	-	-	-	-	-	-	-	-	-	-	-	-	-	+	-	-	-	-	-	-	-	+	-	+	+	-	-	+
Phytoene dehydrogenase (EC 1.14.99.-)	+	+	+	+	+	+	-	-	-	-	-	-	-	-	-	-	-	+	-	-	-	-	-	-	-	-	-	-	-	-	-	-
Phytoene dehydrogenase and related proteins	+	-	+	+	+	+	-	-	-	-	-	-	-	-	-	-	-	+	+	-	-	-	-	-	-	+	+	+	+	-	-	+
Phytoene desaturase (EC 1.14.99.-)	-	-	-	+	+	+	-	-	-	-	-	-	-	-	-	-	-	-	-	-	-	-	-	-	-	-	-	-	-	-	-	+
Phytoene desaturase, neurosporene or lycopene producing (EC 1.3.-.-)	-	-	-	-	-	-	-	-	-	-	-	-	-	-	-	-	-	-	-	-	-	-	-	-	-	-	-	-	-	-	-	+
Phytoene synthase (EC 2.5.1.32)	+	+	+	+	+	+	-	-	-	-	-	-	-	-	-	-	-	+	-	-	-	-	-	-	-	+	+	+	+	-	-	+
Zeaxanthin glucosyl transferase	+	-	+	-	-	-	-	-	-	-	-	-	-	-	-	-	-	-	-	-	-	-	-	-	-	-	-	-	-	-	-	-
**One-carbon metabolism by tetrahydropterines**																																
5,10-methylenetetrahydrofolate reductase (EC 1.5.1.20)	+	+	+	+	+	+	+	+	+	+	-	+	+	+	+	+	+	+	+	+	+	+	+	+	+	+	+	+	+	+	+	+
5-formyltetrahydrofolate cyclo-ligase (EC 6.3.3.2)	+	+	+	+	+	+	+	+	+	+	+	+	+	+	+	+	+	+	+	+	+	+	+	+	+	+	+	+	+	+	+	+
Formate--tetrahydrofolate ligase (EC 6.3.4.3)	-	-	-	-	-	-	-	-	-	-	-	-	-	-	-	-	-	-	-	-	-	-	-	-	-	+	+	+	+	-	-	-
Formiminotetrahydrofolate cyclodeaminase (EC 4.3.1.4)	-	-	-	-	-	-	+	+	+	-	-	-	-	-	-	-	-	-	-	+	+	+	+	-	+	+	-	+	+	-	-	-
Formyltetrahydrofolate deformylase (EC 3.5.1.10)	+	+	+	+	+	+	-	-	-	+	+	+	+	-	+	+	+	+	+	+	+	+	+	+	+	+	+	+	+	+	+	+
Methenyltetrahydrofolate cyclohydrolase (EC 3.5.4.9)	+	+	+	+	+	+	+	+	+	+	+	+	+	+	+	+	+	+	+	+	+	+	+	+	+	+	+	+	+	+	+	+
Methylenetetrahydrofolate dehydrogenase (NADP+) (EC 1.5.1.5)	+	+	+	+	+	+	+	+	+	+	+	+	+	+	+	+	+	+	+	+	+	+	+	+	+	+	+	+	+	+	+	+
**CO_2 uptake**																																
High-affnity carbon uptake protein Hat/HatR	-	-	+	-	+	+	-	-	-	-	-	-	-	-	-	-	-	-	-	-	-	-	-	-	-	-	-	-	-	-	-	-
Ribulose bisphosphate carboxylase large chain (EC 4.1.1.39)	-	-	+	+	+	+	-	-	-	-	-	-	-	-	-	-	-	-	-	-	-	-	-	-	-	+	-	+	-	-	-	-
Ribulose bisphosphate carboxylase small chain (EC 4.1.1.39)	-	-	+	+	+	+	-	-	-	-	-	-	-	-	-	-	-	-	-	-	-	-	-	-	-	+	-	+	-	-	-	-

**Resistance to fluoroquinolones**																																
DNA gyrase subunit A (EC 5.99.1.3)	+	+	+	+	+	+	+	+	+	+	+	+	+	+	+	+	+	+	+	+	+	+	+	+	+	+	+	+	+	+	+	+
DNA gyrase subunit B (EC 5.99.1.3)	+	+	+	+	+	+	+	+	+	+	+	+	+	+	+	+	+	+	+	+	+	+	+	+	+	+	+	+	+	+	+	+
**Copper homeostasis**																																
Blue copper oxidase CueO precursor	+	+	-	-	-	-	-	-	-	-	-	+	-	-	+	-	+	-	-	+	-	-	-	-	+	-	-	-	-	-	-	-
Copper chaperone	-	-	+	-	+	-	-	+	-	-	+	-	-	-	-	-	+	+	-	+	+	-	-	-	-	-	+	-	-	-	+	-
Copper homeostasis protein CutE	-	-	-	-	-	-	-	-	+	-	-	-	-	-	-	-	-	-	-	-	-	-	-	-	-	-	-	-	-	-	-	-
Copper resistance protein CopC	-	+	+	+	+	+	+	-	+	+	+	+	+	+	+	+	+	+	+	+	+	+	+	+	+	+	+	+	+	+	+	+
Copper resistance protein CopD	+	+	+	+	+	+	+	+	+	+	+	+	+	+	+	+	+	+	+	+	+	+	+	+	+	+	+	+	+	-	+	+
Copper resistance protein D	-	-	-	-	-	-	-	+	-	-	-	-	-	-	-	-	-	-	+	-	-	-	-	-	-	-	-	-	-	-	-	-
Copper-translocating P-type ATPase (EC 3.6.3.4)	+	+	+	+	+	+	+	+	-	+	+	+	+	+	+	+	+	+	+	+	+	+	+	+	+	+	+	+	+	+	+	+
Cu(I)-responsive transcriptional regulator	-	-	-	-	-	-	-	-	-	-	+	-	-	-	-	-	-	-	-	-	-	-	-	-	-	-	-	-	-	-	-	-
Cytochrome c heme lyase subunit CcmF	-	-	-	-	-	-	-	-	-	-	-	-	-	-	-	-	+	+	-	-	-	-	-	-	-	-	-	-	-	-	-	-
Cytoplasmic copper homeostasis protein CutC	+	+	+	+	+	+	+	+	+	+	+	-	-	-	-	-	+	+	+	+	+	+	+	+	+	+	+	+	+	+	-	+
Magnesium and cobalt efflux protein CorC	+	+	+	+	+	+	+	+	+	+	+	+	+	+	+	+	+	+	+	+	+	+	+	+	+	+	+	+	+	+	+	+
Multicopper oxidase	-	-	-	+	+	+	+	+	+	+	+	+	+	+	+	+	+	+	-	+	+	+	+	+	+	-	-	-	-	+	+	+
Multidrug resistance transporter, Bcr/CflA family	+	-	+	+	+	+	+	+	+	-	+	+	-	+	-	+	+	+	+	+	+	+	+	+	+	+	+	+	+	+	+	+
Periplasmic divalent cation tolerance protein CutA	+	+	+	+	+	+	+	+	+	-	+	+	+	+	+	+	+	+	+	+	-	-	-	-	-	+	+	+	+	+	+	-
**Resistance to vancomycin**																																
Vancomycin response regulator VanR	-	+	+	+	+	+	-	+	+	+	-	-	-	-	-	-	-	-	-	-	-	-	-	-	-	-	+	-	-	-	-	-
Vancomycin resistance protein VanH	-	-	-	-	-	-	-	+	+	-	-	-	-	-	-	-	-	-	-	-	-	-	-	-	-	-	-	-	-	-	-	-
Vancomycin B-type resistance protein VanX	-	-	-	-	-	-	-	+	+	-	-	-	-	-	-	-	-	-	-	-	-	-	-	-	-	-	-	-	-	-	-	-
Vancomycin B-type resistance protein VanW	-	+	+	+	+	+	-	+	+	+	-	+	+	+	+	+	-	+	-	-	-	-	-	-	-	+	+	+	+	-	+	-
**Arsenic resistance**																																
Arsenate reductase (EC 1.20.4.1)	-	-	-	-	-	-	-	-	-	-	+	+	-	-	-	-	-	+	+	+	-	-	-	+	-	-	+	+	-	-	-	-
Arsenic efflux pump protein	-	-	-	-	-	-	-	-	-	-	+	+	-	-	-	-	-	+	-	+	-	-	-	-	-	-	-	-	-	-	-	-
Arsenical resistance operon repressor	-	-	-	-	-	-	-	-	-	-	+	+	-	-	-	-	-	+	+	+	-	-	-	+	-	-	+	+	-	-	-	-
Arsenical-resistance protein ACR3	-	-	-	-	-	-	-	-	-	-	+	-	-	-	-	-	-	-	-	+	-	-	-	+	-	-	+	+	-	-	-	-
**Mercuric reductase**																																
Mercuric ion reductase (EC 1.16.1.1)	-	-	-	-	-	-	-	-	-	-	+	-	-	-	-	-	+	+	-	-	-	+	-	-	-	-	-	-	-	-	-	-
PF00070 family, FAD-dependent NAD(P)-disulphide oxidoreductase	+	+	+	+	+	+	+	+	+	-	+	+	-	+	-	-	-	-	+	+	+	+	-	+	-	+	+	+	+	+	+	+
**Cobalt-zinc-cadmium resistance**																																
Cation efflux system protein CusA	+	-	-	+	+	-	+	-	-	-	-	-	-	-	-	-	-	-	-	-	-	-	-	+	-	-	-	-	-	+	-	-
Cobalt-zinc-cadmium resistance protein	+	+	+	+	+	+	-	-	-	-	-	-	-	-	-	+	+	-	-	+	-	+	+	+	-	+	+	+	+	-	+	-
Cobalt-zinc-cadmium resistance protein CzcA	+	-	-	+	+	-	+	-	-	-	-	-	-	-	-	-	-	-	-	-	-	-	-	+	-	-	-	-	-	+	-	-
Cobalt-zinc-cadmium resistance protein CzcD	+	+	+	+	+	+	+	+	+	+	+	+	+	+	+	+	-	+	+	+	+	+	+	+	+	+	+	+	+	-	+	-
DNA-binding heavy metal response regulator	-	-	-	-	-	-	-	-	-	-	-	-	+	-	+	-	-	-	-	+	+	-	+	+	+	-	+	-	-	-	-	-

Probable Co/Zn/Cd efflux system membrane fusion protein	-	-	-	-	-	-	-	-	-	-	-	-	-	-	-	-	-	-	-	-	+	-	-	+	-	-	-	-	-	-	-	-
Transcriptional regulator, MerR family	+	+	+	+	+	+	+	+	+	+	+	+	+	+	+	+	+	+	+	+	+	+	+	+	+	+	+	+	+	+	+	+
**<u>Beta-lactamase</u>**																																
Beta-lactamase class C and other penicillin binding proteins	+	-	-	-	+	-	-	-	-	+	-	-	-	-	-	-	-	-	+	-	-	-	-	-	-	+	-	-	-	-	-	-
Metal-dependent hydrolases of the beta-lactamase superfamily III	+	+	+	+	+	+	+	+	+	+	-	+	+	+	+	+	+	+	+	+	+	+	+	+	+	+	+	+	+	+	+	-
Beta-lactamase (Cephalosporinase) (EC 3.5.2.6)	-	-	-	-	-	-	-	-	-	+	-	-	-	-	-	-	-	-	-	-	-	-	-	-	-	-	-	-	-	-	-	-
Beta-lactamase (EC 3.5.2.6)	-	-	-	-	+	-	+	-	+	+	-	-	-	-	-	-	-	-	+	+	-	-	-	-	-	-	-	+	-	-	-	-
Class A beta-lactamase (EC 3.5.2.6)	+	+	+	-	+	+	+	-	+	+	-	+	+	+	+	-	+	+	+	+	+	+	+	+	+	+	+	-	+	+	+	+
Class D beta-lactamase (EC 3.5.2.6)	-	-	+	+	-	+	-	-	-	-	-	-	-	-	-	-	-	-	-	-	-	-	-	-	-	-	-	-	-	-	-	-
**<u>Resistance to chromium compounds</u>**																																
Chromate transport protein ChrA	-	-	-	-	-	-	-	-	-	-	+	+	-	-	-	-	-	-	+	+	+	-	+	+	+	-	-	-	-	-	-	-
Superoxide dismutase SodM-like protein ChrF	-	-	-	-	-	-	-	-	-	-	-	+	-	-	-	-	-	-	-	-	-	-	-	-	-	-	-	-	-	-	-	-

Table S8. Genes associated with DNA repair detected in the genomes of selected isolates. In total, 32 isolates were screened.

Function	KC06	KC216	KC345	6K520	7K507	7K534	KC603^T	5K138^T	8K307^T	16K104^T	5K110	KC207	KC213	KC606	KC721	KC723	13K206^T	15K316	KC13^T	KC201^T	KC310^T	KC333^T	KC401	KC712^T	6K102^T	5K548^T	7K502^T	16K309^T	16K404^T	7K107^T	8K308	13K301^T
Alkylated DNA repair protein alkB	+	+	+	+	+	+	+	+	+	+	-	+	+	+	+	+	+	+	+	+	+	+	+	+	+	-	+	-	-	-	+	-
ATP dependant helicase SCO5183	+	+	+	+	+	+	+	+	+	+	+	+	+	+	+	+	+	+	+	+	+	+	+	+	+	+	+	+	+	+	+	+
ATP dependant helicase SCO5184	+	+	+	+	+	+	+	+	+	+	+	+	+	+	+	+	+	+	+	+	+	+	+	+	+	+	+	+	+	+	+	+
ATP-dependent DNA helicase	+	+	+	+	+	+	-	-	-	+	-	+	+	+	+	+	+	+	+	+	+	+	+	+	+	+	+	+	+	+	+	+
ATP-dependent DNA helicase RecG (EC 3.6.4.12)	+	+	+	+	+	+	+	+	+	+	+	+	+	+	+	+	+	+	+	+	+	+	+	+	+	+	+	+	+	+	+	+
ATP-dependent DNA helicase recQ	+	+	+	+	+	+	+	+	+	+	+	+	+	+	+	+	+	+	+	+	+	+	+	+	+	+	+	+	+	+	+	+
ATP-dependent DNA helicase uvrD/pcrA (EC 3.6.4.12)	+	+	+	+	+	+	+	+	+	+	+	+	+	+	+	+	+	+	+	+	+	+	+	+	+	+	+	+	+	+	+	+
ATP-dependent DNA helicase, actinomycete paralog	+	+	+	+	+	+	+	+	+	+	+	+	+	+	+	+	+	+	+	+	+	+	+	+	+	+	+	+	+	+	+	+
ATP-dependent DNA ligase (EC 6.5.1.1)	+	+	+	+	+	+	+	+	+	+	-	+	+	+	+	+	+	+	+	+	+	+	+	+	+	+	+	+	+	+	+	+
ATP-dependent DNA ligase (EC 6.5.1.1) clustered with Ku protein, ligD	+	+	+	+	+	+	+	+	+	+	+	+	+	+	+	+	+	+	+	+	+	+	+	+	+	+	+	+	+	+	+	+
ATP-dependent DNA ligase (EC 6.5.1.1) ligC	+	+	+	+	+	+	+	+	+	+	+	+	+	+	+	+	+	+	+	+	+	+	+	+	+	+	+	+	+	+	+	+
DNA recombination and repair protein recF	+	+	+	+	+	+	+	+	+	-	+	+	+	+	+	+	+	+	+	+	+	+	+	+	+	+	+	+	+	+	+	+
DNA recombination and repair protein recO	+	+	+	+	+	+	+	+	+	-	+	+	+	+	+	+	+	+	+	+	+	+	+	+	+	+	+	+	+	+	+	+
DNA recombination protein rmuC	+	+	+	+	+	+	+	+	+	+	+	+	+	+	+	+	+	+	+	+	+	+	+	+	+	+	+	+	+	+	-	-
DNA repair protein radA	+	+	+	+	+	+	+	+	+	+	+	+	+	+	+	+	+	+	+	+	+	+	+	+	+	+	+	+	+	+	+	+
DNA repair protein radC	-	-	-	-	-	-	-	-	-	-	-	-	-	-	-	-	-	-	-	-	-	-	-	+	-	-	-	-	-	-	-	-
DNA repair protein recN	+	+	+	+	+	+	+	+	+	+	+	+	+	+	+	+	+	+	+	+	+	+	+	+	+	+	+	+	+	+	+	+
Endonuclease III (EC 4.2.99.18)	+	+	+	+	+	+	+	+	+	+	+	+	+	+	+	+	+	+	+	+	+	+	+	+	+	+	+	+	+	+	+	+
Endonuclease IV (EC 4.2.21.2)	+	+	+	+	+	+	+	+	+	+	+	+	+	+	+	+	+	+	+	+	+	+	+	+	+	+	+	+	+	+	+	+
Endonuclease V (EC 4.2.21.7)	+	+	+	+	+	+	-	-	-	+	+	+	+	+	+	+	+	+	-	+	+	+	+	+	+	+	+	+	+	+	+	+
Endonuclease VIII	+	+	+	+	+	+	+	+	+	+	+	+	+	+	+	+	+	+	+	+	+	+	+	+	+	+	+	+	+	+	+	+
Excinuclease ABC subunit A	+	+	+	+	+	+	+	+	+	+	+	+	+	+	+	+	+	+	+	+	+	+	+	+	+	+	+	+	+	+	+	+
Excinuclease ABC subunit A paralog of unknown function	+	+	+	+	+	+	+	+	+	+	+	+	+	+	+	+	+	+	+	+	+	+	+	+	+	+	+	+	+	+	+	+
Excinuclease ABC subunit B	+	+	+	+	+	+	+	+	+	+	+	+	+	+	+	+	+	+	+	+	+	+	+	+	+	+	+	+	+	+	+	+
Excinuclease ABC subunit C	+	+	+	+	+	+	+	+	+	+	+	+	+	+	+	+	+	+	+	+	+	+	+	+	+	+	+	+	+	+	+	+
Exodeoxyribonuclease III (EC 3.1.11.2)	+	+	+	+	+	+	+	+	+	+	+	+	+	+	+	+	+	+	+	+	+	+	+	+	+	+	+	+	+	+	+	+
Exodeoxyribonuclease V alpha chain (EC 3.1.11.5)	-	-	-	-	-	-	-	-	-	+	-	-	-	-	-	-	-	-	+	-	-	-	-	-	-	-	-	-	-	-	-	-

Exodeoxyribonuclease V beta chain (EC 3.1.11.5)	-	-	-	-	-	-	-	-	-	+	-	-	-	-	-	-	+	+	+	-	-	-	-	-	-	-	-	-	-	-	-	-
Exodeoxyribonuclease V gamma chain (EC 3.1.11)	-	-	-	-	-	-	-	-	-	+	-	-	-	-	-	-	-	-	+	-	-	-	-	-	-	-	-	-	-	-	-	-
Exodeoxyribonuclease VII large subunit (EC 3.1.11.6)	+	+	+	+	+	+	+	+	+	+	+	+	+	+	+	+	+	+	+	+	+	+	+	+	+	+	+	+	+	+	+	+
Exodeoxyribonuclease VII small subunit (EC 3.1.11.6)	+	+	+	+	+	+	+	+	+	+	+	+	+	+	+	+	+	+	+	+	+	+	+	+	+	+	+	+	+	+	+	+
Exonuclease sbcC	+	+	+	+	+	+	-	-	-	-	+	+	+	+	+	+	+	+	+	+	+	+	+	+	+	+	+	+	+	+	+	+
Exonuclease sbcD	+	+	+	+	+	+	-	-	-	-	+	+	+	+	+	+	+	-	+	+	+	+	+	+	+	+	+	+	+	+	+	+
Holliday junction ATP-dependent DNA helicase RuvA (EC 3.6.4.12)	+	+	+	+	+	+	+	+	+	+	+	+	+	+	+	+	+	+	+	+	+	+	+	+	+	+	+	+	+	-	+	+
Holliday junction ATP-dependent DNA helicase RuvB (EC 3.6.4.12)	+	+	+	+	+	+	+	+	+	+	+	+	+	+	+	+	+	+	+	+	+	+	+	+	+	+	+	+	+	-	+	+
RecA protein	+	+	+	+	+	+	+	+	+	+	+	+	+	+	+	+	+	+	+	+	+	+	+	+	+	+	+	+	+	+	+	+
RecD-like DNA helicase yrrC	+	+	+	+	+	+	-	-	-	-	-	+	+	+	+	+	+	-	-	+	+	+	+	+	+	-	-	-	-	+	+	+
Recombination protein recR	+	+	+	+	+	+	+	+	+	-	+	+	+	+	+	+	+	+	+	+	+	+	+	+	+	+	+	+	+	+	+	+
Recombinational DNA repair protein recT	+	-	-	-	-	-	-	-	-	-	-	-	-	-	-	-	-	-	-	-	-	-	-	-	-	-	-	-	-	-	-	-
Regulatory protein recX	+	+	-	+	-	+	+	+	+	+	+	+	+	+	+	+	+	+	+	+	+	+	+	+	+	+	+	+	+	+	+	+
SOS-response repressor and protease lexA (EC 3.4.21.88)	+	+	+	+	+	+	+	+	+	+	+	+	+	+	+	+	+	+	+	+	+	+	+	+	+	+	+	+	+	+	+	+
Very-short-patch mismatch repair endonuclease (G-T specific)	-	+	-	-	-	+	+	-	-	+	-	-	-	+	-	+	-	-	-	-	-	-	-	-	-	+	+	+	+	-	+	+

Table S9. Genes associated with sporulation detected in the genomes of selected isolates. In total, 32 isolates were screened.

Function	KC06	KC216	KC345	6K520	7K507	7K534	KC603[T]	5K138[T]	8K307[T]	16K104[T]	5K110	KC207	KC213	KC606	KC721	KC723	13K206[T]	15K316	KC13[T]	KC201[T]	KC310[T]	KC333[T]	KC401	KC712[T]	6K102[T]	5K548[T]	7K502[T]	16K309[T]	16K404[T]	7K107[T]	8K308	13K301[T]
Aromatase WhiE VI	-	-	-	-	-	-	-	-	-	-	-	+	-	+	+	-	+	+	-	+	-	+	+	-	+	-	-	-	-	+	-	+
Cell division inhibitor	+	+	+	+	+	+	+	+	+	+	+	+	+	+	+	+	+	+	+	+	+	+	+	+	+	+	+	+	+	+	+	+
Peptidyl-tRNA hydrolase (EC 3.1.1.29)	+	+	+	+	+	+	+	+	+	+	+	+	+	+	+	+	+	+	+	+	+	+	+	+	+	+	+	+	+	+	+	+
Polyketide beta-ketoacyl synthase WhiE-KS	-	-	-	-	-	-	-	-	-	-	-	+	+	+	+	+	+	+	-	-	-	-	-	-	-	-	-	-	-	+	-	+
Polyketide beta-ketoacyl synthase WhiE-KS paralog	+	-	+	+	+	+	-	-	-	-	-	-	+	+	+	-	-	-	-	+	-	+	+	-	+	-	-	-	-	+	-	-
Polyketide chain length factor WhiE-CLF	-	-	-	-	-	-	-	-	-	-	-	+	+	+	+	+	+	+	-	-	-	-	-	-	-	-	-	-	-	+	-	+
Polyketide chain length factor WhiE-CLF paralog	+	-	+	+	+	+	-	-	-	-	-	-	+	+	+	-	-	-	-	+	-	+	+	-	+	-	-	-	-	+	-	-
Polyketide cyclase WhiE II	-	-	-	-	-	-	-	-	-	-	-	+	+	+	+	+	+	+	-	+	-	+	+	-	+	-	-	-	-	+	-	+
Polyketide cyclase WhiE VII	-	-	+	+	+	+	-	-	-	-	-	+	+	+	+	+	+	+	-	+	-	-	+	-	+	-	-	-	-	+	-	+
Polyketide hydroxylase WhiE VIII	-	-	-	-	-	-	-	-	-	-	-	-	-	-	-	-	-	-	-	+	-	-	-	-	-	-	-	-	-	-	-	+
Sporulation control protein Spo0M	+	+	+	+	+	+	-	-	-	-	-	-	-	+	+	-	-	-	+	-	+	+	+	+	+	+	+	+	-	+	+	+
Sporulation regulatory protein WhiB	+	+	+	+	+	+	+	+	+	+	+	+	+	+	+	+	+	+	+	+	+	+	+	+	+	+	+	+	+	+	+	+
Sporulation regulatory protein WhiD	-	-	-	-	-	-	+	+	+	-	-	+	+	+	+	+	+	+	-	-	-	-	-	-	-	+	+	+	+	-	+	+
Sporulation transcription regulator WhiA	-	-	-	-	-	-	-	-	-	-	-	-	-	-	-	-	-	-	+	-	-	-	-	-	-	-	-	-	-	-	-	-
Stage 0 sporulation protein YaaT	+	+	+	+	+	+	+	+	+	+	-	+	+	+	+	+	+	-	+	-	+	+	+	+	+	-	-	-	-	+	+	-
Stage II sporulation protein E-like	+	+	+	+	-	-	-	-	-	-	-	+	+	+	+	+	+	-	+	-	+	+	+	+	+	-	-	-	-	+	+	+
Stage V sporulation protein K	-	-	-	-	-	-	-	-	-	-	-	-	-	-	-	-	-	-	-	-	-	-	-	-	-	+	+	+	+	-	-	-
Stage V sporulation protein SpoVK	-	-	-	-	-	-	-	-	-	-	-	-	-	-	-	-	-	+	-	-	-	-	-	-	-	-	-	-	-	-	-	-
WhiB-family transcriptional regulator	-	-	-	-	-	-	-	-	-	-	-	-	-	-	-	-	-	-	+	-	-	-	-	-	-	-	-	-	-	-	+	-
WhiB-like transcription regulator	+	+	+	+	+	+	+	+	+	+	+	+	+	+	+	+	+	+	+	+	+	+	+	+	+	+	+	+	+	+	+	+
WhiB-like transcriptional regulator	+	+	+	+	+	+	-	-	-	+	-	-	-	-	-	-	-	-	-	+	+	+	+	+	+	-	-	-	-	+	-	-
WhiE I protein of unknown function	-	-	-	-	-	-	-	-	-	-	-	+	+	+	+	+	+	+	-	+	-	-	+	-	-	-	-	-	-	+	-	+

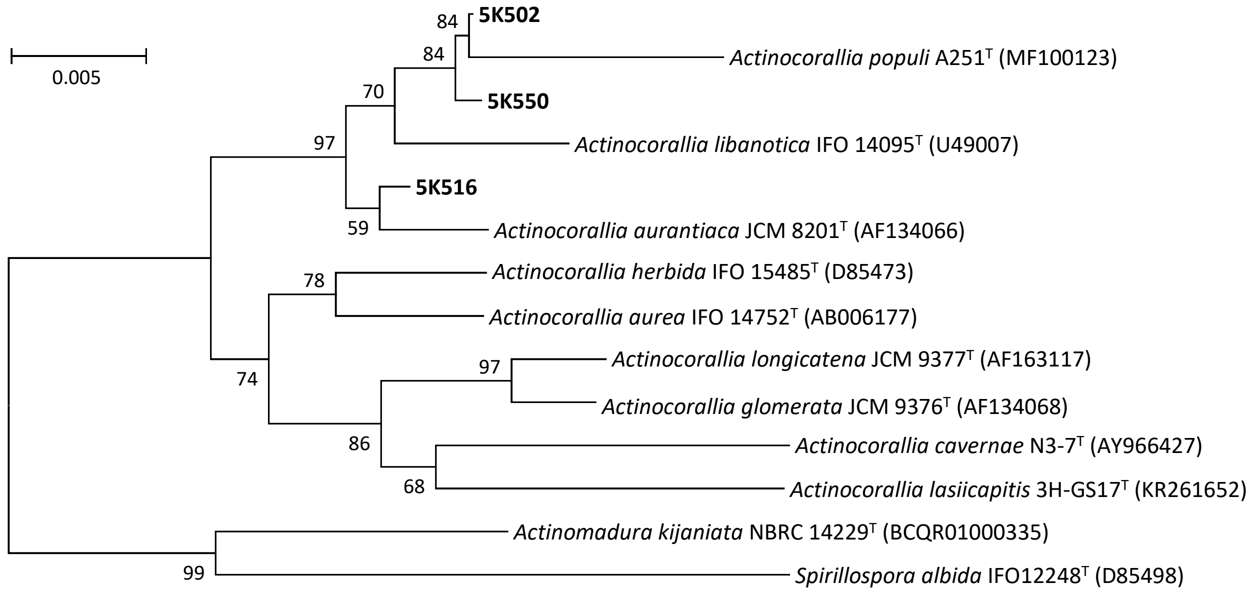

Fig. S1. Neighbor-joining phylogenetic tree based on 16S rRNA gene sequences showing relationships between isolates and closely related type strains of the genus *Actinocorallia*. Numbers at the nodes indicate percentage levels of bootstrap support; only values over 50 % are shown. There were a total of 1371 positions in the final dataset. Bar, 0.005 substitutions per nucleotide position. The tree is rooted using the type strain of *Spirillospora albida*, the type species of the genus.

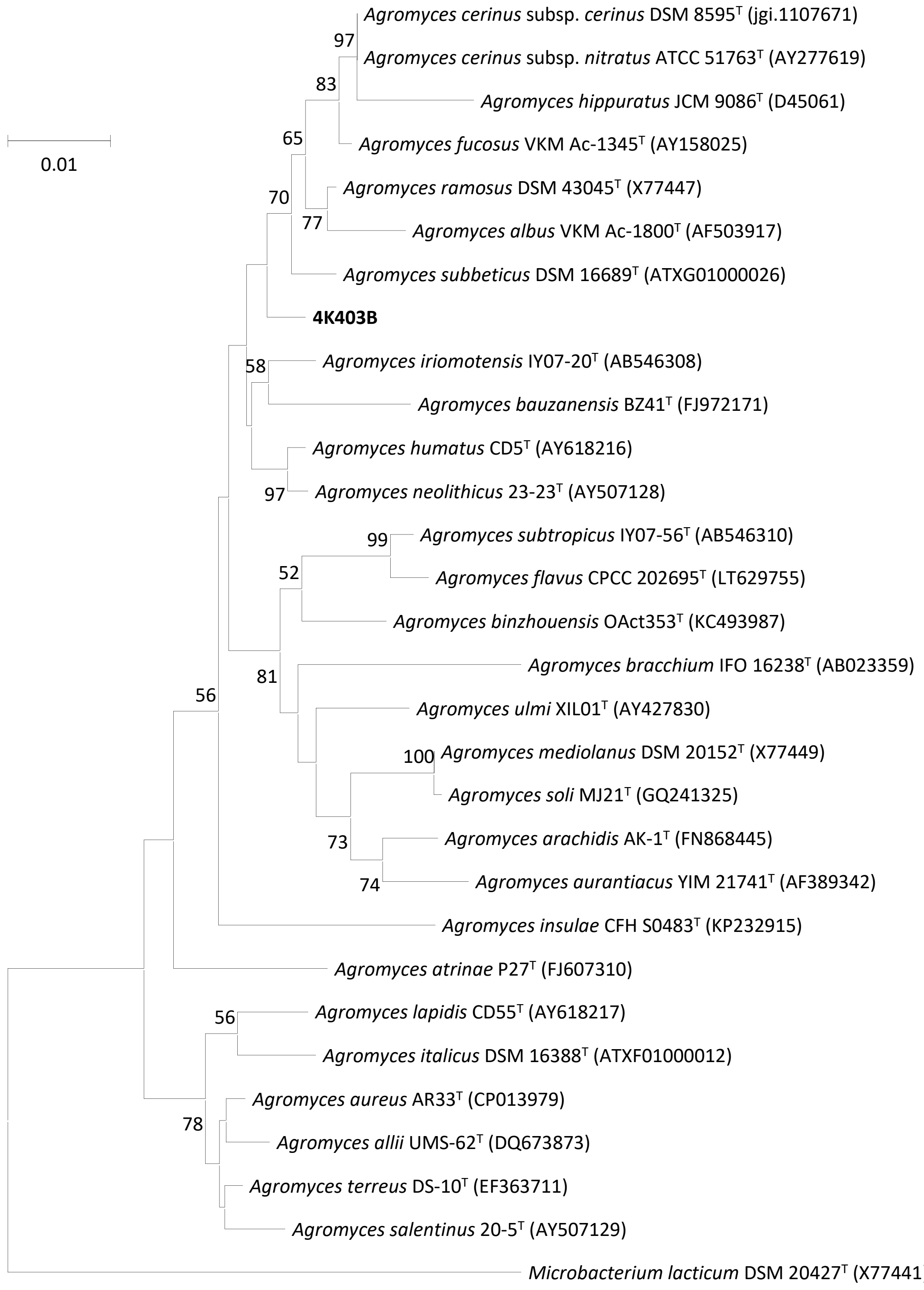

Fig. S2. Neighbor-joining phylogenetic tree based on 16S rRNA gene sequences showing relationships between isolate 4K403B and closely related type strains of the genus *Agromyces*. Numbers at the nodes indicate percentage levels of bootstrap support; only values over 50 % are shown. There were a total of 1395 positions in the final dataset. Bar, 0.01 substitutions per nucleotide position. The tree is rooted using the type strain of *Microbacterium lacticum*, the type species of the genus.

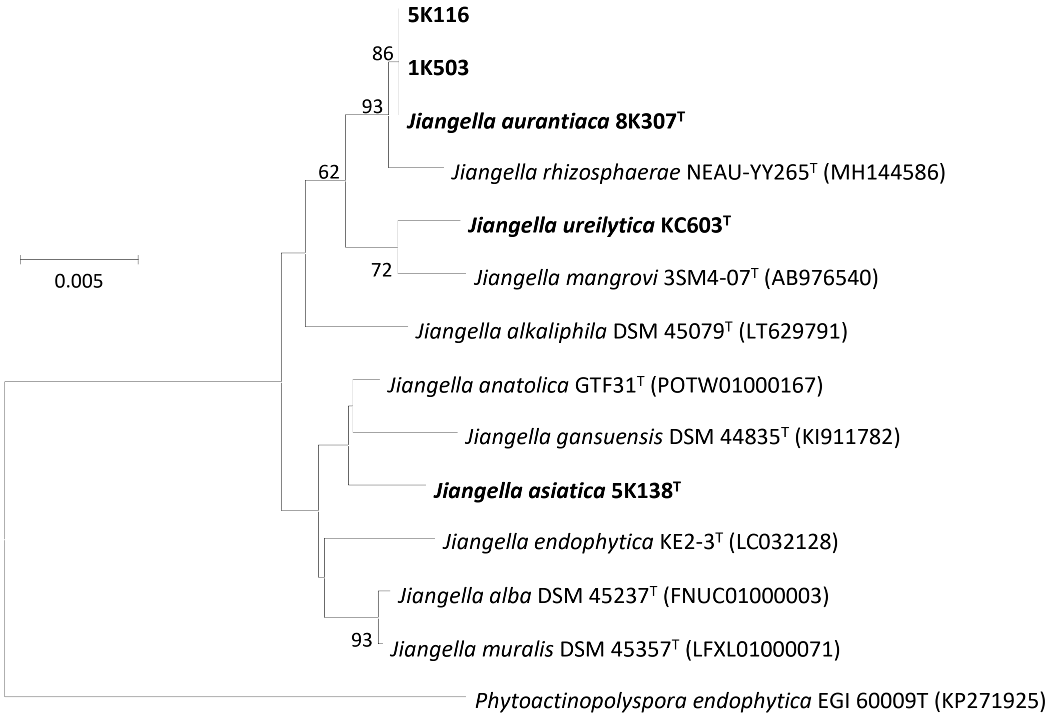

Fig. S3. Neighbor-joining phylogenetic tree based on 16S rRNA gene sequences showing relationships between isolates and closely related type strains of the genus *Jiangella*. Numbers at the nodes indicate percentage levels of bootstrap support; only values over 50 % are shown. There were a total of 1440 positions in the final dataset. Bar, 0.005 substitutions per nucleotide position. The tree is rooted using the type strain of *Phytoactinopolyspora endophytica*, the type species of the genus.

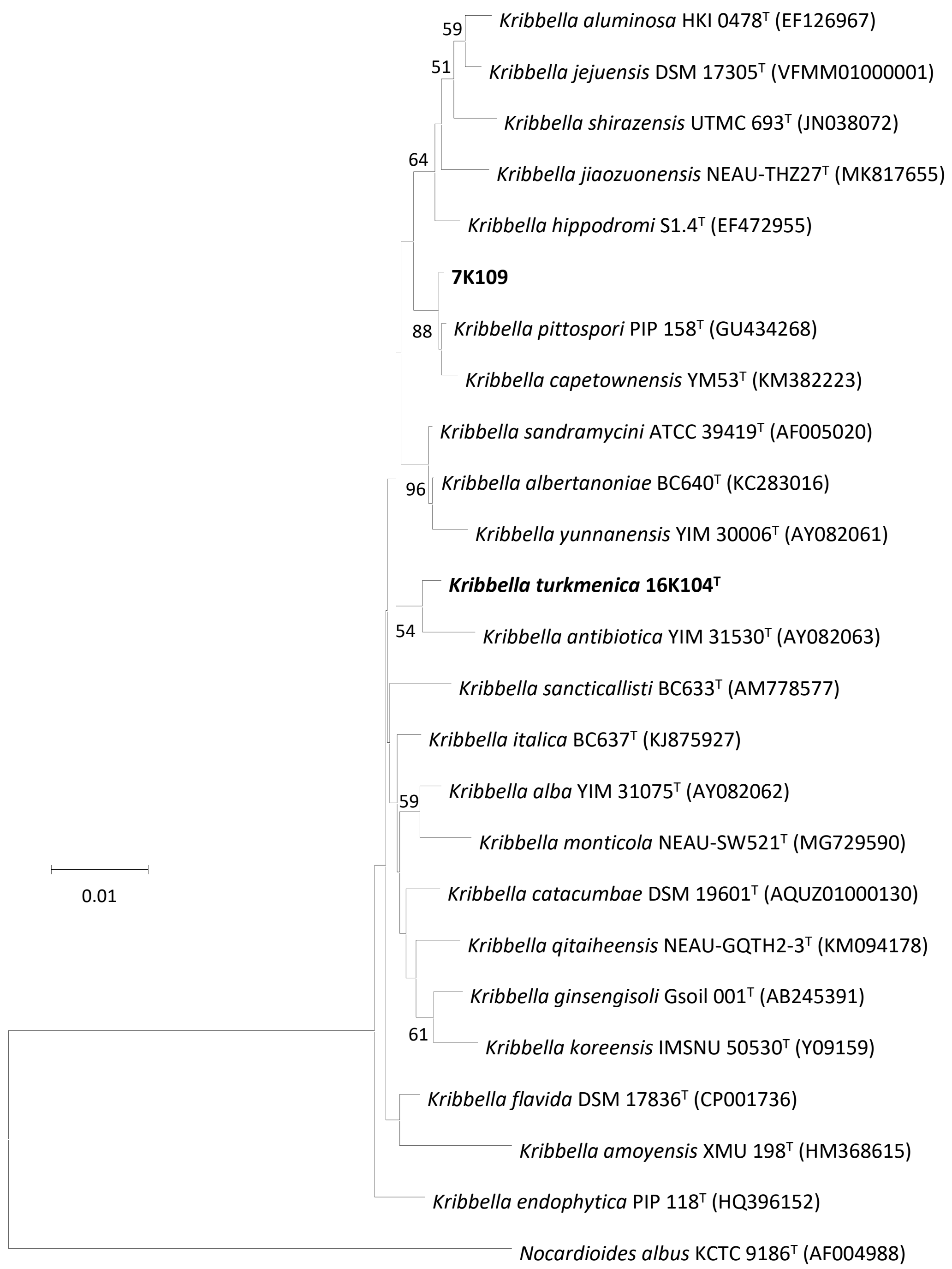

Fig. S4. Neighbor-joining phylogenetic tree based on 16S rRNA gene sequences showing relationships between isolates and closely related type strains of the genus *Kribbella*. Numbers at the nodes indicate percentage levels of bootstrap support; only values over 50 % are shown. There were a total of 1334 positions in the final dataset. Bar, 0.01 substitutions per nucleotide position. The tree is rooted using the type strain of *Nocardioides albus*, the type species of the genus.

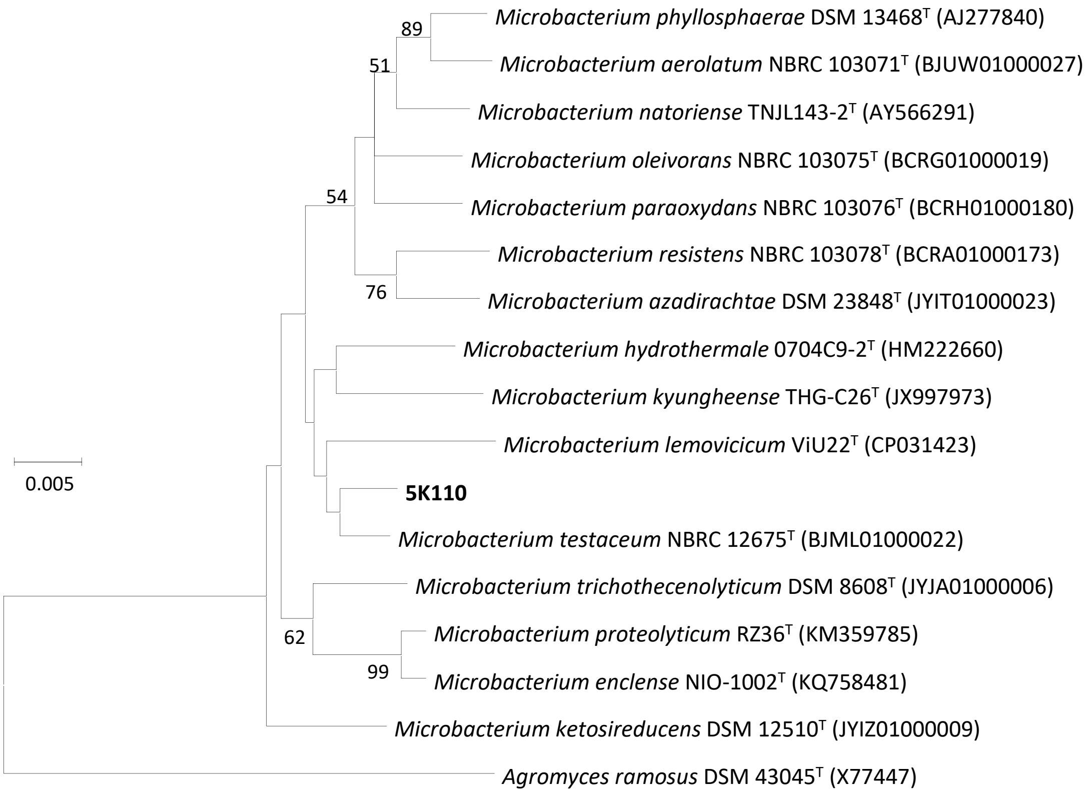

Fig. S5. Neighbor-joining phylogenetic tree based on 16S rRNA gene sequences showing relationships between isolate 5K110 and closely related type strains of the genus *Microbacterium*. Numbers at the nodes indicate percentage levels of bootstrap support; only values over 50 % are shown. There were a total of 1390 positions in the final dataset. Bar, 0.005 substitutions per nucleotide position. The tree is rooted using the type strain of *Agromyces ramosus*, the type species of the genus.

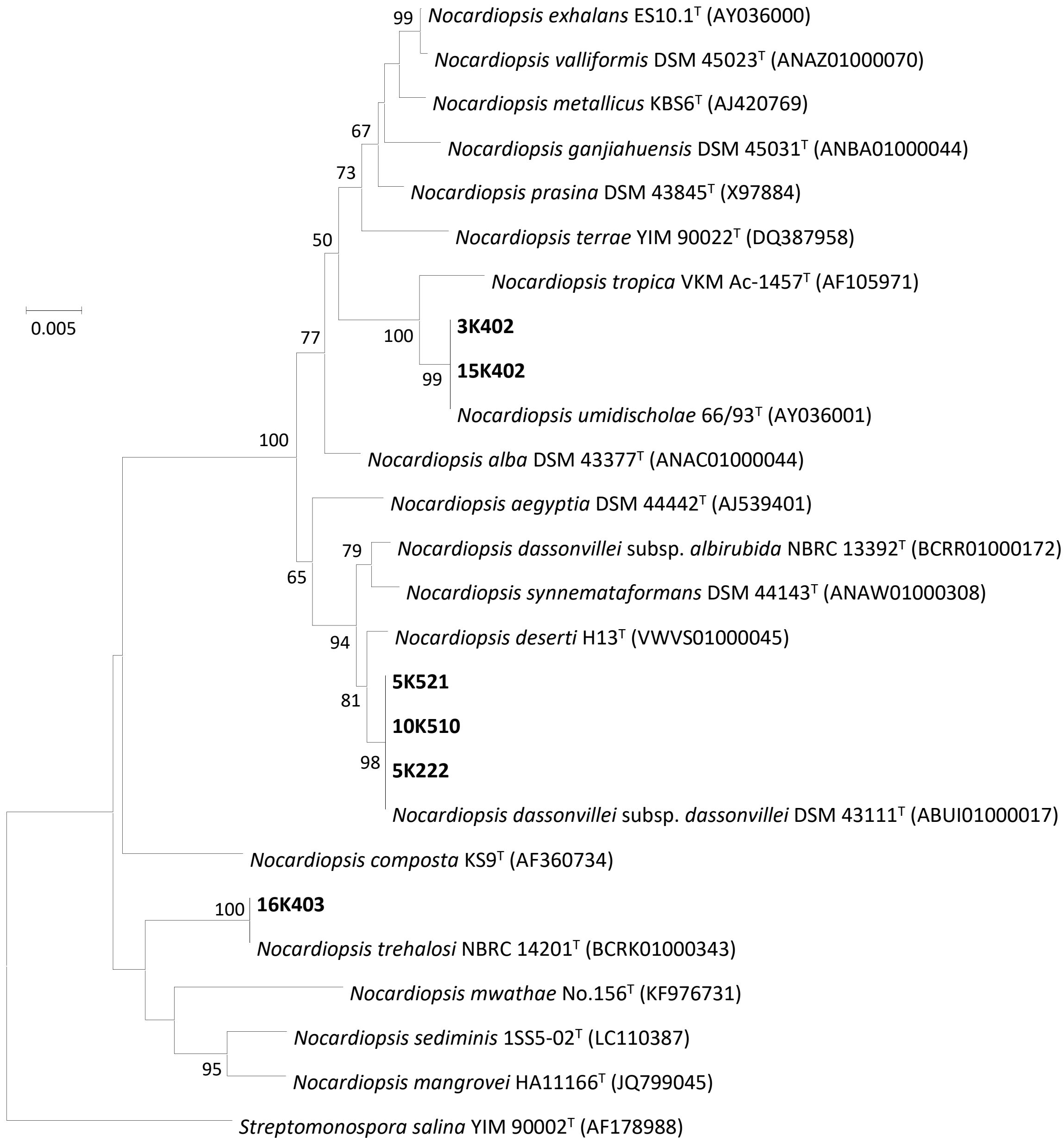

Fig. S6. Neighbor-joining phylogenetic tree based on 16S rRNA gene sequences showing relationships between isolates and closely related type strains of the genus *Nocardiopsis*. Numbers at the nodes indicate percentage levels of bootstrap support; only values over 50 % are shown. There were a total of 1408 positions in the final dataset. Bar, 0.005 substitutions per nucleotide position. The tree is rooted using the type strain of *Streptomonospora salina,* the type species of the genus.

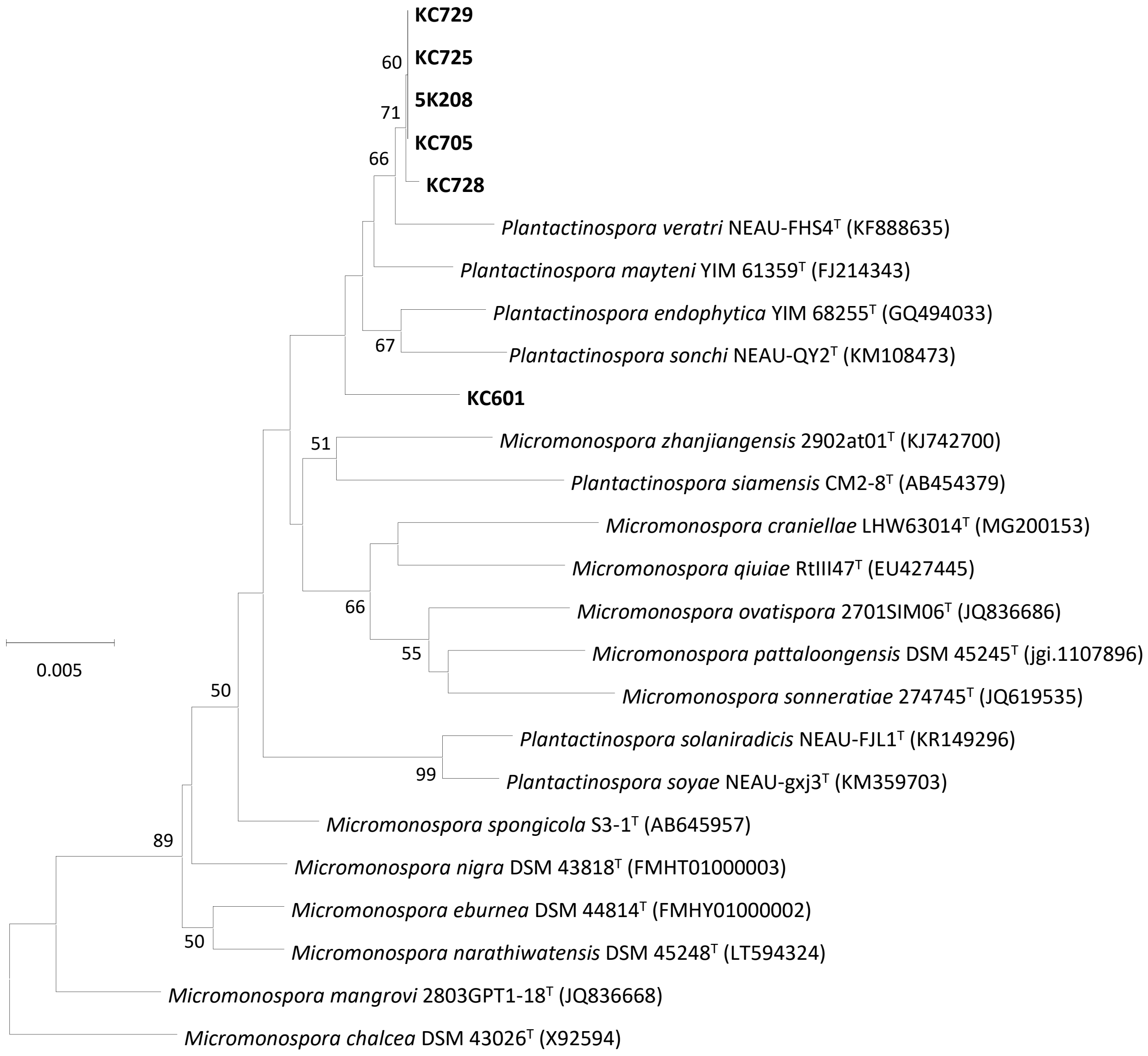

Fig. S7. Neighbor-joining phylogenetic tree based on 16S rRNA gene sequences showing relationships between isolates and closely related type strains of the genera *Plantactinospora* and closely related *Micromonospora* strains. Numbers at the nodes indicate percentage levels of bootstrap support; only values over 50 % are shown. There were a total of 1370 positions in the final dataset. Bar, 0.005 substitutions per nucleotide position. The tree is rooted using the type strain of *Micromonospora chalcea*, the type species of the genus.

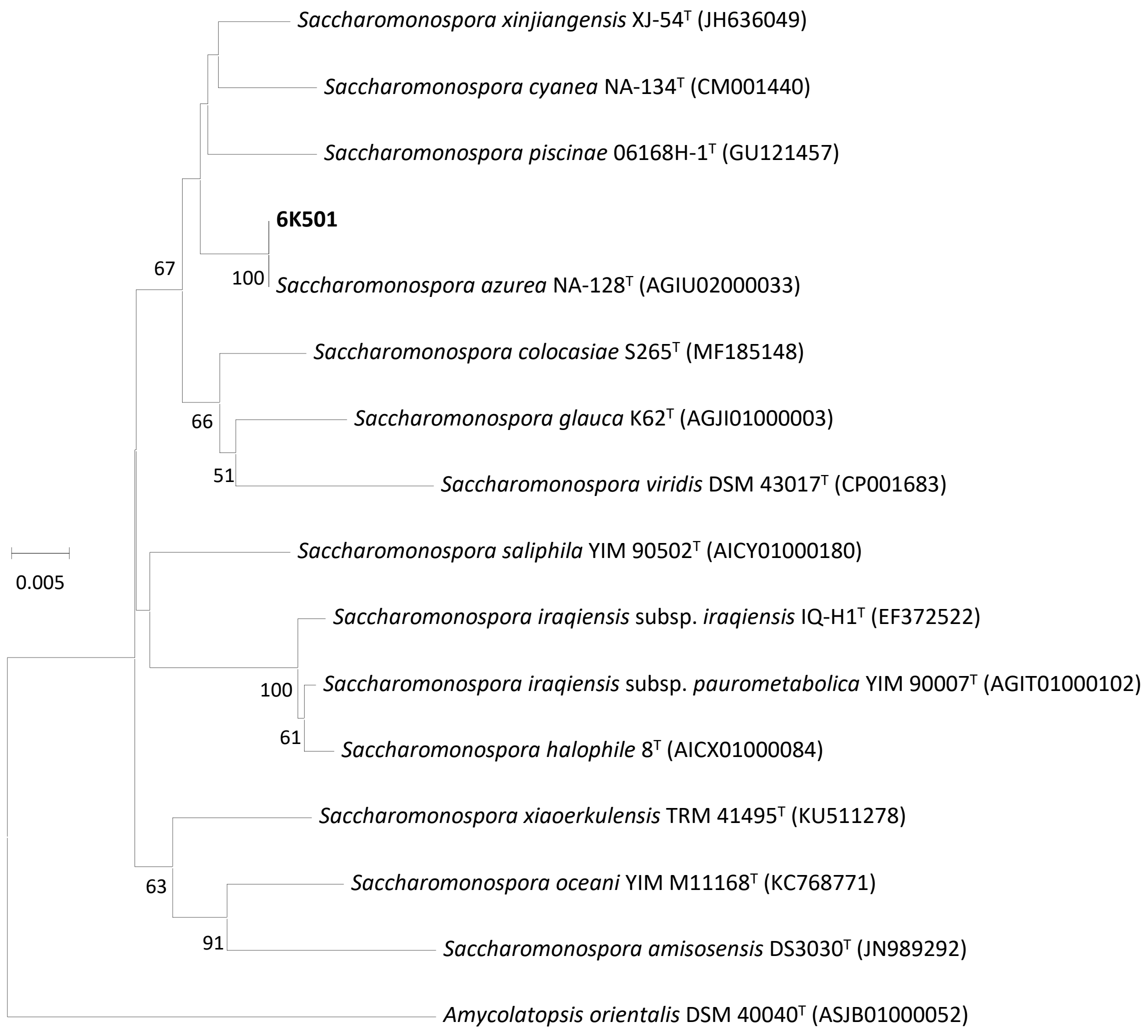

Fig. S8. Neighbor-joining phylogenetic tree based on 16S rRNA gene sequences showing relationships between isolate 6K501 and closely related type strains of the genus *Saccharomonospora*. Numbers at the nodes indicate percentage levels of bootstrap support; only values over 50 % are shown. There were a total of 1363 positions in the final dataset. Bar, 0.005 substitutions per nucleotide position. The tree is rooted using the type strain of *Amycolatopsis orientalis*, the type species of the genus.

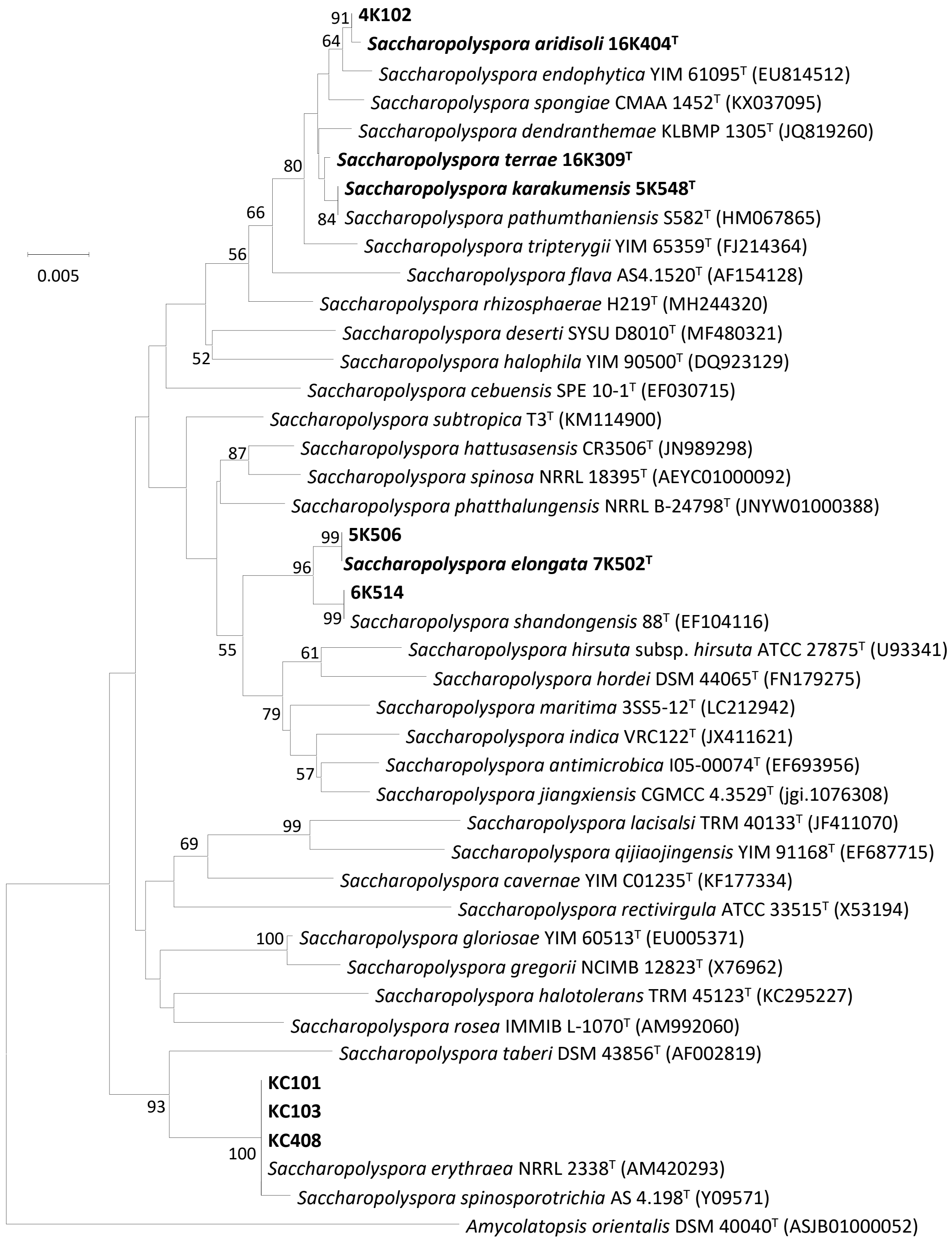

Fig. S9. Neighbor-joining phylogenetic tree based on 16S rRNA gene sequences showing relationships between isolates and closely related type strains of the genus *Saccharopolyspora*. Numbers at the nodes indicate percentage levels of bootstrap support; only values over 50 % are shown. There were a total of 1270 positions in the final dataset. Bar, 0.005 substitutions per nucleotide position. The tree is rooted using the type strain of *Amycolatopsis orientalis,* the type species of the genus.

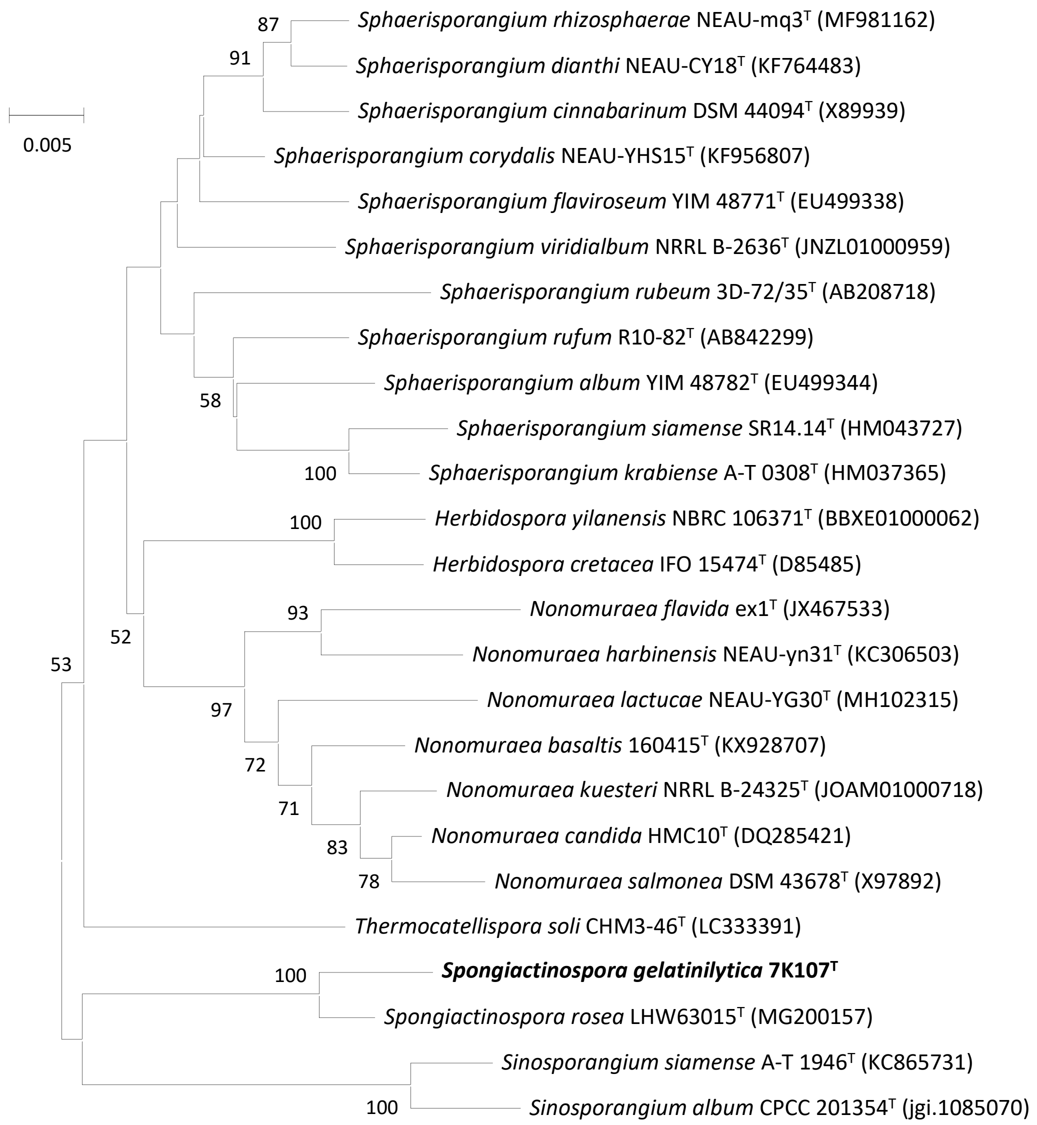

Fig. S10. Neighbor-joining phylogenetic tree based on 16S rRNA gene sequences showing relationships between isolate 7K107 and closely related type strains of the family *Streptosporangiaceae*. There were a total of 1316 positions in the final dataset. Numbers at the nodes indicate percentage levels of bootstrap support; only values over 50 % are shown. Bar, 0.005 substitutions per nucleotide position.

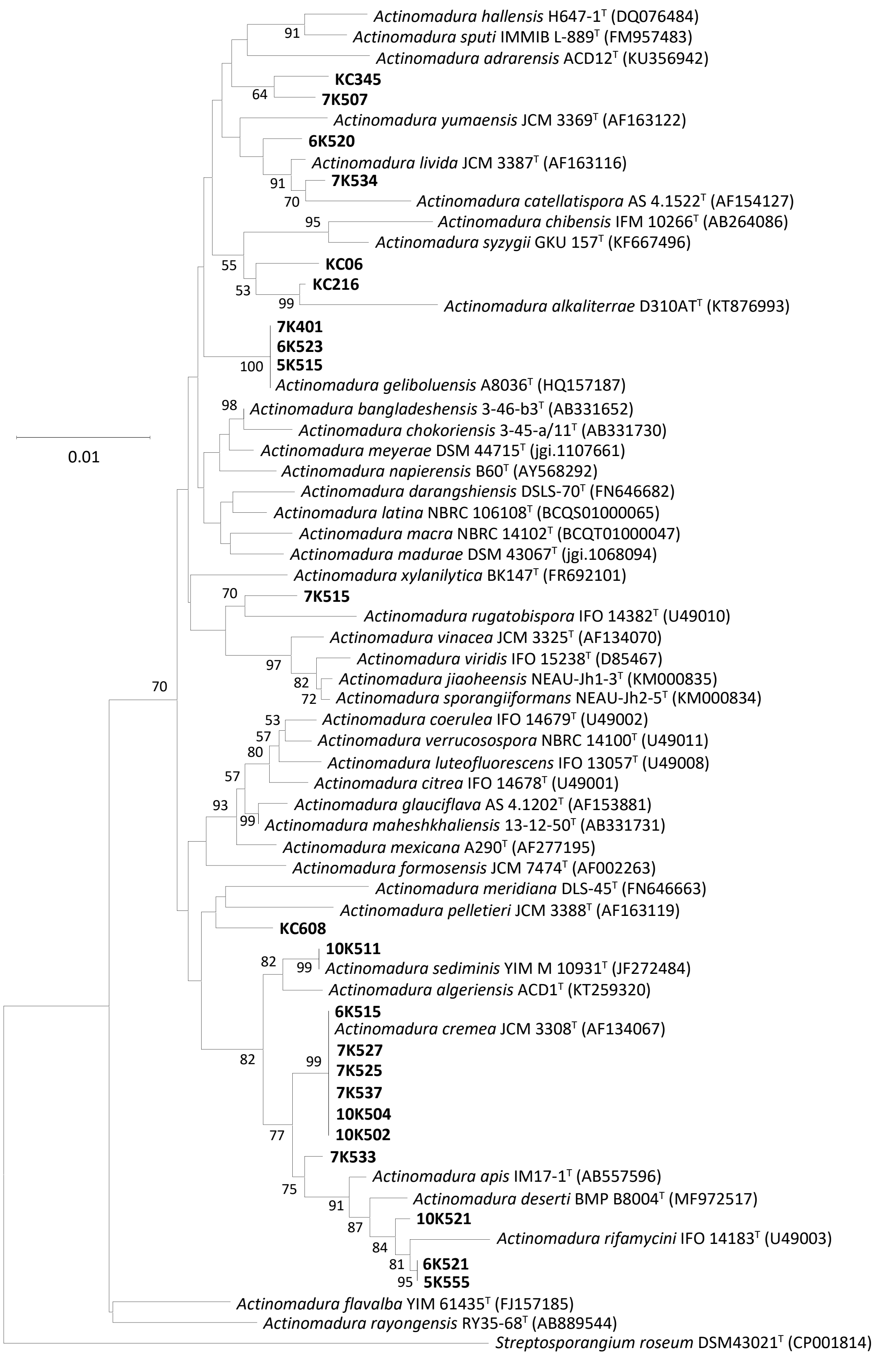

Fig. S11. Neighbor-joining phylogenetic tree based on 16S rRNA gene sequences showing relationships between isolates and closely related type strains of the genus *Actinomadura*. Numbers at the nodes indicate percentage levels of bootstrap support; only values over 50 % are shown. There were a total of 1287 positions in the final dataset. Bar, 0.005 substitutions per nucleotide position. The tree is rooted using the type strain of *Streptosporangium roseum*, the type species of the genus.

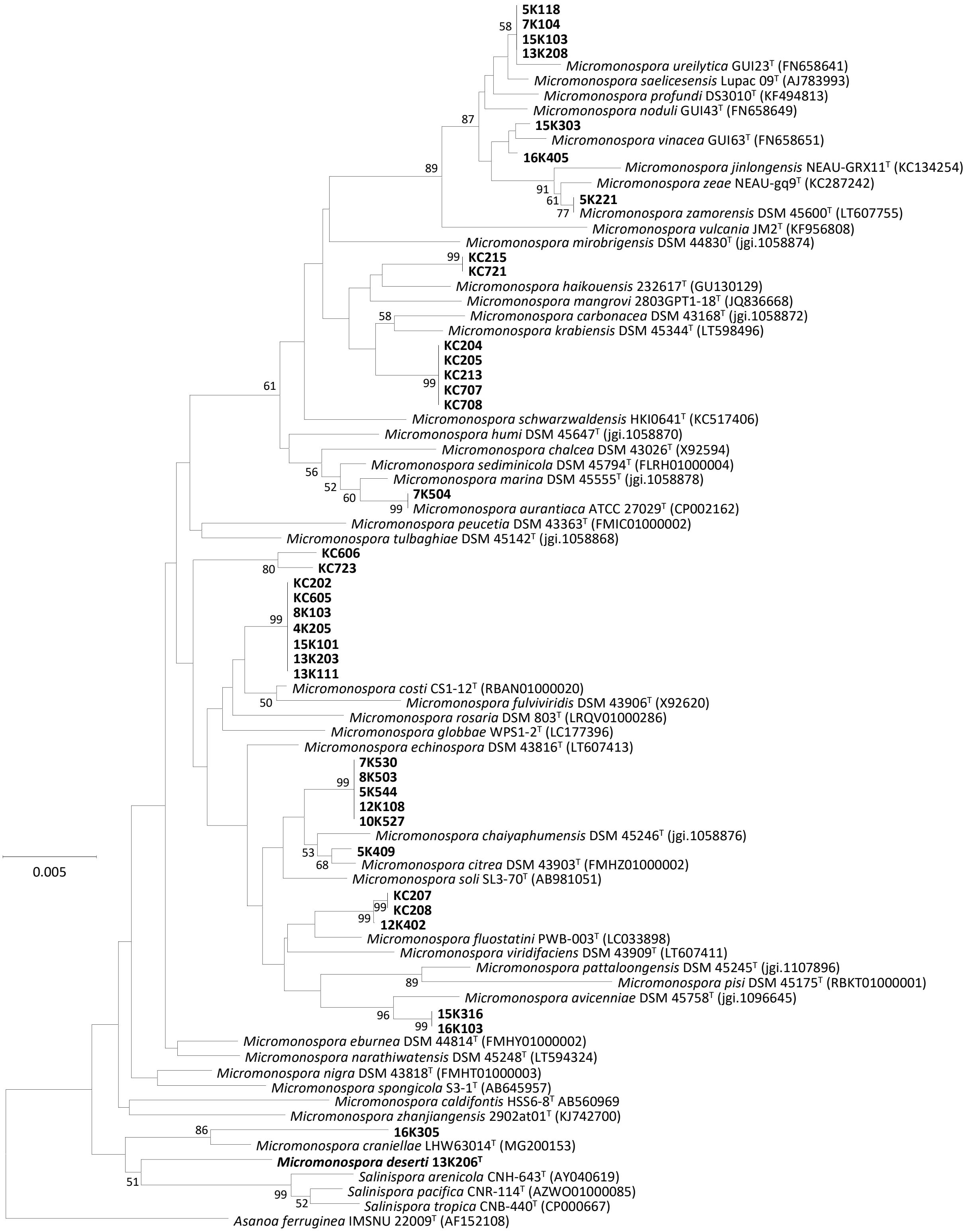

Fig. S12. Neighbor-joining phylogenetic tree based on 16S rRNA gene sequences showing relationships between isolates and closely related type strains of the genus *Micromonospora*. Numbers at the nodes indicate percentage levels of bootstrap support; only values over 50 % are shown. There were a total of 1380 positions in the final dataset. Bar, 0.005 substitutions per nucleotide position. The tree is rooted using the type strain of *Asanoa ferruginea*, the type species of the genus.

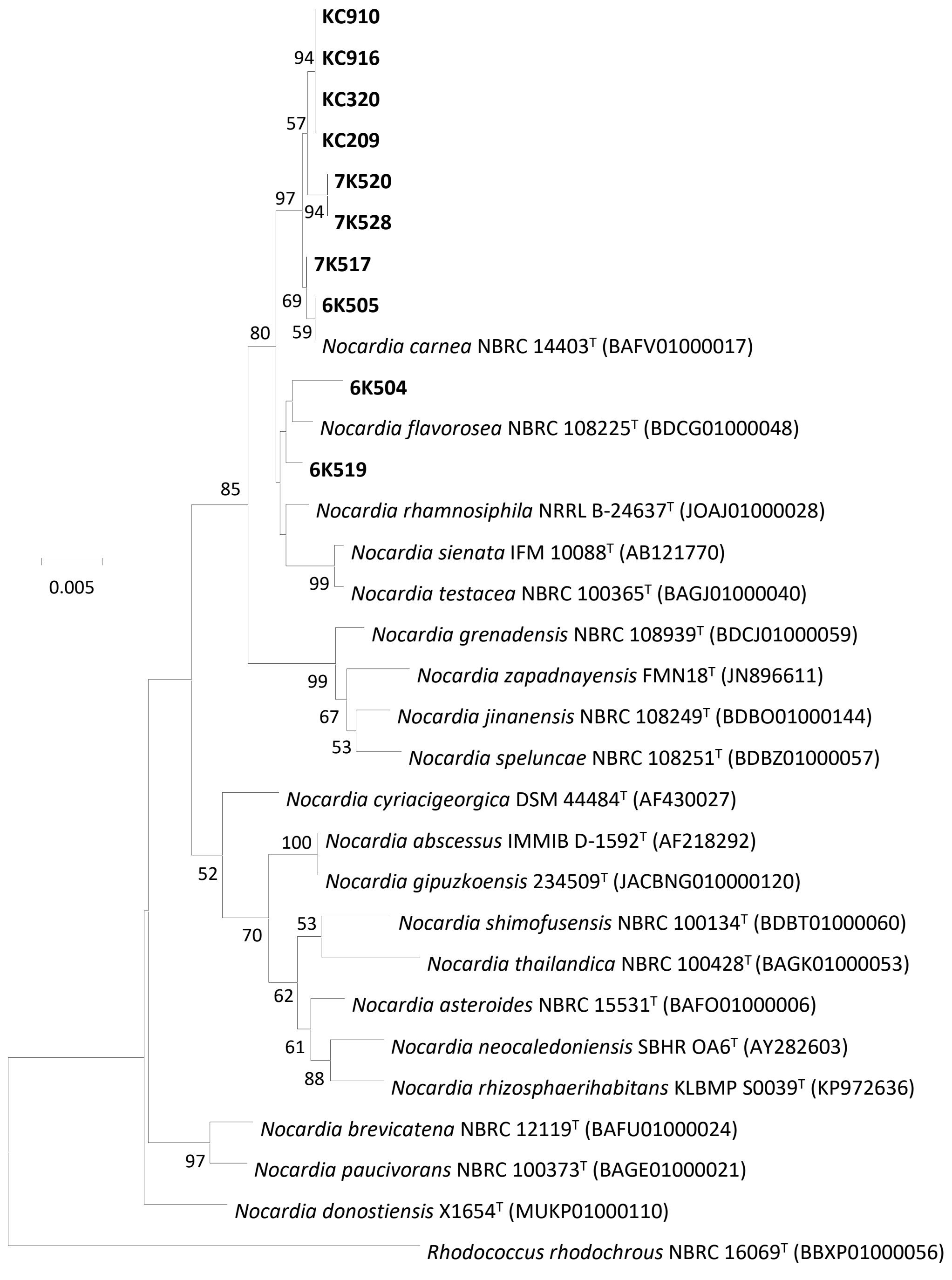

Fig. S13. Neighbor-joining phylogenetic tree based on 16S rRNA gene sequences showing relationships between isolates and closely related type strains of the genus *Nocardia*. Numbers at the nodes indicate percentage levels of bootstrap support; only values over 50 % are shown. There were a total of 1375 positions in the final dataset. Bar, 0.005 substitutions per nucleotide position. The tree is rooted using the type strain of *Rhodococcus rhodochrous*, the type species of the genus.

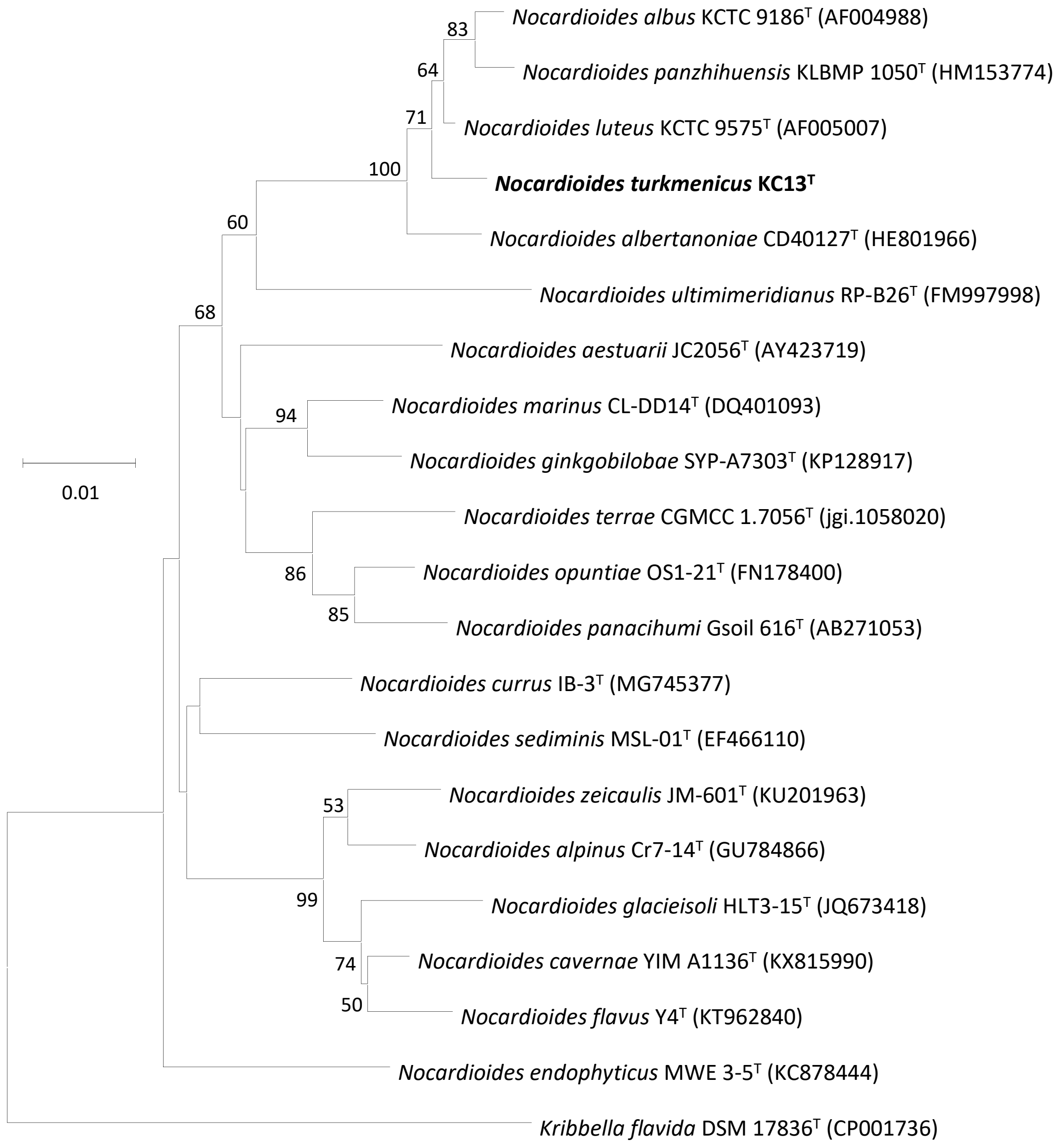

Fig. S14. Neighbor-joining phylogenetic tree based on 16S rRNA gene sequences showing relationships between isolate KC13^T and closely related type strains of the genus *Nocardioides*. Numbers at the nodes indicate percentage levels of bootstrap support; only values over 50 % are shown. There were a total of 1332 positions in the final dataset. Bar, 0.01 substitutions per nucleotide position. The tree is rooted using the type strain of *Kribbella flavida*, the type species of the genus.

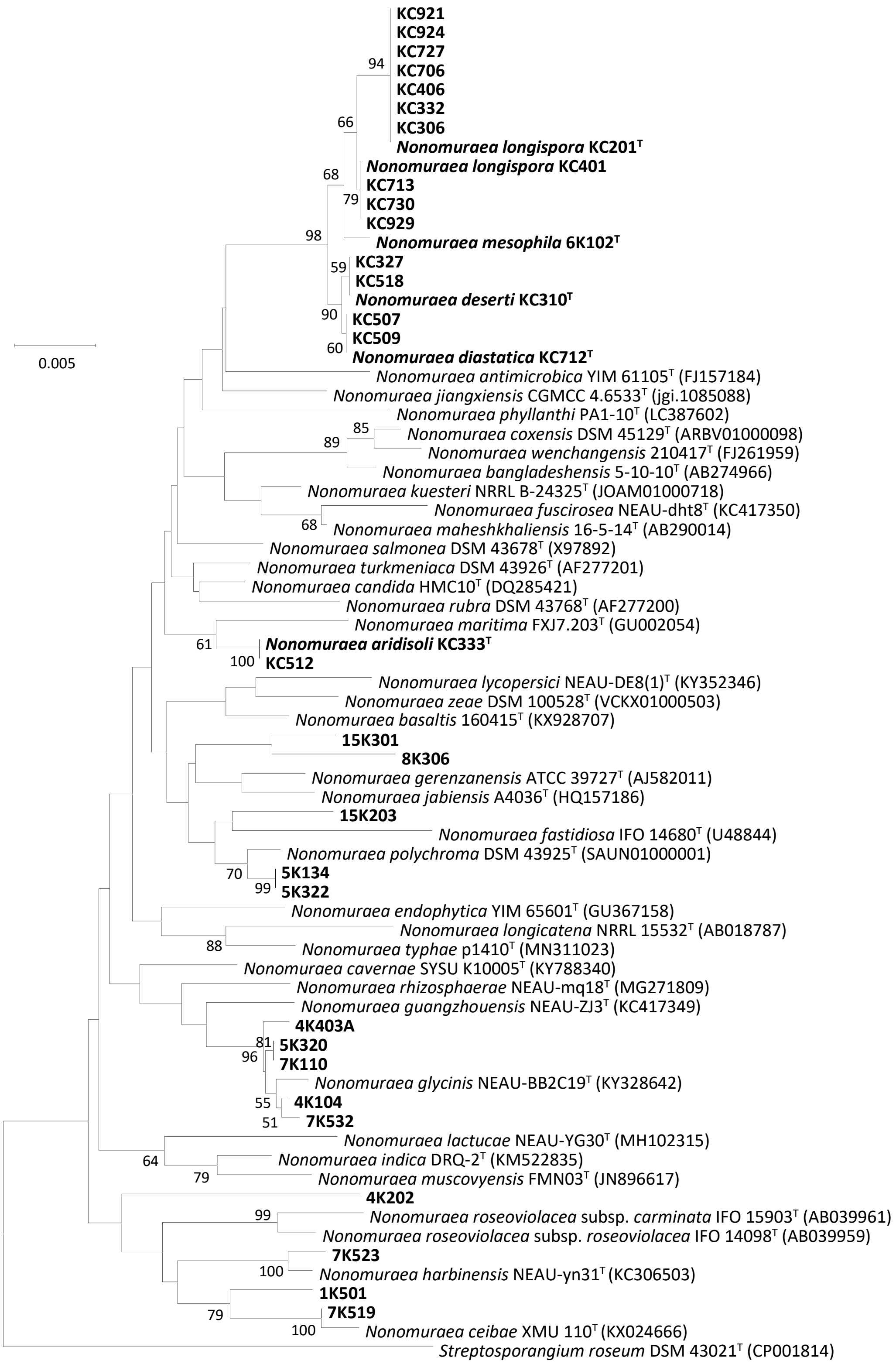

Fig. S15. Neighbor-joining phylogenetic tree based on 16S rRNA gene sequences showing relationships between isolates and closely related type strains of the genus *Nonomuraea*. Numbers at the nodes indicate percentage levels of bootstrap support; only values over 50 % are shown. There were a total of 1305 positions in the final dataset. Bar, 0.005 substitutions per nucleotide position. The tree is rooted using the type strain of *Streptosporangium roseum*, the type species of the genus.

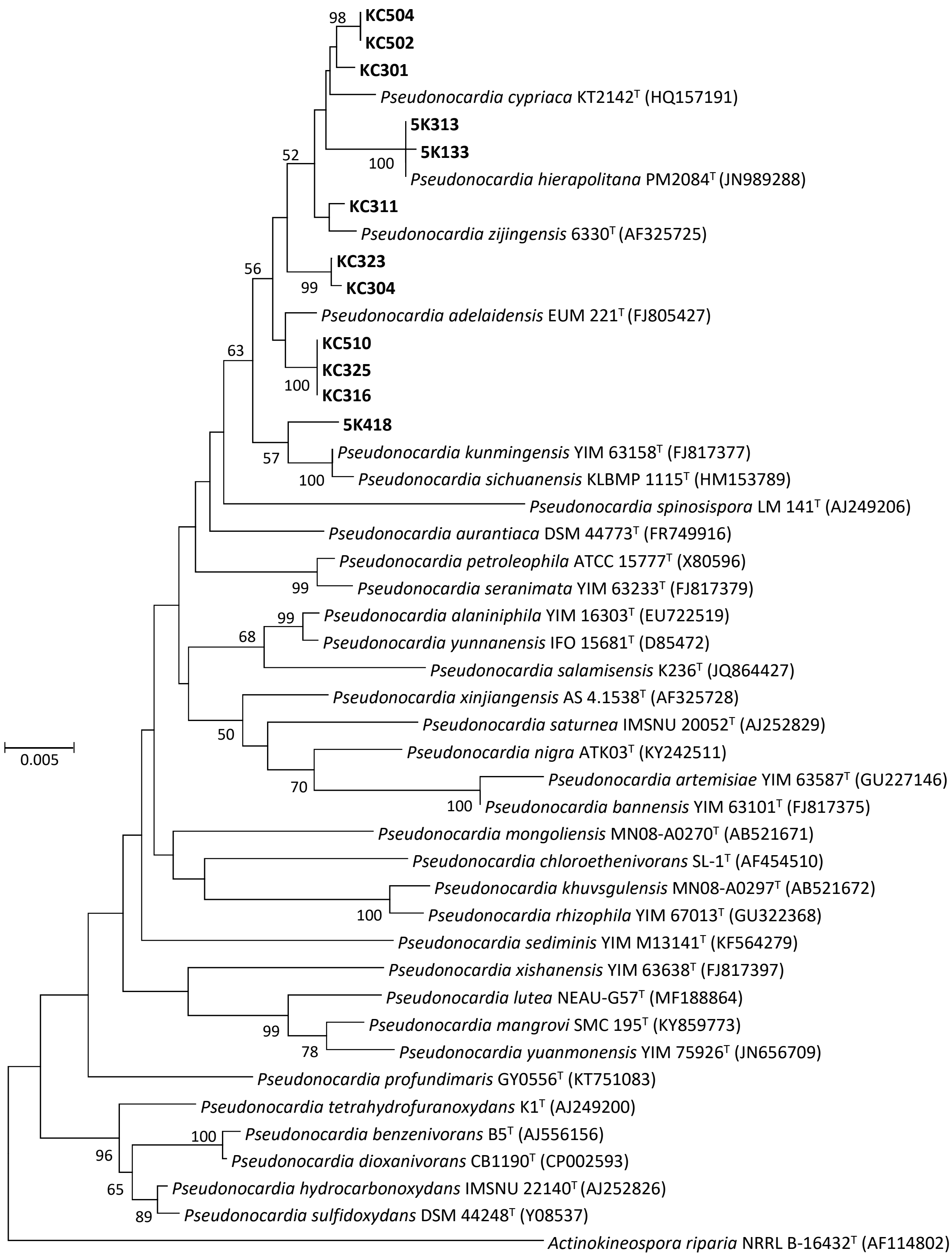

Fig. S16. Neighbor-joining phylogenetic tree based on 16S rRNA gene sequences showing relationships between isolates and closely related type strains of the genus *Pseudonocardia*. Numbers at the nodes indicate percentage levels of bootstrap support; only values over 50 % are shown. There were a total of 1385 positions in the final dataset. Bar, 0.005 substitutions per nucleotide position. The tree is rooted using the type strain of *Actinokineospora riparia,* the type species of the genus.
